# Modeling Formamide Denaturation of Probe-Target Hybrids for Improved Microarray Probe Design in Microbial Diagnostics

**DOI:** 10.1371/journal.pone.0043862

**Published:** 2012-08-27

**Authors:** L. Safak Yilmaz, Alexander Loy, Erik S. Wright, Michael Wagner, Daniel R. Noguera

**Affiliations:** 1 Department of Biochemistry and Molecular Pharmacology, University of Massachusetts Medical School, Worcester, Massachusetts, United States of America; 2 Department of Civil and Environmental Engineering, University of Wisconsin-Madison, Madison, Wisconsin, United States of America; 3 Department of Microbial Ecology, Vienna Ecology Center, Faculty of Life Sciences, University of Vienna, Wien, Austria; University of North Carolina at Charlotte, United States of America

## Abstract

Application of high-density microarrays to the diagnostic analysis of microbial communities is challenged by the optimization of oligonucleotide probe sensitivity and specificity, as it is generally unfeasible to experimentally test thousands of probes. This study investigated the adjustment of hybridization stringency using formamide with the idea that sensitivity and specificity can be optimized during probe design if the hybridization efficiency of oligonucleotides with target and non-target molecules can be predicted as a function of formamide concentration. Sigmoidal denaturation profiles were obtained using fluorescently labeled and fragmented 16S rRNA gene amplicon of *Escherichia coli* as the target with increasing concentrations of formamide in the hybridization buffer. A linear free energy model (LFEM) was developed and microarray-specific nearest neighbor rules were derived. The model simulated formamide melting with a denaturant *m*-value that increased hybridization free energy (ΔG°) by 0.173 kcal/mol per percent of formamide added (v/v). Using the LFEM and specific probe sets, free energy rules were systematically established to predict the stability of single and double mismatches, including bulged and tandem mismatches. The absolute error in predicting the position of experimental denaturation profiles was less than 5% formamide for more than 90 percent of probes, enabling a practical level of accuracy in probe design. The potential of the modeling approach for probe design and optimization is demonstrated using a dataset including the 16S rRNA gene of *Rhodobacter sphaeroides* as an additional target molecule. The LFEM and thermodynamic databases were incorporated into a computational tool (ProbeMelt) that is freely available at http://DECIPHER.cee.wisc.edu.

## Introduction

The field of microbial ecology aims to resolve the composition of complex microbial communities in engineered and natural ecosystems, with the ultimate goal of establishing the link between community structure and function. To this end, microarrays can be quite effective in determining community composition as they allow the simultaneous capture of the different types of a marker molecule (typically a functional gene or rRNA) in complex target mixtures using a large set of organism- and group-specific single-stranded DNA probes [1]. Besides traditional low throughput techniques such as Sanger sequencing of clone libraries [2] and fluorescence in situ hybridization (FISH) [3], as well as the recently established high throughput sequencing approaches [4], microarrays are an important component of the microbial ecologist’s molecular toolbox. However, the routine use of microarrays for diagnostic applications is challenged by the difficulty of designing thousands of oligonucleotide probes with optimal sensitivity and specificity to phylogenetic markers.

Probe optimization is complicated by the overwhelming diversity of microorganisms as observed with the sequence databases of small subunit rRNA, the most commonly used phylogenetic marker [5], [6], [7]. While probes in the longer range (>30 nucleotides) can generally assure sensitivity by efficient target capture, they cause specificity problems in two ways. First, due to within-group sequence variability, the longer the target site, the poorer the coverage of the probe over its targeted group of organisms (e.g., a species or a genus). Second, the higher affinity of long probes to their target molecules undermines their ability to discriminate the perfectly matching target sequences of interest from mismatching out-group sequences, thereby causing false positive identifications. Oligonucleotide probes on microarrays targeting rRNA (genes) are thus mostly in the shorter size range (<30 nucleotides). However, using shorter probes with lowered affinity can obviously cause sensitivity problems due to inefficient target capture, leading to false negatives. Therefore, in microbial ecology applications of microarrays, probe design and optimization of hybridization conditions require establishing a delicate balance between sensitivity and specificity in the oligonucleotide size range.

Since the accurate prediction of probe sensitivity and specificity is difficult [8], earlier studies with spotted microarrays relied on experimental evaluations of probes. Single targets from culture collections or clone libraries hybridized on separate microarrays were used as references to verify the relationship between probe response and organism identification in environmental samples [9], [10], [11], [12]. Although tedious, empirical testing of almost every individual probe was feasible due to the small enough number of probes (tens to hundreds) on such microarrays. However, advanced high-density microarray technology currently allows the synthesis of thousands to millions of probe features on the same slide (e.g., http://www.nimblegen.com/, http://www.affymetrix.com). While this has brought the great advantage of using more comprehensive probe sets, as in the design of 16S rRNA-based microarrays for the identification of large numbers of different phylogenetic groups of microorganisms [13], [14], [15], [16], experimental testing of all probes is no longer an option. Rather, in addition to using standard mismatch probes as in Affymetrix setups [15], [17], which are not necessarily adequate controls for cross hybridization [18], high-density microarray applications rely on the ability to design multiple probes for each taxonomic group to reduce the chance of misidentification. Certainly, it is still desirable to develop a robust strategy for the design of the individual probes with optimal sensitivity and specificity, thus increasing the accuracy of identifications based on organism-specific probe sets. We are therefore interested in establishing stringent and predictable hybridization conditions to maximize the confidence in the analyses of microbial communities.

In this study, we propose the methodical use of formamide during microarray hybridizations to develop design rules for the optimization of probe sensitivity and specificity. Formamide is a denaturant routinely used in hybridization techniques to adjust stringency [19], [20], [21]. As formamide concentration in the hybridization buffer is increased, probe/target duplexes denature, usually resulting in a sigmoidal decrease in signal response and generating a so-called melting curve [22], [23]. Since the denaturation proceeds more rapidly for mismatched duplexes than for perfect matches, there is generally an optimal range of formamide concentration that effectively eliminates signal response from mismatched non-target organisms while maintaining high signal for still non-denatured perfect match targets. Unlike other hybridization techniques, systematic evaluations of formamide denaturation are not available for microarrays, although preliminary formamide series during hybridization have been reported [24]. We show here that sigmoidal formamide melting profiles can be obtained with microarray probes, as in FISH [20], [23]. For this approach to be effective in probe design, one needs to be able to predict formamide denaturation and determine the optimal concentration range for mismatch discrimination. Thus, we also use equilibrium thermodynamics to develop a linear free energy model (LFEM) of formamide melting [23], [25] and employ this model to systematically derive thermodynamic parameters that characterize the stability of both perfect match and mismatched duplexes. Our analysis shows that the predictive ability of microarray LFEM is much better than similar models devised for FISH [23]. When combined with the multiple-probe strategy in high-density arrays, the overall approach can potentially facilitate the optimization of probe sensitivity and specificity for the high-confidence identification of organisms in complex microbial communities.

## Methods

### Targets and Target Labeling

Single 16S rRNA gene clones of *Escherichia coli K-12* and *Rhodobacter sphaeroides 2.4.1* were used as pure target templates. A small subunit rRNA gene clone library was developed and sequenced to determine the clones retrieved from the rRNA operons that encoded for the sequences used in probe design (see below). Briefly, plasmid inserts of clones were obtained from pure cultures by cell-PCR amplification with 27f [26] and 1492r [27] primers, followed by ligation and transformation with the TOPO10 cloning kit and TOP10 competent cells (Invitrogen, Carlsbad, CA). The insert was amplified with M13 primers and purified using Ampure (Agencourt Bioscience Corporation, Beverly, MA). The purified product was sequenced (primed with 27f) at the University of Wisconsin Biotechnology center using Sanger’s method. Partial sequences (ca. 800 nucleotides) were used to match sequences to known rRNA operons and one clone that matched the design template was selected for each organism.

For target labeling, the cloned and purified 16S rRNA gene was first re-amplified with the 27f and 1492r primers, and the product was purified using a QIAquick spin column (Qiagen, Valencia, CA) and Cy3-labeled according to a previously published protocol [11]. Briefly, Cy3-dCTPs (Amersham, GE Healthcare; Little Chalfont; UK) were incorporated into 200 ng PCR product during random prime amplification with Klenow fragment and a decalabel DNA labeling kit (Fermentas, St Leon-Rot, Germany). The product of labeling was purified with a QIAquick spin column and the yield was measured using a Nanodrop 1000 spectrophotometer (NanoDrop Products, Wilmington, DE). The target concentrations were in the range of 25–35 ng/µL, with an incorporated dye concentration of 0.8–1.2 ng/µL. The applied labeling procedure results in a fragmented target due to the linear random priming amplification. This was confirmed by measuring the labeled product length with an Agilent RNA 6000 Pico Kit (Agilent, Santa Clara, CA), which showed lengths ranging between 25 and 150 bases, with an average of 65 bases.

### Microarrays and Probes

High-density 4-plex microarray slides were obtained from Nimblegen (Madison, WI). Each of the four subarrays accommodated 72,000 features. Most probes were replicated three times on the array, with a total of ∼24,000 independent probe sequences produced, of which, 15,394 were used in this study (Table 1). For designing probes targeting *E. coli* and *R. sphaeroides* (Table 1), rRNA gene sequences with accession codes U00006 and X53853 were used, respectively. A poly-T chain of 20 bases was added to the 3′ end of the probe sequence, to provide an elevation above the slide surface in addition to the default linker of Nimblegen design. This was done to minimize the brush effect due to the surface-proximal tails of target molecules, which may reduce signal intensity in ways difficult to predict [28], [29], [30]. All Ts in the first three nucleotides (nearest to probe sequence) of the poly-T linker that matched an A or G in the target sequence were converted to As, to avoid the additional free energy of binding from dT-dA or dT-dG type interactions between the poly-T linker and target.

**Table 1:** Probe sets used in modeling.

Set	N^a^	Probe Length	Description	Use
TileE^b^	1380	22	22-nucleotide-long perfect match probes tiling the 16S rRNAgene of *Escherichia coli*.	Comparison to mismatches.
Length	1045^c^	18–26	Probes with varied lengths (18, 20, 24, 26) targeting 209random sites on 16S rRNA.	Models M1–M3; fitting.
OneM^b^	4140	22	TileE set with all three types of mismatches inserted in the 11th position of each probe.	Models M4, M5; fitting.
PosM	4092^d^	22	62 probes from TileE set with all types of single mismatchesinserted in all positions.	Models M4, M5; verification and positional effects.
Gap	248	22	62 probes from TileE set with a deletion at the 5^th^, 11^th^, 12^th^,or 18^th^ position.	Models M6, M7; fitting.
Insertion	248	23	62 probes from TileE set with all types of single insertion between 11^th^ and 12^th^ positions.	Models M6, M7; fitting.
TwoM	1674	22	62 probes from TileE set with all types of mismatches inserted in positions 5 and 11, 11 and 18, or 5 and 18.	Models M4, M5; verification and double-mismatch effects.
Tandem	558	22	62 probes from Tile set with all types of 2 mismatchesinserted in positions 11 and 12.	Models M8, M9; fitting.
TileR^b^	1301	22	22-nucleotide-long perfect match probes tiling the 16SrRNA gene of *Rhodobacter sphaeroides*.	Target effects; evaluation of the extent of cross hybridization.
Nonsense^e^	1	22	Nonsense sequences not complementary to targets used.	Background fluorescence

a Number of probes in set. Not all probes are directly used in model development (see next footnote, text, and Table 2).

b Probes targeting positions before the 50^th^ and after the 1450^th^ nucleotide (in *E. coli* positioning) were excluded from all analyses to avoid unamplified terminals of the targeted genes and other possible end effects.

c 209 probes shared with TileE set.

d 186 probes shared with OneM set.

e Ten replicates of the probe 5′-AGAGAGAGAGAGAGAGAGAGAG-3′.

The names, sequences, experimental signal intensity values, and calculated free energy changes of probes used in this study were deposited at the public database Gene Expression Omnibus (http://www.ncbi.nlm.nih.gov/geo/), with the accession code GSE33021, following MIAME guidelines. The naming of probes (e.g. R101–122, E1013–1034_10AC) was based on the following convention: Target (“E” for *E. coli*, “R” for *R. sphaeroides*), target site positions (5′ – 3′) on the target gene, position of mismatch from the 5′ end of the probe (if available), and the change in base (original followed by modification) to create the mismatch (if available). For deletions and inserts, “gap” and “I” preceded the mismatch position, respectively.

### Hybridization and Wash

Before hybridization, slides were pre-processed with 6–7 hrs of incubation in Nimblegen reuse buffer, a denaturing reagent that is normally used for stripping hybridized targets. The purpose of this step was to remove unknown surface-related factors that seemed to make probes less accessible at lower formamide concentrations (data not shown). For hybridization, a total of 6 or 60 ng (ca. 2 µL) of labeled and purified target was combined with 0.5 µL alignment oligo (Nimblegen), dried using a Vacufuge Plus vacuum centrifuge (Eppendorf, Hamburg, Germany) at 30°C, and then re-suspended in 10 µL of hybridization buffer (1M Na^+^, 20 mM Tris [pH = 7.2], 0.02% SDS, and variable amounts of formamide). To dissociate the complementary strands of DNA, the suspension was heat denatured by a 5-min incubation at 95°C, followed by fast cooling on ice. The hybridization buffer was then applied to the array surface using NimbleGen 4-plex mixers adhered to the slides. A total of ca. 8 µL suspension was transferred to each array, bringing the used target mass to ca. 5 or 50 ng. The slides were placed in a 12-bay NimbleGen Hybridization System for overnight (∼20 hrs) hybridization at a controlled constant temperature of 42°C, and with active mixing of the hybridization buffer to improve mass transfer.

After hybridization, slides were washed in pairs, using a series of three wash buffers (I, II, and III) provided by Nimblegen and following Nimblegen guidelines. All buffers were amended with 0.1 mM dithiothreitol according to manufacturer’s recommendations. Each slide was first submerged in 250 mL of pre-warmed (40–45°C) wash buffer I to detach the mixer from slide surface and immediately taken through the wash series in buffers I (2 min), II (1 min), and III (15 secs) at room temperature with constant manual agitation. The slides were dried using Arrayit High-Speed Microarray Centrifuge (Telechem, Silicon Valley, CA) and subsequently stored in a dark and dry environment.

### Scanning and Analysis

Microarrays were scanned with an Axon 4000B laser scanner and GenePix Pro 6.0 software (Molecular Devices, Sunnyvale, CA). The wavelength and PMT gain were set at 532 nm and 430, respectively. Two lines were averaged during scanning. Fluorescence data was saved in TIFF files, which were processed with Nimblescan software (Nimblegen). Using the signal from the alignment oligomers a custom grid was aligned with the images to derive raw data for each feature. It should be noted that this procedure produces data in the form of pixel intensity values ranging from 0 to 65536, the latter representing a saturation point. Raw data was saved as pair files and analyzed using Matlab (The MathWorks, Natick, MA). For each probe, the average and standard deviation of the brightness of three replicate features were calculated. An outlier test was also performed, such that if one of the replicates gave a value that was more than three standard deviations (of the remaining two) away from the average of the remaining two it was eliminated. Then, the average of control (Nonsense) probes (see Table 1) was subtracted from all averages to obtain background-corrected results (standard deviations were calculated with error propagation).

### Linear Free Energy Model (LFEM)

To simulate probe/target hybridization in the presence of formamide, the LFEM previously developed for FISH [23] was reduced to a two-state hybridization system describing the local equilibrium at the probe’s microenvironment (*P*+*T* = *PT*, where P, T, and PT denote probe, target, and hybrid, respectively). The modified microarray LFEM defines hybridization efficiency (i.e., the ratio of probe-bound target to all locally available: [*PT*]/[*T*]_o_) as shown in Equation 1, where, ΔG° is the free energy change for no formamide condition, the *m*–value defines the linear increase in the free energy change with increasing formamide concentration ([*FA*]) [23], [31], [32], and *R* and *T* stand for the gas constant (0.00199 kcal/molK) and hybridization temperature (315.15 K), respectively. During the derivation of Equation 1, the activity coefficients of *P*, *T*, and *PT* were added to the reaction stoichiometry, as different from the LFEM for FISH [23], and embedded in the effective probe concentration term ({P}_o_), which is treated as an unknown parameter to be derived by model-fitting (see below).
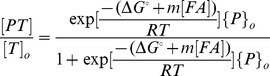
(1)


The free energy value in Equation 1 was calculated as described elsewhere [22], [33], [34], [35]. Briefly, ΔG° of perfect match hybrids was obtained by summing the free energies of all nearest neighbors and adding an initiation free energy to this sum [34], [35]. For mismatched duplexes, the free energy difference introduced by the mismatch was formulated using a ΔΔG° term described in Equation 2 [22], [33], which reflects both the destabilizing effect of losing a nearest neighbor pair (second term on the right hand side) and the contribution of the newly formed internal loop (first term on the right hand side). Both solution-based and microarray-specific parameters were used for nearest neighbor and loop terms in this study. Solution based parameters were obtained from UNAFold [36], whilst microarray parameters were derived by modeling.
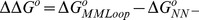
(2)


### Curve Fitting

Predicted hybridization efficiency in a formamide series was matched to normalized experimental melting profiles with Equation 3, where *I* is the background-corrected probe signal intensity at a specific formamide concentration, *I_max_* is the maximum *I* value achieved in the whole formamide series, and *γ* represents a probe-specific proportionality constant that aligns experimental and theoretical trends. Theoretical formamide curves of multiple probes were simultaneously fitted to their experimental profiles using a bi-level fitting approach. Thus, the parent fitting function changed general modeling parameters ({*P*}_o_ and the *m*-value in Equation 1 along with free energy parameters), while a secondary fitting function determined the probe-specific proportionality constants according to Equation 3 (i.e., a particular *γ* value for each probe). Curve-fitting was done via non-linear regression [37] using the ‘nlinfit’ routine in the Statistics Toolbox of MATLAB, as described previously [23]. The goodness of fit was evaluated by the coefficient of determination (*R* ^2^) in Equation 4, where *y*, *r*, and *n* represent experimental data points, residuals, and the total number of formamide data points used in the fitting, respectively. To compare the performance of different models with varied number of parameters, the error squares function (*s* ^2^) in Equation 5 was used. Here, *ν* represents the degree of freedom (i.e., *n* minus all parameters, including one *γ* value for each probe used in the fitting).
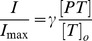
(3)

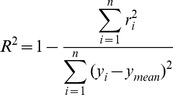
(4)

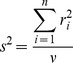
(5)


Curve fitting was based on data from modeling probe sets in Table 1 using 5 ng of *E. coli* 16S rRNA gene as the target. Experimental signal intensity values of some of these probes were close to the background over the entire formamide series or all points but 0% formamide. Since these probes were observed to bias fitting parameters by random noise, they were eliminated from curve-fitting (<15% of all probes; the final number of probes used in fitting are provided in Table 2 by the parameter *N_T_*; see below). Eliminations included perfect and mismatched probes with *I_max_* values <1000 a.u. and <500 a.u. respectively, and mismatched probes whose signal decreased by more than 50% in the first increment of the formamide series (i.e., from 0 to 5%). After model development, retrospective analyses showed that 99%, 93%, and 56% of these filtered probes were predicted to have half denaturation points (see below) at or below 15%, 10%, and 5% (v/v) formamide, respectively. Thus, filtered data was mainly a result of predictable poor hybridization efficiency and did not significantly affect modeling evaluations.

**Table 2:** Model development and curve-fitting^a^.

Model Description^b^			Parameters^c^				Statistics^d^						|err[FA]_1/2_|^e^	
Name	Type	Free Energy Change (kcal/mol)^f^	*m (kcal/mol/%)*	log{*P_o_*}	*α*	*β*	N_F_/N_T_	Σ*r^2^/n*	*ε^2^_val_*	*s^2^*	*ε^2^_ov_*	*R^2^*	*µ*/σ	<5%
M1	Perfect Match	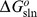	*0.522*	*−11.6*	na	na	500/1033	0.0207	0.0210	0.0240	0.0209	0.87	3/2.2	81.3
M2	Perfect Match	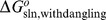	*0.528*	*−12.4*	na	na	500/1033	0.0212	0.0211	0.0246	0.0211	0.87	3/2.3	80.6
**M3**	**Perfect Match**	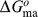	***0.173***	***−2.0***	**na**	**na**	**500/1033**	**0.0083**	**0.0080**	**0.0096**	**0.0081**	**0.95**	**2.1/1.7**	**93.4**
M4	Central Single Mismatch	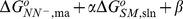	0.173	−2.0	*0.354*	*0.487*	1750/3594	0.0093	0.0091	0.0107	0.0092	0.94	2.2/1.7	92.6
**M5**	**Central Single Mismatch**	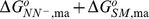	**0.173**	**−2.0**	**na**	**na**	**3594/3594**	**0.0078**	**na**	**0.0090**	**0.0078**	**0.95**	**2/1.6**	**94.5**
M4	Positional Single Mismatch	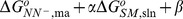	0.173	−2.0	0.354	0.487	3815/3815	na	na	0.0117	0.0101	0.93	2.4/1.8	90.2
**M5**	**Positional Single Mismatch**	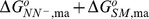	**0.173**	**−2.0**	**na**	**na**	**3815/3815**	**na**	**na**	**0.0107**	**0.0093**	**0.94**	**2.3/1.7**	**91.6**
M6	Bulged Mismatch	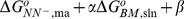	0.173	−2.0	*0.132*	*0.296*	250/467	0.0098	0.0099	0.0113	0.0098	0.94	2.4/1.8	91.4
**M7**	**Bulged Mismatch**	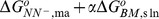	**0.173**	**−2.0**	***0.238***	**na**	**250/467**	**0.0098**	**0.0098**	**0.0113**	**0.0099**	**0.94**	**2.4/1.8**	**90.8**
M4	Two Mismatches	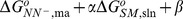	0.173	−2.0	0.354	0.487	1086/1086	na	na	0.0126	0.0111	0.92	1.4/1.4	98.1
**M5**	**Two Mismatches**	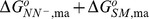	**0.173**	**−2.0**	**na**	**na**	**1086/1086**	**na**	**na**	**0.0111**	**0.0098**	**0.93**	**1.2/1.2**	**98.8**
M8	Tandem Mismatch	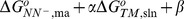	0.173	−2.0	*0.198*	*1.167*	300/401	0.0117	0.0118	0.0133	0.0117	0.92	1.6/1.5	97.3
M9	Tandem Mismatch	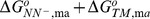	0.173	−2.0	na	na	300/401	0.0107	0.0112	0.0123	0.0108	0.92	1.5/1.3	97.8
**M9**	**Tandem Mismatch**	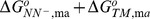	**0.173**	**−2.0**	**na**	**na**	**401/401**	**0.0108**	**na**	**0.0124**	**0.0108**	**0.92**	**1.5/1.3**	**97.5**

a Concluding (optimal) models are indicated in bold and their details are presented in other tables and figures.

b SM, single (non-bulge) mismatch; BM, bulge mismatch; TM, tandem mismatch; sln, for in solution hybridization; ma, for microarray hybridization.

c Parameters in italics are used in best-fitting. The use of additional parameters *α* and *β*, when applicable, is shown in the free energy column under model description (see text for best-fitting values). Other parameters derived using M3, M5, and M9 are the free energy rules in Tables S1A, S1B, and S1D, with 10, 104, and 8 additions, respectively.

d N_F_, number of probes used in fitting; N_T_, total number of probes; *ε* ^2^_val_ and *ε* ^2^_ov_, average squares of prediction errors in validation set (i.e., not used in fitting) and overall set, respectively. See Materials and Methods for other parameters.

e Absolute value of the deviation of predicted half-denaturation point (% formamide) from the apparent experimental value, as described by its average/standard deviation (*µ/σ*) and percentage of values below 5% formamide.

f ΔG° for perfect matches and ΔΔG° for mismatches (see Materials and Methods).

## Results

**Figure 1:**
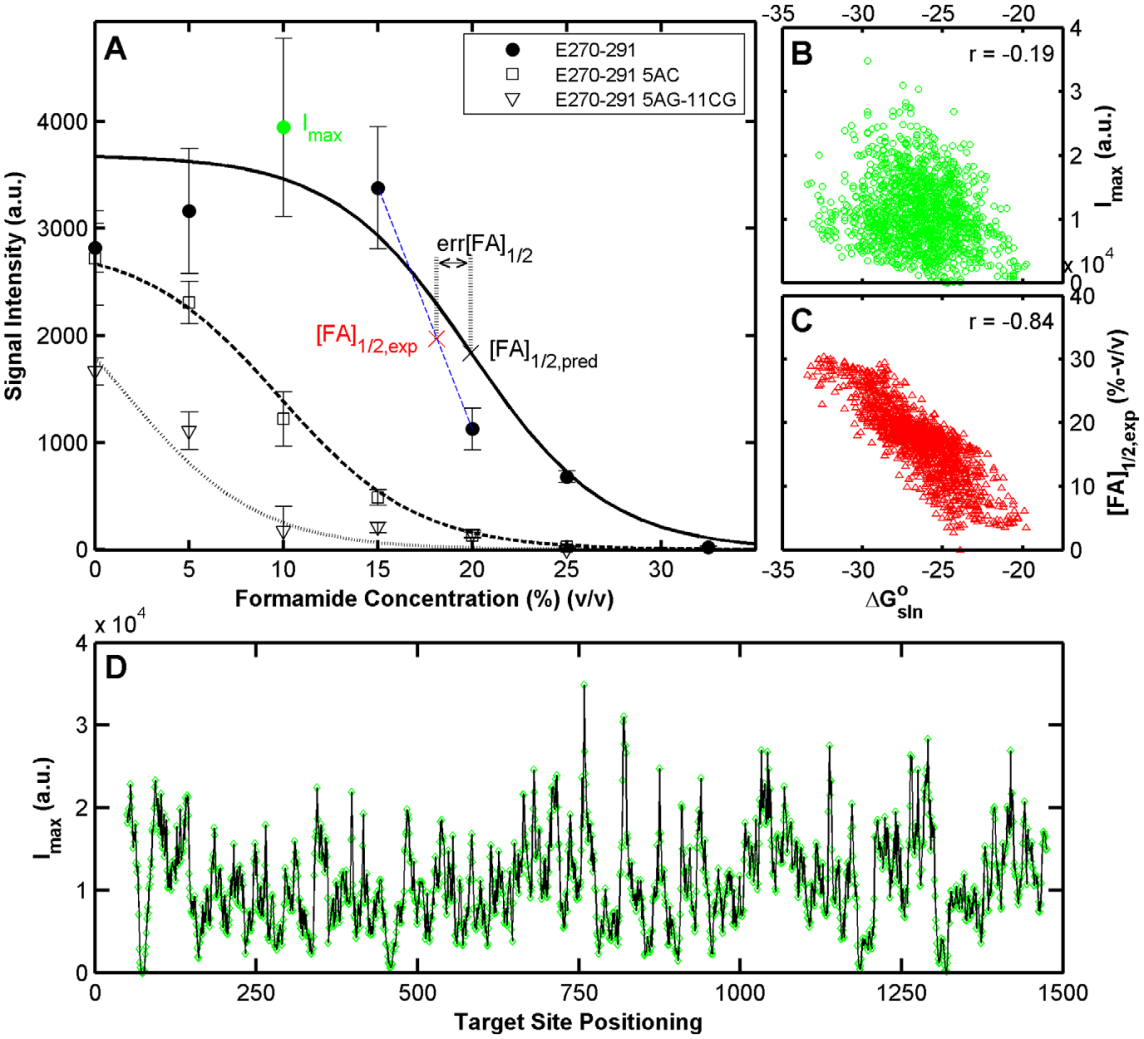
Characteristics of formamide denaturation profiles. (**A**) Example formamide curves with perfect match, one-mismatch, and two-mismatch probes targeting the same site on 16S rRNA gene of *E. coli*. Curves represent theoretical profiles. Observed maximum signal intensity (I_max_), experimental ([FA]_1/2,exp_) and predicted ([FA]_1/2,pred_) half-denaturation points, and the prediction error (err[FA]_1/2_) are illustrated. [FA]_1/2,exp_ is estimated by linear interpolation between two subsequent experimental points that are greater than and less than I_max/2_, respectively. Panels (**B**) and (**C**) show the correlation of solution-based free energy predictions with I_max_ and [FA]_1/2,exp_, respectively, with r defining the Pearson’s correlation coefficient. (**D**) I_max_ plotted against position of target site, as represented by the middle point. All data were obtained from probes belonging to the TileE set (Table 1). Amount of hybridized target was 5 ng.

We obtained probe denaturation profiles with a formamide series of eight concentrations: 0, 5, 10, 15, 20, 25, 32.5, and 45% on a volume by volume basis (v/v). For each target, this was achieved by parallel hybridizations with two slides (4 arrays per slide). Typical experimental profiles are shown in Figure 1A for selected perfectly matched and mismatched probes from the hybridization experiment with 5 ng of amplified, fragmented, and Cy3-labeled *E. coli* 16S rRNA gene used in model development. As expected, increasing formamide creates a sigmoid-like loss of signal as the efficiency of target capture decreases, and the melting occurs at lower concentrations when mismatches are inserted in the duplex (Figure 1A). For those probes with a full sigmoid trend, there is a general increase of signal with increasing formamide at lower formamide concentrations, as exemplified by the perfect match probe in Figure 1A, which may be due to the removal of structural kinetic limitations by formamide as in FISH [38], [39] or other unknown complications in microarray hybridizations. In any case, the gradual loss of signal at higher stringency creates a window of formamide concentrations (15–25% in the example in Figure 1A), where the signal from perfect match duplex is easily detectable, while the mismatched duplexes are close to the background, thereby allowing mismatch discrimination as desired. Thus, our modeling efforts aimed at predicting the observed melting behavior for probe design and optimization.

Our mathematical framework depends on the estimation of the standard Gibbs free energy change (ΔG°) of the hybridization reaction (Equation 1). Initially, we used UNAFold [36] to predict a ΔG° value based on thermodynamic parameters from in-solution hybridizations (hence designated ΔG°_sln_) and evaluated its correlation with the experimental observations. As shown in Figure 1B, this free energy value poorly correlated with maximum signal intensity of probes in a formamide series (*I_max_*). The low correlation can be attributed to non-thermodynamic factors that may influence the signal intensity of individual probes, such as the biases introduced during the amplification and fragmentation of the target (e.g., fragment concentration and dye labeling efficiency). Indeed, *I_max_* showed non-random positional dependence in the 16S rRNA gene with regions of peaks and sinks (Figure 1D), which may presumably reflect these biases. It is noteworthy that, patterns as in Figure 1D have been reported before for single target molecules [40], but could be related to binding free energy unlike with our dataset (Figure 1B). A more robust descriptor of thermodynamic stability would be the melting behavior, since positional and other non-thermodynamic factors for a given probe likely remain constant in a formamide series. Consistently, the point of half-denaturation ([FA]_1/2,exp_), defined as the formamide concentration where signal intensity decreased to half of *I_max_*, was largely predictable by ΔG°_sln_ (Figure 1C). This term represents the melting point of the duplex when a nearly full sigmoidal profile is obtained as is the case for most perfect matches used in this analysis. In retrospect, [FA]_1/2,exp_ also does not correlate with *I_max_* itself (r = −0.22; not shown), further pointing to the dependence of *I_max_* on factors not related with stability. Thus, we focused our modeling strategy on the normalized melting profiles where thermodynamically irrelevant signal variations as in Figure 1D are mostly eliminated.

**Figure 2:**
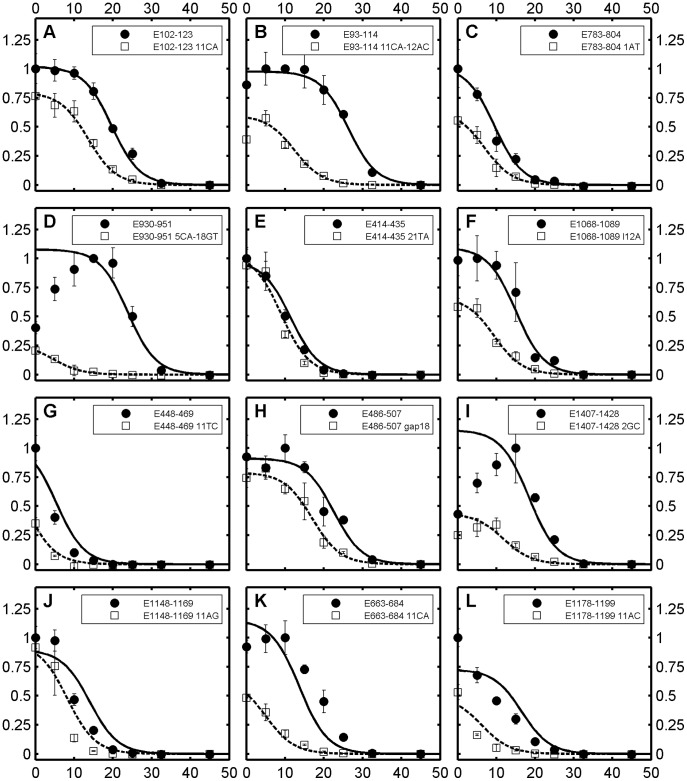
Formamide melting profiles of 12 arbitrarily selected perfect match probes and their mismatched versions. All perfect matches are from the TileE set, since only this set has mismatched versions (for examples from the Length set, see Figure S1A). Solid and dashed curves indicate theoretical profiles for perfect matches and mismatches, respectively. x-axis, formamide concentration; y-axis, normalized signal intensity; error bars, standard deviations.

Our methodology is based on simulating the formamide-based denaturation of a probe/target duplex with the linear free energy model (LFEM) in Equation 1. This defines hybridization efficiency as a sigmoidally decreasing function with increasing formamide concentration, which takes values up to 1 at low formamide and 0 at full denaturation. The resulting theoretical curves are simultaneously fitted to large sets of experimental probe profiles normalized using Equation 3 for each probe (see Figure 2 for example fits). In what follows, we describe the stepwise use of LFEM for the systematic establishment of free energy rules and calibration of formamide denaturation models for perfect and mismatched duplexes (Table 2).

### Perfect Matches

To calibrate LFEM for perfect matching duplexes, we formed a set of 1045 probes of variable length (Length set, Table 1), of which, 1,033 were used for modeling after elimination of probes close to the background (N_T_ = 1033, Table 2). This set was designed to have a wide variability of half denaturation points (range, 4–30%; median, 18%) for a robust calibration of key modeling parameters. A randomly selected subset of 500 probes (N_F_ = 500, Table 2) was used for curve fitting, and the rest for model validation. In addition, signal intensity values that were to the left of *I_max_* and less than 80% of *I_max_* (e.g. the 0% formamide data point in the perfect match example of Figure 1A) were discarded to prevent the influence of possible kinetic factors at lower formamide concentrations. Thus, a total of 3,630 data points were used in the fitting with 500 probes. Using this data set we compared three models (M1–M3) with different degrees of complexity.

Initially, we evaluated the simplest model (M1, Table 2) where ΔG°_sln_ obtained from UNAFold was used to predict the binding free energy. Therefore, the only general parameters of fitting were *m* and {*P*}_o_. The best-fitting *m*-value showed 0.522 kcal/mol free energy increase with every percent of formamide added, while the effective probe concentration was equivalent to 0.0025 nM (Table 2). These parameters can be assumed to have converged to true values, as the average residual squares of 0.0207 units per data point agreed well with the average error squares in the validation set of 533 independent probes (*ε^2^_val_* = 0.021) (Table 2). The residual squares translated into a significantly larger *s^2^* value of 0.024 (Table 2), since this statistic is based on a degree of freedom (Equation 5) that takes into account all individual *γ* constants in addition to the two general fitting parameters and is therefore significantly smaller than the total number of formamide data points of 3,630 (i.e., *ν* = *n*–(500+2) = 3128). Thus, the *s^2^* value of the simple model was set as the reference point to test against the goodness of fit for the other models, in addition to the coefficient of determination (*R^2^*), which was 0.87 (Table 2).

Next, we evaluated a slightly modified model, designated M2 (Table 2), which included dangling end effects in free energy calculations. Dangling ends, including terminal mismatches, have been shown to contribute significantly to duplex stability with in-solution hybridizations [41], [42]. We therefore employed UNAFold to derive ΔG°_sln_ values with dangling parameters (ΔG°_sln,with dangling_). The calibrated and validated model showed a higher *s^2^* than M1 (Table 2), and therefore, we excluded dangling ends from our framework.

It has been shown that the establishment of specific nearest neighbor free energy rules for microarray hybridizations can improve predictive ability [33], [43]. We therefore developed model M3 for better predictions of perfect matching duplexes (Table 2). The ΔG° value (designated ΔG°_ma_) was calculated for every probe using the free energies of ten DNA/DNA nearest neighbors, which were derived as part of the general fitting parameter set in addition to *m* and {*P*}_o_. To get the total free energy of binding, a constant initiation free energy penalty (ΔG°_ini_ = 1.96 kcal/mol) was used rather than deriving it for microarrays. This was because ΔG°_ini_ and {*P*}_o_ were interdependent by the constant multiplication exp(−ΔG°_ini_/RT){*P*}_o_ because of the way free energy is summed and Equation 1 is constructed. Between the two variables we selected {*P*}_o_ to vary, since there was an in-solution based approximation available for ΔG°_ini_ [34]. The best-fitting parameters point to a constant value of 4.37·10^−4^ for the term exp(−ΔG°_ini_/RT){*P*}_o_, and ΔG°_ini_ and {*P*}_o_ values can be arbitrarily changed without affecting model fits as long as this constant is satisfied. The results with M3 (Table 2) showed significantly lower average residual squares (*Σr^2^*/*n* = 0.0083) than M1, as confirmed with the error squares of the validation set (*ε^2^_val_* = 0.0080). This was also reflected in an increase of the R^2^ value from 0.87 to 0.95 and a significant reduction of 0.0144 units in the *s^2^* statistic, which was more than twice the experimental variance calculated as 0.0061 based on the standard deviation of all data points (not shown), and therefore, the statistical difference between M1 and M3 was remarkable [44].

**Figure 3:**
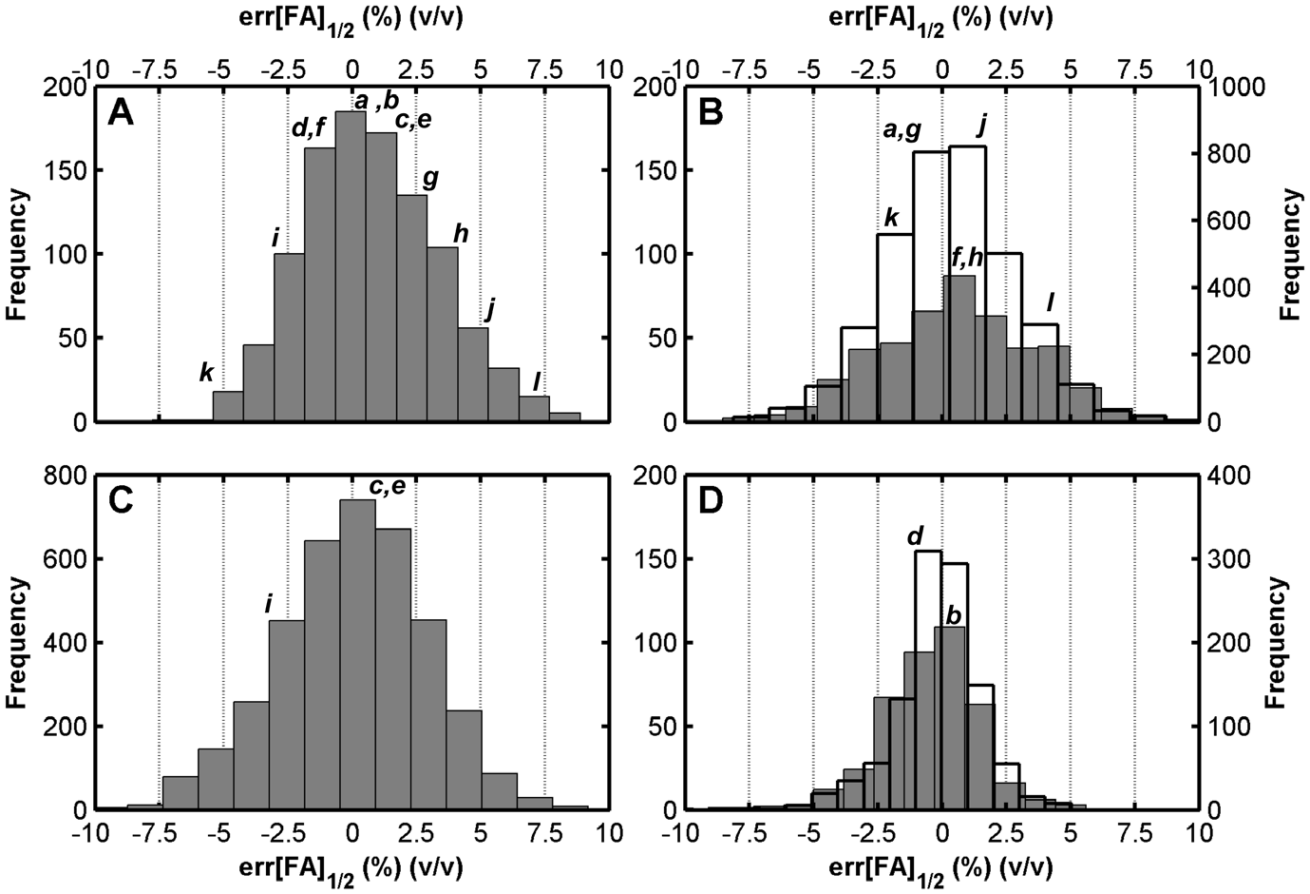
Histograms of prediction errors. (**A**) Perfect match (Length set), (**B**) central mismatch (OneM set; open bars, right axis) and bulged mismatch (Gap and Insertion sets; grey bars, left axis), (**C**) positional mismatch (PosM set), and (**D**) two-mismatch (TwoM set; open bars, right axis) and tandem-mismatch (Tandem set; grey bar, left axis) probes. Lower case letters indicate bins to which probes in corresponding panels of Figure 2 belong.

Example predictions with M3 are shown for 12 perfect match probes in Figure 2 (biased sampling) and 100 more in Figure S1A in Supporting Information (random sampling). The upper panels of Figure 2 show better fits than the lower ones. To evaluate the global fitting quality, we calculated the distance between theoretical and experimental profiles based on half-denaturation points (|err[FA]_1/2_|), as illustrated in Figure 1A. The theoretical half-denaturation point, [FA]_1/2,pred_, is defined the same as [FA]_1/2,exp_ (see above and Figure 1A), except that it is calculated for the continuous theoretical curve where the maximum value is always attained at 0% formamide. The resulting distribution of the predictive errors in formamide curve positioning is shown in Figure 3A. Most predictions were represented by the good fits in Figures 2A–F, as can be seen from respective labelings in Figure 3A. In fact, average absolute distance between theoretical and experimental profiles was 2.1±1.7% in formamide concentration, with more than 93 percent of probes having <5% distance (Table 2). These numbers also show significant advancement of predictive power over the solution-based M1 model (Table 2).

**Figure 4:**
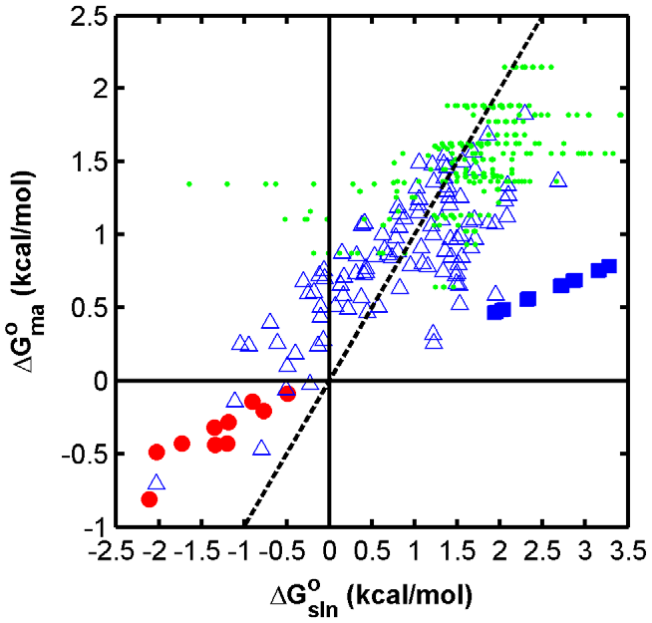
Relationship between in-solution and microarray free energy values (at 42°C). Red circles, nearest neighbors; blue triangles, single mismatch loops; blue squares, single bulged mismatch loops; green dots, tandem mismatch loops.

Best-fitting nearest neighbor free energies of M3 are presented in Table S1A, together with their in-solution matches and plotted in Figure 4. Although the scale of microarray parameters seems lower (about 1 kcal/mol reduction in magnitude of free energy) this was offset by a high effective probe concentration of ca. 0.010 M, compared to that when in-solution parameters were used (Table 2). The resulting *m*-value showed 0.173 kcal/mol decrease in the magnitude of free energy at every percentage of formamide, not very different from what was previously obtained for FISH (0.2–0.3 kcal/mol/%, [23]). Given the excellent correspondence with experimental profiles, the nearest neighbor parameters, the *m*-value, and the effective probe concentration obtained from M3 formed the backbone of our modeling framework for the evaluation of mismatches (Table 2).

### Central Single Matches

The destabilization effect of a single mismatch (ΔΔG°) is represented by a loss term and a gain term (Equation 2) due to the replacement of two nearest neighbors with an internal loop [33], [45]. The loss term can now be calculated based on the microarray nearest neighbor parameters from M3 (hence designated ΔG°_NN_−_,ma_). To establish the free energy rules for loop stability, we used a large set of probes named OneM (Table 1). Probes were created by inserting all three possible single mismatches in the central 11^th^ position of the perfect matches of the TileE set (Table 1) to avoid positional effects initially.

Based on the good correlation between in-solution and microarray nearest neighbor free energies previously obtained with M3 (Figure 4), we initially assumed that the loop free energy is a linear function of the in-solution values (i.e., ΔG°_MM Loop_ = αΔG°_SM,sln_+β, see M4 in Table 2). The calibrated and validated model M4 showed an *s^2^* value (0.0107) higher than the perfect match model M3, and yet showed a similar goodness of fit based on R^2^ and |err[FA]_1/2_| evaluations (Table 2). However, for best predictions, we developed M5 to derive specific free energy parameters for all individual loops (ΔG°_MM Loop_ = ΔG°_SM,ma_, Table 2). These loops are represented by 104 mismatch triplets that have all combinations of a middle mismatch and two flanking base pairs (Table S1B). Curve fitting was done separately for each triplet to find the best-fitting values of ΔG°_SM,ma_ as listed in Table S1B. Since the number of available probes was as low as 7 for some triplets (highest sampling size was 65), we included all probes in this analysis for the maximal use of the experimental data. The results showed that M5 outperformed M4 in terms of all goodness of fit criteria (Table 2).

The relationship between in-solution and microarray loop free energies was significantly scattered (Table S1B, Figure 4), hence the better fitting quality of M5 than M4. However, microarray and in-solution mismatch stabilities seemed to be on a similar scale unlike with nearest neighbor values (Figure 4). Example model fits with these values are shown in Figures 2A, 2G, 2J, 2K, and 2L and their representative ability is indicated in Figure 3B. In addition, Figure S1B presents profiles for 100 randomly selected probes. We see in Figure 2 both well-developed (2A) and truncated (2G and 2F) sigmoidal profiles with perfect fits, implicating the accurate identification of a large range of overall free energy values (ΔG°_ma_ = −2.5 to −5.2 kcal/mol). Although the best-fits were not validated by an independent subset in this case (i.e., N_F_ = N_T_, Table 2), other mismatch datasets were used for the verification of the choice of M5, as will be seen below.

### Positional Single Matches

**Figure 5:**
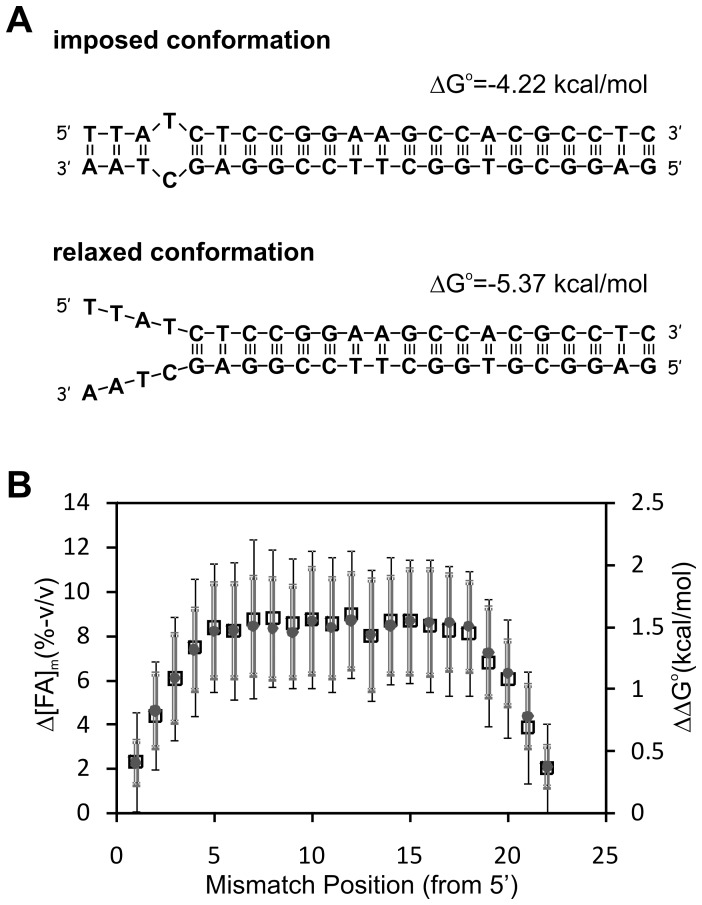
Effect of mismatch position on free energy and formamide denaturation. (**A**) Example probe (E844–865_4GT) with lower free energy at relaxed conformation as compared to imposed duplex with one mismatch. (**B**) Experimentally observed shift in the half-denaturation point (Δ[FA]_1/2,exp_ – open squares and black error bars) and calculated minimum free energy change (ΔΔG° - grey circles and error bars) upon insertion of a single mismatch, as a function of mismatch position. Values, averages; error bars, standard deviations.

The positional dependence of mismatch stability has been shown in multiple studies (e.g. [8], [43], [46], [47]) with a general agreement that mismatches towards the ends are less destabilizing than those in central positions. We addressed positional effects mechanistically, using the PosM set (Table 1) and the idea of relaxed ends illustrated in Figure 5A. In theory, a probe with a mismatch in a terminal position may have a more favorable (more negative) free energy in a relaxed conformation that leaves the bases spanning positions from the mismatch to the end unpaired. This happens when the free energy penalty of the loop (i.e., a positive ΔG°_MM Loop_ value) is larger in magnitude than the cumulative negative contribution of terminal base pairs clamping the duplex together, thereby causing an overall positive free energy contribution in the closed conformation. Therefore, the free energy of both imposed and relaxed conformations (Figure 5A) should be calculated and the most negative used. We adjusted our free energy calculations to include this effect for the positional dataset and compared the modified ΔΔG° term (i.e., the free energy difference from the perfect match duplex) with the experimental shift in the half-denaturation point (Δ[FA]_1/2,exp_) upon the insertion of the mismatch (i.e., the distance between the denaturation profiles of a mismatched probe and its perfect match version). The average Δ[FA]_1/2,exp_ shifts shown in Figure 5B revealed a very strong positional trend starting at the 4^th^ position from each terminal, which was almost perfectly captured by the modified free energy calculations.

We tested models M4 and M5 against the positional dataset without making additional calibrations. The results revealed better goodness of fit values for M5 in all terms, thus confirming that the derived microarray-specific mismatch parameters were more informative than in-solution parameters. The error squares with M5 were larger in the positional dataset compared to the central mismatch dataset (i.e., compare *s^2^* and *ε^2^_ov_* values in both sets), but the fitting quality was still satisfactory with a comparably high R^2^ value of 0.94 and more than 91 percent of the probes having less than 5% (v/v, formamide concentration) error in the prediction of half-denaturation points (Table 2 and Figure 3C). Example fits in Figure 2 show two terminal mismatches that are very difficult to discriminate from the perfect match (2C and 2E), as well as one with moderate discrimination potential (2I), which were captured by the M5 model. Figure S1C presents profiles of 100 randomly selected probes from this dataset. The relaxation adjustment adopted during the positional analysis was consistently implemented in the models presented below.

### Bulged Mismatches

A bulged mismatch occurs when there is an insertion or deletion in an otherwise conserved target site and can potentially have a comparable stability to an average single mismatch [48], [49]. We combined the Gap (deletions) and Insertion (insertions) probe sets (Table 1) to develop free energy rules for bulged mismatches. The strategy was the same as with single mismatches, except that deletions removed two nearest neighbors and insertions only one nearest neighbor for the calculation of the loss term in Equation 2, which still required existing nearest neighbor values from M3 (ΔG°_NN_−_,ma_). Thus, modeling aimed at the derivation of the missing loop terms for bulged mismatches (ΔG°_BM,ma_), which were represented by 64 triplets in total, including all combinations of a bulged mismatch and two flanking base pairs (see Table S1C).

The general screening procedure yielded 467 probes for testing. Although this set covered all mismatch triplets, there was not sufficient information for deriving specific free energy values for each loop (2 to 14 probes per loop). Thus, we only tested models assuming a linear relationship between in-solution loop parameters and microarray parameters (i.e., ΔG°_BM,ma_ = αΔG°_BM,sln_+β). Results with and without the constant term of the linear relationship (β) revealed that it did not contribute to the overall fitting quality (i.e., *s^2^* values were the same for M6 and M7, Table 2). We therefore selected M7 as the preferred method, which showed goodness of fit parameters similar to single mismatch models (Table 2). The relationship of loop free energies to original in-solution parameters is depicted in Figure 4, with a line of data points of slope α (0.238). As an interesting result, the plot suggests that bulged mismatches are similar to moderate single mismatches in microarray-based stability, in contrast with in-solution parameters where bulged mismatches are generally more destabilizing. Example fits are illustrated in Figures 2F and 2H, both of which show poor mismatch discrimination potential due to stable bulged mismatches. Figure S1D presents 100 additional randomly selected probes. The distribution of predictive errors at half-denaturation points was similar to other mismatch datasets (Figure 3B).

### Two Mismatches

In principle, the stability of two separate single mismatches can be calculated by adding their respective ΔΔG° values. Thus, we tested M4 and M5 developed for single mismatches, using a set of two separate mismatches (TwoM, Table 2). Once again, error square parameters in Table 2 showed that microarray-specific free energy rules (M5) were better predictors than the linear mapping from in-solution values (M4), although they also showed lowered fitting quality in comparison to single mismatches. The predictive errors were least of all according to the distribution in Figure 3D, but this was biased by the fact that complete denaturation happened at very low formamide concentrations (0–10%) in general with double mismatches. An example is provided in Figure 2D, while 100 more randomly selected probes are presented in Figure S1E.

### Tandem Mismatches

A special type of double-mismatch is the tandem mismatch, which involves two adjacent mismatches [50]. Thus, the loss term in Equation 2 should include three nearest neighbors and the loop term a quadruplet that accommodates a tandem mismatch pair in the middle flanked by two base pairs. In the simplest case (model M8, Table 2), we again assumed a linear relationship of the loop term (ΔG°_TM,ma_) with in-solution parameters (ΔG°_TM,sln_) obtained from UNAFold. However, the massive number of 1,176 combinations of tandem quadruplets lowers the confidence in the indirect calculation of in-solution parameters based on limited data [42]. Thus, simple microarray-specific rules may again be preferred to solution-based modeling.

We developed M9 with a set of eight rules (see Table S1D) describing the stability of tandem mismatches based on our observations with single mismatches (Table S1B). The model first divides tandem mismatch quadruplet into two halves, each having a closing base pair and a mismatch. Based on single mismatch data, whether the closing pair is an AT- or GC-type affects the loop stability, such that GC pairing generally stabilizes the mismatch. As for the mismatch type, GG, GA, and GT mismatches show significantly higher stability than others. Therefore, our eight rules (Table S1D) are established to give a different score to each one of the 8 combinations of closing pair (two types) and mismatches (four types). The two scores from either half of the quadruplet are then added to obtain the overall free energy of the loop (ΔG°_TM,ma_). Initial results with 300 probes used for model calibration yielded a better *s^2^* statistic than the solution-based M8 model. However, the validation set showed somewhat different error squares than residual squares indicating there was benefit of using more probes (Table 2). We therefore obtained final best-fitting scores (Table S1D) using the entire probe set for curve fitting (Table 2). The free energies of the quadruplets in our dataset were related to in-solution values in a way similar to single mismatches (Figure 4), except for some combinations of GT and GG base pairs that were predicted by UNAFold to stabilize the loop by a significantly negative free energy, but according to our parameters did not show a negative contribution (Table S1D). The error distribution of this model (Table 2 and Figure 3D) was similar to separate double mismatches. An example fit is provided in Figure 2B, while 100 more randomly selected probes are presented in Figure S1F.

### The Effect of Target Concentration

**Figure 6:**
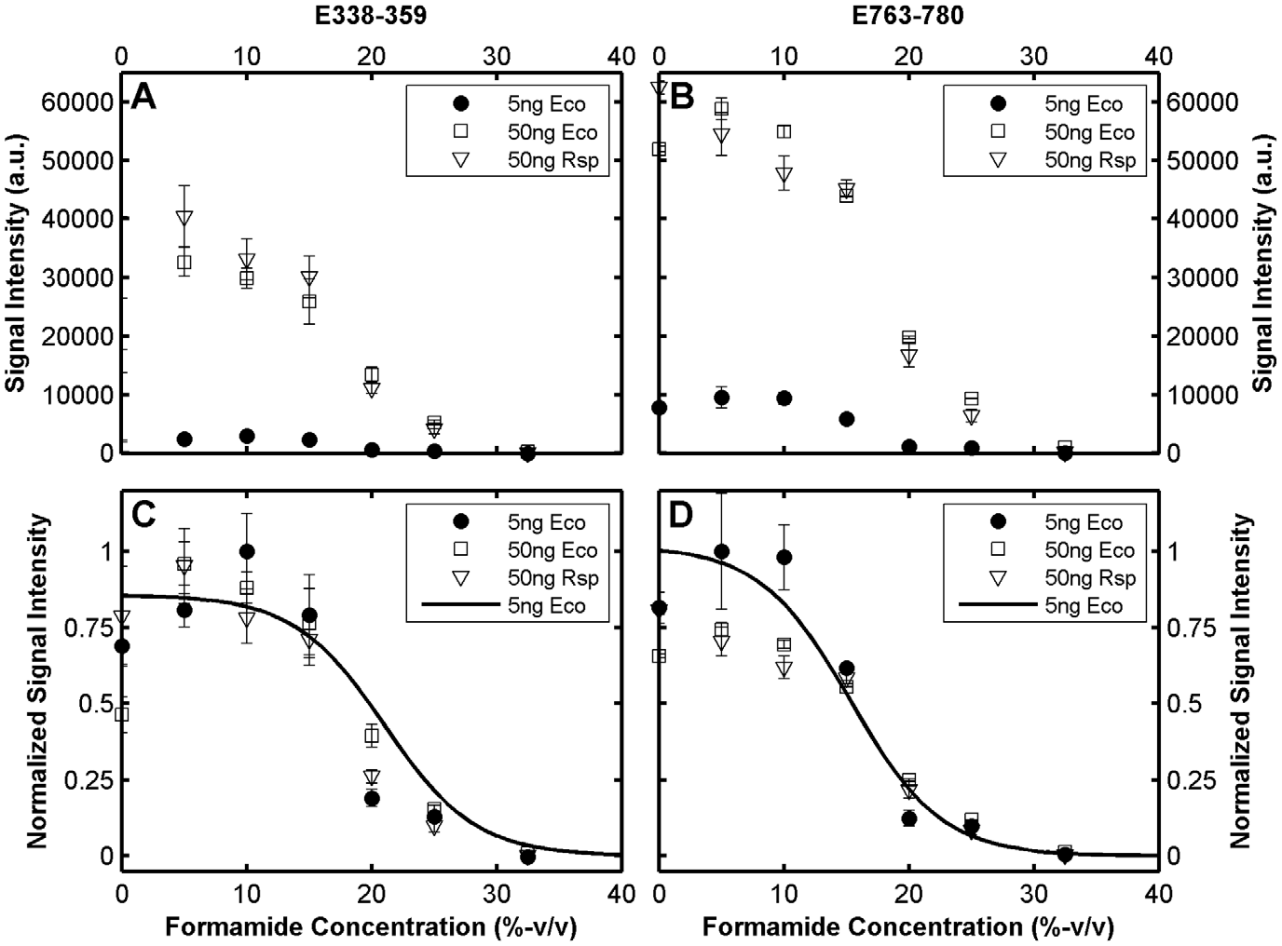
Effect of target concentration and type of target molecule on formamide denaturation profiles. Probes E338–359 (**A** and **C**) and E763–780 (**B** and **D**) are shown. Both probes perfectly match the 16S rRNA gene of both *E. coli* (Eco) and *R. sphaeroides* (Rsp).

In this study, a uniform amount of target (5 ng in a hybridization buffer of 8 µL) was used during the model development. Although total DNA concentration can be controlled in environmental applications, relative abundances of organisms in the analyzed sample can cause a large range of target and non-target concentrations. Therefore, it is important to know if target concentration affects formamide curves in a way that undermines model predictions. To test this effect, we used an order of magnitude greater concentration of our target (50 ng) in independent hybridizations with the same formamide series. Furthermore, we analyzed an additional dataset obtained with 50 ng of the 16S rRNA gene of *R. sphaeroides*. As exemplified in Figure 6A with a probe perfectly matching both targets, when the signal did not reach saturation levels in the signal scale, 50 ng target yielded fluorescence values consistent with the 10X increase in concentration. Despite the remarkable gap in fluorescence levels, the profiles aligned well when normalized and the theoretical prediction was not significantly affected (Figure 6C).

When concentrated targets were used, the experimental profiles of most probes were affected by signal saturation, which was evident at fluorescence levels of about 40,000 units and above (see Figure S2). Importantly, this was not the case with the modeling datasets, since the highest probe signal intensity encountered was less than 40,000. Typical profiles affected by saturation are shown in Figure 6B, with another probe that targets both *E. coli* and *R. sphaeroides*. While the level of maximum signal was about 10,000 units with the 5 ng target, the 10X increase in concentration could not carry this beyond a level of around 60,000 units, implicating that the full sigmoidal profile could not be observed. Accordingly, normalized curves could be matched only at higher formamide concentrations provided that the normalization was adjusted to offset the signal saturation effect (Figure 6D).

The data in Figure 6 represent the general case except for small deviations that can be explained by experiment-to-experiment variability. It follows from the agreement of experimental profiles that the predictive ability of our models should not be significantly affected by even large concentration changes. Indeed, a total of 181 *E. coli* probes (perfect matches) not affected by saturation (i.e. *I_max_*<40,000 a.u.) showed |err[FA]_1/2_| values of 1.89±1.33% when hybridized with the 50 ng *E. coli* target. The same analysis applied to 50 such *R. sphaeroides* probes (TileR set, Table 1) resulted in an absolute error of 1.82±1.22% with the 50 ng *R. sphaeroides* target. These numbers agree well with the data in Table 2. We therefore conclude that our models should be applicable to environmental samples with a range of concentrations, as long as signal supersaturation is prevented by the optimization of total target concentration.

## Discussion

We adopted the idea of formamide denaturation from FISH protocols, where the strategy is successfully used for balancing probe sensitivity and specificity [3], [20]. In FISH, the optimization is generally carried out experimentally by establishing probe denaturation profiles (similar to those in Figure 1A) with pure cultures or clones of target and non-target organisms, an option that is clearly not feasible for high density microarrays given the large number of probes. Therefore, we did not only show the proof of principle for formamide denaturation in microarrays, but also developed mathematical models for predicting the melting profiles of perfect and mismatched probe/target pairs. The predictive accuracy for the position of the melting curves is generally within 5% formamide of the half-denaturation point, remarkably better than what was previously achieved for FISH with a multi-state LFEM [23]. This must be in part due to the absence of a stable secondary structure in the fragmented DNA target of the microarray method studied, in comparison to the full length rRNA target in FISH. However, the derived nearest neighbor free energies in our current two-state model may be reflecting an averaged out partitioning of nearest neighbors between different states, including secondary interactions within or between target fragments. This is consistent with the fact that the scale of nearest neighbor parameters turned out to be lower in microarrays than in solution, while that of mismatch loops were consistent (Figure 4). Overall, we believe that the predictive power achieved by the two-state LFEM in this study can significantly improve probe design and optimization in microbial ecology applications of oligonucleotide microarrays, as will be discussed below.

The curve-fitting procedure used in this study was carefully devised to include probes with a large range of melting points. A key aspect of the mathematical framework was the use of *γ* factors to adjust theoretical curves when a full sigmoidal profile was not obtained (Equation 3). We graphically explain how the *γ* factor affects curve-fitting, and compare alternative approaches to estimate these factors in Figure S3. For nearly full sigmoidal profiles, lower formamide concentrations represent points of approximately 100% hybridization efficiency. In these cases, it is sufficient to match experimental profiles normalized by *I_max_* to theoretical profiles with *γ* = 1. On the other hand, when a probe melts at low formamide concentrations, the sigmoidal curve is truncated and the experimental hybridization efficiencies cannot be determined with high enough confidence. Hence, an adjustment of the theoretical curve using *γ*≠1 provides a better match of theoretical and experimental profiles. We tested three different approaches to calculate *γ* factors. In our preferred approach, *γ* factors were included as best-fitting parameters. This did not affect the modeling of formamide denaturation since the loss of the degrees of freedom by best-fitting *γ* values was taken into account in key statistics (Equation 5) and *γ* factors do not mathematically change the melting point, which our modeling effort aims to predict. Nonetheless, the use of one *γ* factor per probe may seem to have caused overparameterization during model development. We did additional statistical tests to independently show that this is not the case (Table 3). The details of these analyses are explained in Text S1. In one set of tests, the selected models were calibrated with *γ* = 1 for all probes. In another set, we set *γ* equal to the inverse of the maximum predicted hybridization efficiency (i.e., efficiency at 0% formamide) so that the truncated denaturation profile always started at a value of 1, consistent with the normalization of experimental intensities with *I_max_*. On the overall, our evaluations demonstrate that the model predictions were driven by thermodynamic parameters. Although the alternative methods resulted in similar conclusions for the test models (Text S1 and Table 3), a unique advantage of using best-fitted *γ* factors is the effective buffering of experimental artifacts such as the increase in signal intensity at low formamide concentrations (Figure S3C) and general experimental noise. Thus, with the help of *γ* factors, we were able to estimate a series of thermodynamic parameters, with minimal influence of experimental artifacts, for the prediction of probe denaturation in a large range of melting points.

**Table 3:** Additional statistical testsa,b.

		Parameters				Statistics					|err[FA]_1/2_|	
Test	Model	eliminated	permuted	randomized	fitted	*Σr^2^/n*	*s^2^*	*ε^2^_val_*	*ε^2^_ov_*	*R^2^*	*µ*	<5%
T1	M3	γ-factors	na	na	na	na	na	na	0.0118	0.93	2.1	93.4
T2	M3	na	γ -factors	na	na	na	na	na	0.0158	0.90	2.1	93.4
T3	M3	na	*ΔG*°*_NN_*	na	na	na	na	na	0.035	0.78	5.7	52.5
T4	M3	na	*ΔG*°*_NN_, Probe* c	na	na	na	na	na	0.052	0.68	7.7	38.5
T5	M3	na	Na	*P_o_, m*	na	na	na	na	0.19	−0.18	14	10
T6	M1	γ -factors	Na	na	Po, m	0.0321	0.0322	0.0319	0.0320	0.80	2.7^d^	86.0^d^
T7	M3	γ -factors	Na	na	*Po, m, ΔG*°*_NN_*	0.0118	0.0119	0.0115	0.0116	0.93	2.1	93.8^d^
T8	M5	γ -factors	Na	na	na	na	na	na	0.0147	0.90	2.0	94.5
T9	M5	na	γ -factors	na	na	na	na	na	0.028	0.815	2.0	94.5
T10	M5	γ -factors^e^	Na	na	na	na	na	na	0.0107	0.93	2.0	94.5
T11	M5	na	*ΔG*°*_MM Loop_*	na	na	na	na	na	0.0144	0.905	3.0	82
T12	M5	γ -factors^e^	Na	na	*ΔG°_MM Loop_*	0.0104	0.0107	0.0104	0.0104	0.93	2.0	94.4

a See Table 2 for the definition of models and parameters and the reference values. See Text S1 for the details of the statistical tests.

b Randomization and permutation tests are based on at least 100 runs until convergence. Significant figures in these results reflect the uncertainty in the converged values.

c Probes were permuted while maintaining the original sequence of each probe. This test corresponds to permuting the probe length in addition to the nearest neighbor free energies.

d Show improvement over original models although statistical parameters indicate otherwise. The discrepancy reflects the fact that half-denaturation point is not a perfect representation of the melting point for experimental profiles without a plateau (e.g., see Figure S3C). This adversely affects the results with *γ*-factors more than without, as the models without *γ*-factors tend to compensate for the lack of good fitting in the vertical by moving closer to experimental values in the horizontal, although this movement does not mean a better match. Since the original models in the main text always use *γ*-factors, the evaluation of model predictions are conservative and more accurate with respect to half-denaturation points. This analysis provides just another way of seeing how *γ*-factors buffer experimental artifacts as discussed in Text S1.

e Best-fitted *γ* replaced by a model-derived factor (see Text S1).

Certainly, our model does not capture free energy parameters for all possible mismatch conformations in a probe/non-target duplex (e.g., bulged mismatches with two deletions or inserts, three adjacent mismatches, etc.) to directly predict their effect on hybridization efficiency. But the most important (stable) ones were systematically covered, which allowed us to extend the predictive algorithm to other (complex) mismatch conformations by penalizing them with conservative parameters (see Text S2; Table S2 shows the list of extended free energy rules). The extended model can be used in the calculation of the hybridization efficiency of most duplexes with reasonable confidence (see Text S2 and Figure S4D). The algorithm that simulates formamide denaturation with LFEM using all thermodynamic parameters established in this study (Table S1 and Table S2) is named “ProbeMelt” and made freely available both as an on-line web tool at http://DECIPHER.cee.wisc.edu and a package in R programming language (R Foundation for statistic computing, Vienna, VA) (see Text S2 for details).

### Differences with Previous Approaches

The governing equation of our mathematical framework (Equation 1) is similar to Langmuir isotherms commonly used for describing the relationship between target concentration and the fraction of target-bound probes [33], [51], [52], [17]. In addition to lacking a denaturation term (i.e., m-value and formamide concentration), Langmuir models differ from LFEM with the assumption that the target is in excess of probe. This assumes probes are saturated at a hybridization efficiency of 1, which was clearly not the case in our experiments as the fluorescence intensity at the plateaus of sigmoidal melting profiles (i.e., points of hybridization efficiency ∼ 1) largely varied and was consistently elevated by increased target (Figure 6). As shown in Figure 6B, we encountered signal saturation with the highly concentrated target, but this is likely due to the sensitivity of the scanner as the signal always converged to the upper limit of the measurable signal scale (Figure S2). At lower signal values, 10 times more target caused around 10 times higher signal intensity (Figure 6A and Figure S2). Thus, the data was more consistent with the depletion of the target rather than the probe, as was assumed in the derivation of Equation 1. When the Langmuir model is rejected, the competition for the limited target molecules may need to be addressed [53]. However, this competition effect would be evidenced by a good correlation between predicted hybridization free energy and fluorescence intensity, which also was not the case in our study (Figure 1B). The actual mechanism of surface hybridizations on microarrays is not well understood [18], [28], [54]. It is beyond the scope of this study to justify the conceptual model behind our mathematical framework, except to show that the simulations with the equilibrium model adequately represented experimental denaturation profiles, thereby fulfilling our main goal.

**Figure 7:**
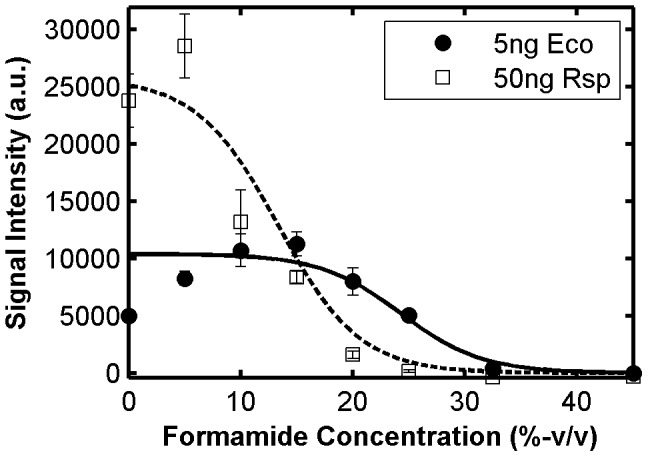
Formamide denaturation profiles with conventional target and highly abundant non-target. The example probe, E751–772, is a perfect match to the 16S rRNA gene of *E. coli* and has one mismatch to *R. sphaeroides*. Curves are theoretical predictions fitted to the experimental scale. Eco, *E. coli*; Rsp, *R. sphaeroides*.

Unlike most other models of oligonucleotide microarray hybridization [51], [53], the aim of the LFEM is not to find the concentration of the target molecules, but to predict the hybridization efficiency at a given formamide concentration, which produces the normalized melting profiles regardless of the concentration. Focusing the predictive power on target concentration is problematic for diagnostic applications in several ways. First of all, since most DNA-targeted protocols are end point PCR-dependent, the concentration in question is a biased quantity even if accurately predicted [55]. Secondly, concentration predictors work on the signal intensity variation assuming it is a function of relative target concentration as well as binding free energy. However, if the target is labeled by the common random priming method as in this study, there will be significant differences in signal intensity over different fragments within the same target (e.g., the fluctuations in Figure 1D), which clearly undermines the ability to pick target to target differences. But most important for microbial ecology applications, a strong signal response that leads to the prediction of a concentration may be both from a target or a closely related non-target as depicted in Figure 7 (e.g., at 0% formamide). Thus, detecting the absence/presence of organisms at high stringency (e.g., 20–25% formamide, Figure 7), rather than measuring their concentration, seems to be a more feasible approach, which requires the calculation of melting curves.

Another property of hybridization that our LFEM is not trained to capture is the decrease in signal intensity upon the insertion of a mismatch (e.g., the lowered plateaus of mismatches in Figures 2A and 2H). Since the theoretical hybridization efficiency is close to 1 in the plateau of both perfect and mismatched duplexes, the LFEM cannot directly address this issue, although the model is unaffected by it because of the normalization by *I_max_*. Arguably, mismatch stability (i.e., ΔΔG°) can be quantified by the decrease in signal intensity level as another way of developing free energy rules for mismatch discrimination, as was done previously [33]. However, the prediction of the change in plateau levels is also not a viable approach for applications in microbial ecology, because the moderate decrease in signal associated with a mismatch can be easily offset by the relative abundance of the non-target organism. Figure 7 illustrates this phenomenon as well. Once again, a more realistic approach is to force the mismatched duplex to dissociate by applying stringent conditions, so that it counts as absent even when it is highly abundant (e.g., Figure 7, 20–25% formamide).

To the best of our knowledge, the only other systematic use of denaturation trends for microarray optimization appears in the non-equilibrium thermal dissociation (NTD) approach [10], [46]. Applications of NTD involve the derivation of dissociation profiles with reference organisms as well as environmental samples for matching the two [9], [56], [57]. This approach is not feasible with high-density microarrays designed to target thousands of organisms at once [13], [15], [16]. In addition to the use of formamide rather than temperature for denaturation, an important difference that sets apart our methodology from NTD is the adjustment of stringency during the long hybridization period to achieve equilibrium-like conditions, whereas NTD is based on a kinetically-driven dissociation during the wash step [58]. Thus, we took advantage of equilibrium thermodynamics and developed predictive algorithms to create a feasible alternative to the experimental testing of probes for optimization. Furthermore, we do not recommend the matching of predicted melting profiles to experimental ones, as not only would this require an even higher accuracy of predictions than what we have obtained, but also the possible superimposition of signals from perfectly matching and mismatched targets could undermine the curve-matching approach [58]. The recommended use of our modeling approach for the rationalization of probe design and optimization is described next.

### Application of LFEM to Diagnostic Probe Design

In this section, we describe how the LFEM-based calculations of hybridization efficiency can be useful for the optimization of probe sensitivity and specificity in microbial ecology applications. The general practice aims at determining the absence/presence of organisms by setting a signal intensity threshold to define successful target capture [13], [19], [59], [60]. When there is sufficient signal from the capture of a mismatched non-target gene, probe specificity is compromised. To minimize the chance of false positive identification because of cross hybridizations, multiple probes with identical or nested coverage are designed per target group (i.e., operational taxonomic unit; OTU), and nearly all of these are demanded to be bright in order to call a target group as present (e.g. 9 out of 10 probes). This assumes the probes are designed with high enough sensitivity to avoid signal intensities below the threshold when the perfect match target is captured (e.g., if 2 out of 10 probes targeting an existing OTU fail to give bright signal, then it is a false negative identification). Therefore, the obvious target for our predictive denaturation approach is the design of optimal probes and hybridization conditions to obtain the highest possible hybridization efficiency with the targets while keeping the hybridization efficiency with the non-targets at the lowest possible level, thus minimizing the chance of false positive and false negative identification of OTUs.

For the demonstration of optimization, we did sensitivity and specificity analysis with the 16S rRNA gene of two organisms, *E. coli* and *R. sphaeroides*, such that *E. coli* served as target for perfect-match *E. coli* probes (TileE and Length sets in Table 1) and non-target for *R. sphaeroides* probes (TileR, Table 1), and vice versa. It is important to note that probes that would be filtered due to poor signal intensity during model development (see Methods) were included in these tests to avoid biasing the results. The amount of *E. coli* target was 5 ng and it represented an organism of moderate abundance assuming total DNA used in an environmental application was 50–100 ng. This number is consistent with our signal-optimized applications with real mixed communities where 70 ng of total target is used for hybridization without causing frequent signal saturation problems (not shown). The amount of *R. sphaeroides* target was 50 ng, and therefore, it represented an unlikely abundance of a single organism in total DNA causing signal saturation with most probes and challenging specificity at the extreme levels. The optimization of probe sensitivity and specificity by predictive modeling follows two steps.

**Figure 8:**
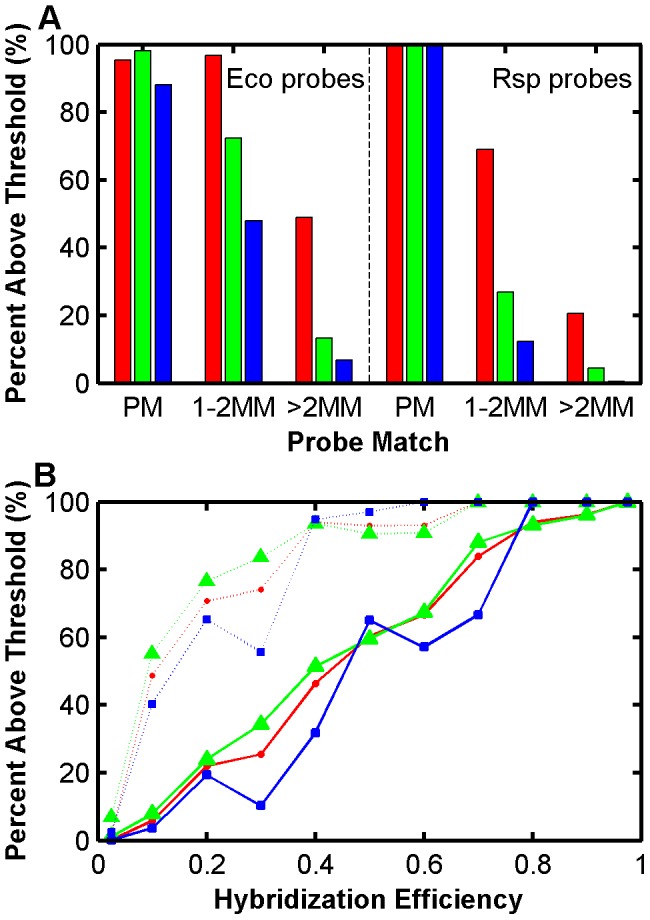
Fraction of probes above an arbitrarily defined threshold of 1750 fluorescence units. (**A**) Probes designed to have a melting point of 18–22% formamide and hybridized at 0% (red), 15% (green), and 20% (blue) formamide. Left panel, *E. coli* probes hybridized with 5 ng *E. coli* target (PM data) and 50 ng *R. sphaeroides* non-target (MM data); right panel, *R. sphaeroides* probes hybridized with 50 ng *R. sphaeroides* target (PM) and 5 ng *E. coli* non-target (MM). (**B**) The predictive power of hybridization efficiency for *E. coli* probes hybridized with 50 ng *R. sphaeroides* (dashed lines) and *R. sphareoides* probes hybridized with 5 ng *E.coli* (solid lines) for all mismatches (red), 1–2 mismatches (green), and 3–5 mismatches (blue). Data from formamide concentrations 10, 15, 20, and 25% were combined to maximize the sample space for each data point. x-axis shows midpoints of bins with a hybridization efficiency window of 0.1, except for end bins (window of 0.05).

First, since microarray hybridizations are typically performed at a single level of stringency, it is important to be able to design probes with similar formamide-based stabilities (i.e., similar melting points), to achieve a consistent level of hybridization efficiency with target organisms over thousands of probes. We can do this with the ProbeMelt algorithm by predicting melting points. In Figure 8A, we show the mismatch discrimination potential for probes designed to have a narrow range of predicted melting points, between 18–22% formamide. In the *E. coli* set there are 561 such probes that have one or more mismatches to *R. sphaeroides*. We tested the mismatch discrimination ability of these probes against this extremely abundant non-target. The results show that, when formamide is not present, the discrimination for 1–2 mismatches is not possible at all and about half of the probes with >2 mismatches give a bright signal. The situation changes radically at 20% formamide (Figure 8A), which represents the targeted melting point in the design of these probes. However, there is about a 12% chance of poor target capture at this high level of stringency (i.e., the corresponding perfect match column in Figure 8A shows only 88% above signal threshold) implying that sensitivity is not optimal. As a compromise, hybridization can be done at 15% formamide (i.e., ∼5% less than the predicted melting points), to decrease the rate of low signal from target to <2% and bring the rate of high signal from non-targets to about 72% for 1–2 mismatches and 13% for more mismatches (Figure 8A). Although mismatch discrimination potential seems low for 1–2 mismatches, it should be considered within the context of a multiple-probe strategy, which results in a false positive identification only when several non-target OTUs are captured by different probes. Since it is unlikely to have many such non-targets in the same environmental sample (i.e., total DNA is 50–100 ng while the tested non-target was 50 ng), these results show that the predictive formamide denaturation strategy can be useful to avoid false positive identification of even extremely abundant non-targets. The second half of Figure 8A shows the experimental simulation with 378 *R. sphaeroides* probes tested against 5 ng *E. coli* as the moderately abundant non-target. In this more likely scenario, using 15% formamide is enough to effectively suppress the signal intensity of mismatched probes (Figure 8A). Thus, 15% formamide can be an optimal point for the sensitivity and specificity of probes designed with a predicted melting point around 20% formamide.

It is clear from the experimental simulations in Figure 8A that not all mismatches can be perfectly discriminated even under optimal conditions, as could be anticipated by the proximity of some denaturation profiles encountered (e.g., Figures 2E and 2H). Therefore, an important question is whether problematic mismatches can be predicted beforehand. This brings us to the second step in optimization: the prediction of worst non-targets based on hybridization efficiency calculations during the design process. We show in Figure 8B the relationship between predicted hybridization efficiency and percent above threshold for all mismatched duplexes at four formamide concentrations. Consistent with our goals, the predicted efficiency of *R. sphaeroides* probes hybridizing with the moderately abundant *E. coli* non-target dictates the frequency of false signal. An important result here is that more than 2 mismatches can also bind effectively, as captured by the thermodynamic model. On the other hand, the extremely abundant non-target *R. sphaeroides* causes specificity problems with *E. coli*-targeted probes starting at ∼0.1 hybridization efficiency (Figure 8B). Nonetheless, probes predicted to have <0.05 hybridization efficiency, which are the majority of the population at all formamide points considered (e.g. 72% of the probes for 15% formamide), are still dim even when they have 1 or 2 mismatches. Thus, by defining probe specificity based on the hybridization efficiency with potential non-targets, probes with the best specificity scores can be selected during probe design with the help of the ProbeMelt algorithm.

In summary, we recommend the following steps for the systematic optimization of microarray protocols with LFEM: (i) prescribe a single formamide concentration for hybridization (e.g., 15% formamide), (ii) design multiple probes per target group (e.g., 10) to allow predictive errors without compromising identification, (iii) at every target site, adjust probe length to obtain a uniform probe stability throughout the array, such that the theoretical melting points are always slightly higher than the prescribed experimental formamide concentration (e.g., keep the probe melting points in the range of 18–22% formamide), (iv) set a specificity score for each probe candidate by calculating the hybridization efficiency with mismatched non-targets and select for probes that have best specificity scores. Steps *iii* and *iv* are applicable for designs with large target datasets since the ProbeMelt algorithm can evaluate more than a million probes per second. Step *iv* is also a significant departure from traditional design approaches based on mismatch numbers or types [61], [62], since it takes advantage of the thermodynamic parameter sets that were rigorously developed in this study. In addition to helping with the design and optimization phase, we expect our models to be useful for the interpretation of signal patterns from hybridized microarrays. Advanced algorithms for organism detection from complex array data have been developed [63], [64], [65], but they either lack predictive tools for the evaluation of probe hybridization with non-targets [64], [65], or use in-solution free energy parameters as a preliminary approach [63]. Therefore, it is not hard to imagine hybridization efficiency predictions improving the accuracy of interpretation algorithms for diagnostic microarrays.

### Application to other Platforms

Because of platform- and protocol-specific variables such as probe density and fragment length, our model should be applied to other types of microarrays with care. Obviously, the modeling parameters are optimized for 4-Plex Nimblegen arrays hybridized at 42°C, and therefore, the ProbeMelt algorithm developed in this study should be directly applied only for these conditions. While we do not expect the free energy rules to differ significantly in other similar platforms (if the temperature and hybridization buffers are not changed), it is anticipated that the effective probe concentration ({P}_o_) may need to be re-optimized when probe concentration or configuration are altered. On the other hand, more significant adjustments may be necessary if the target labeling procedure is different. For instance, if long, unfragmented target nucleic acids are prepared [66], significant competition with stable secondary structures may change the thermodynamics of binding. Thus, re-optimization may need to be extended to nearest neighbor rules or the *m*-value. In any case, probe sets similar to those used in this study can be included in a custom array design, so that the parameters can be re-optimized if necessary, following our modeling approach. Also, since our modeling framework is derived assuming the probes are not depleted by the local target the validity of this assumption needs to be verified as probe saturation has been clearly shown in some studies with other platforms [52]. If probe depletion appears to be the case, the hybridization efficiency term of this study can be redefined based on the ratio of target-bound probes as in Langmuir models [51], [52] and the same linear free energy approach can be applied. With the current technology of microarray fabrication allowing the placement of millions of probes on a slide, a set of ∼15,000 probes allocated for modeling can be a negligible amount. Extension of the specific formamide denaturation LFEM to other platforms could also be informative about the general applicability of the modeling framework, thereby helping with the efforts to understand the mechanisms of hybridization.

In conclusion, the thermodynamic modeling framework established to simulate formamide denaturation can be effectively used for the design and optimization of probes in microbial ecology analyses. For similar platforms and protocols obeying the assumptions of this work, the LFEM can be directly applied using the online ProbeMelt algorithm. For others, the systematic approach developed can be followed to customize the thermodynamic parameters.

## Supporting Information

Figure S1 **Figures of additional experimental and theoretical formamide melting profiles.**
(PDF)

Figure S2 **Signal saturation with highly concentrated target.**
(PDF)

Figure S3 **Curve fitting with different methods to align theoretical curves with normalized experimental profiles.**
(PDF)

Figure S4 **Figures for Text S2.**
(PDF)

Table S1 **Tables of free energy parameters describing duplex stability.**
(XLSX)

Table S2 **Extended free energy rules for quadruplets of nucleotide pairs in a DNA duplex.**
(PDF)

Text S1 **Best-fitted γ Factors and Additional Statistical Tests.**
(PDF)

Text S2 **Free energy calculations with nucleotide quadruplets.**
(PDF)
