# Supplementary material for: Modeling Formamide Denaturation of Probe-Target Hybrids for Improved Microarray Probe Design in Microbial Diagnostics
Source: PLoS One. 2012 Aug 27;7(8):e43862. doi: 10.1371/journal.pone.0043862 (PMC3428302; doi:10.1371/journal.pone.0043862)

Figure S1A. Experimental (circles) and theoretical (curves) formamide dissociation profiles of 100 randomly selected probes from Length set (Table 2). The labels (upper right corner of each graph) indicate probe name (top), I_{max} (bottom, first number), err$[FA]_{1/2}$ value (bottom, second number). See main manuscript for the descriptions of probe names and I_{max} and err$[FA]_{1/2}$ values. x-axis, formamide concentration; y-axis, normalized signal intensity; error bars, standard deviations.

Probes 1-8:

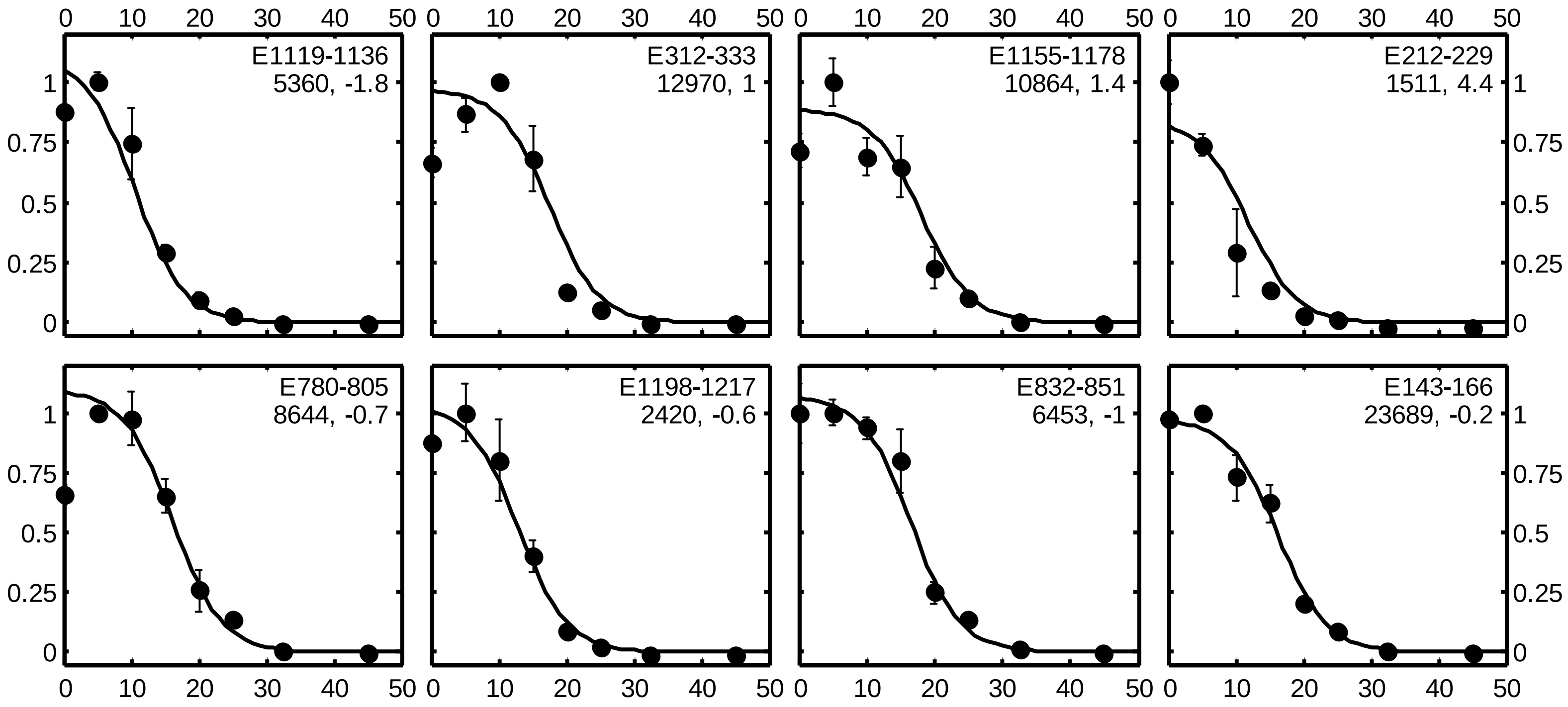

Figure S1A

Probes 9-20:

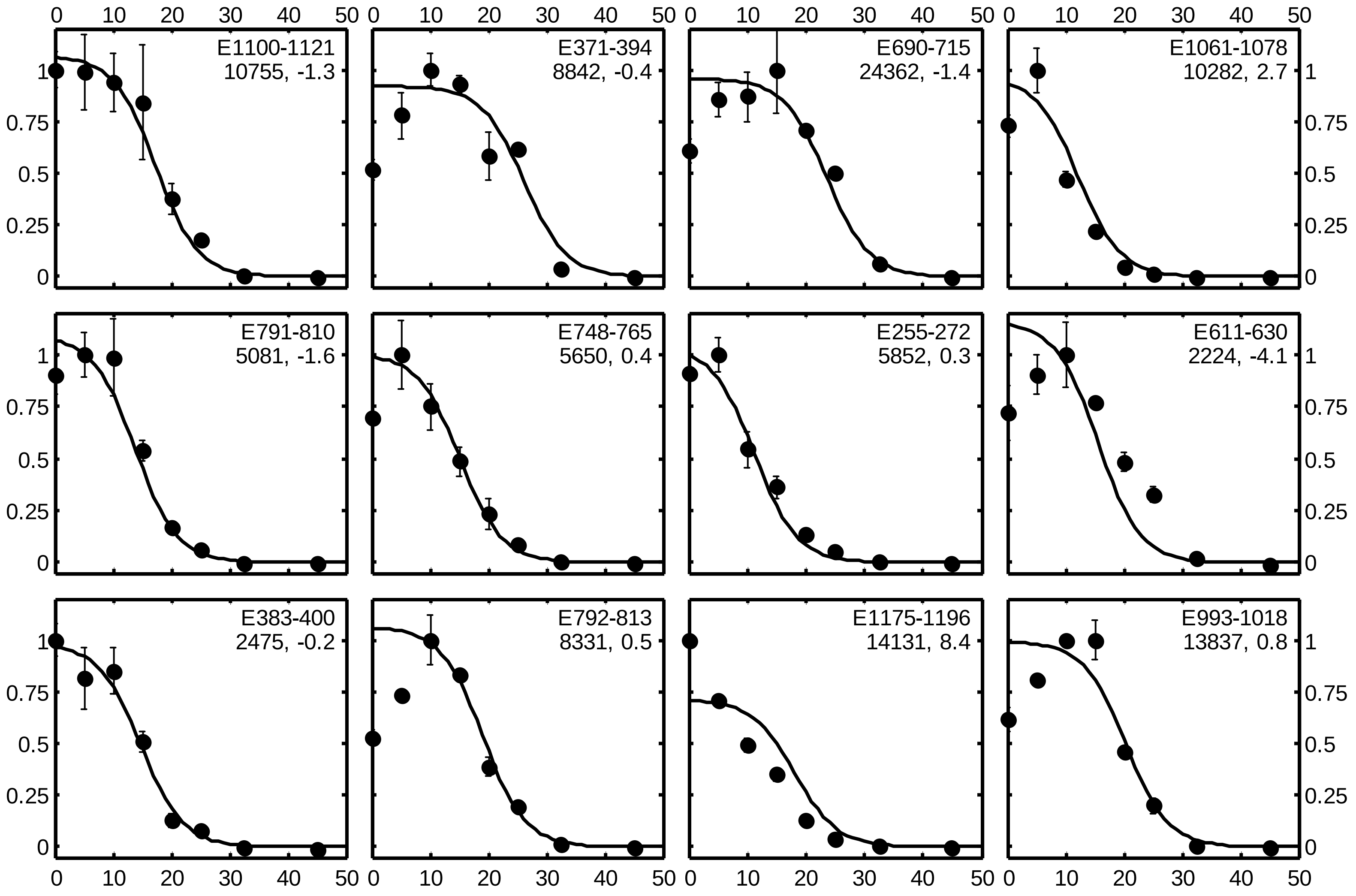

Figure S1A

Probes 21-32:

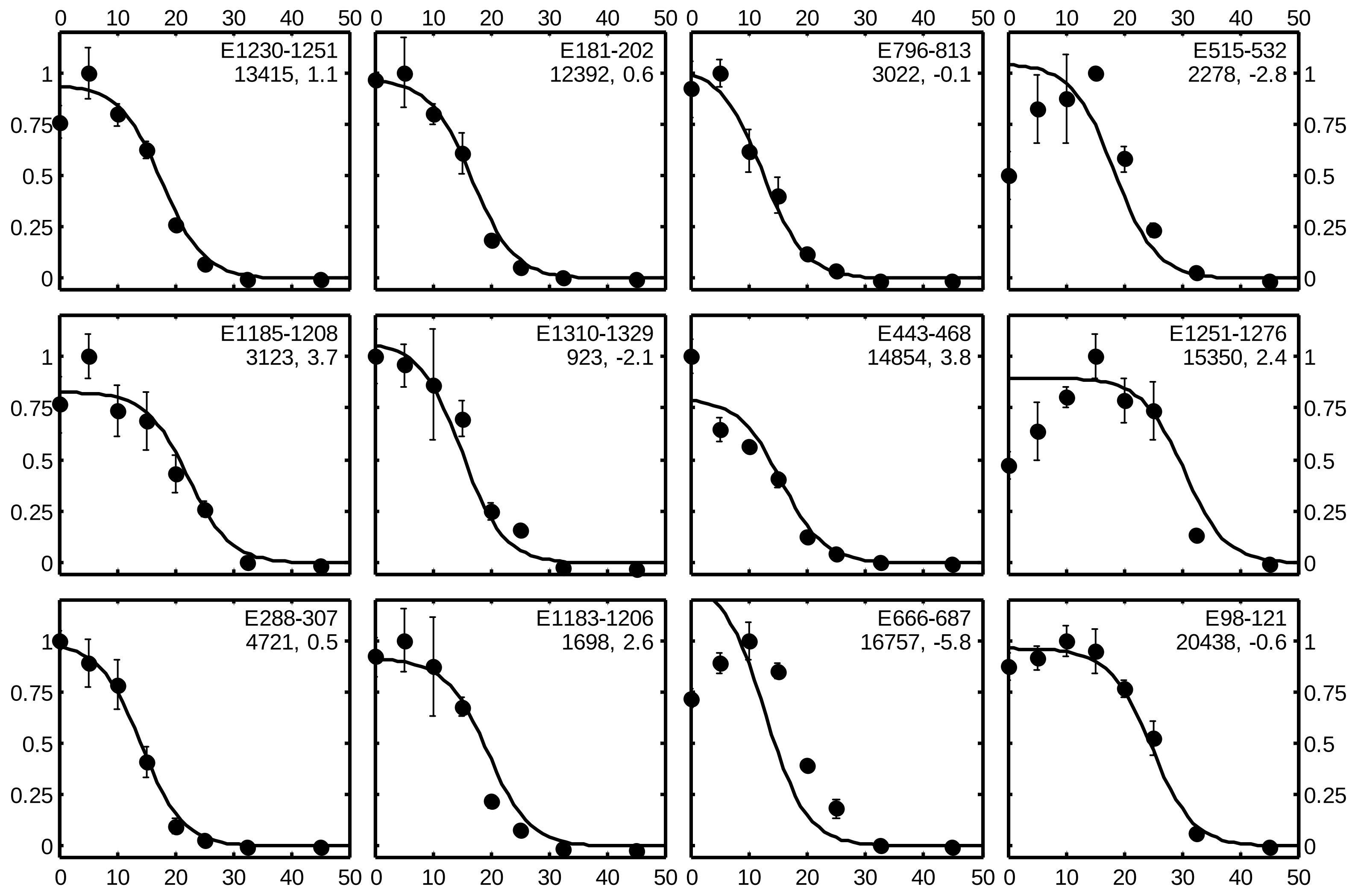

Probes 33-44:

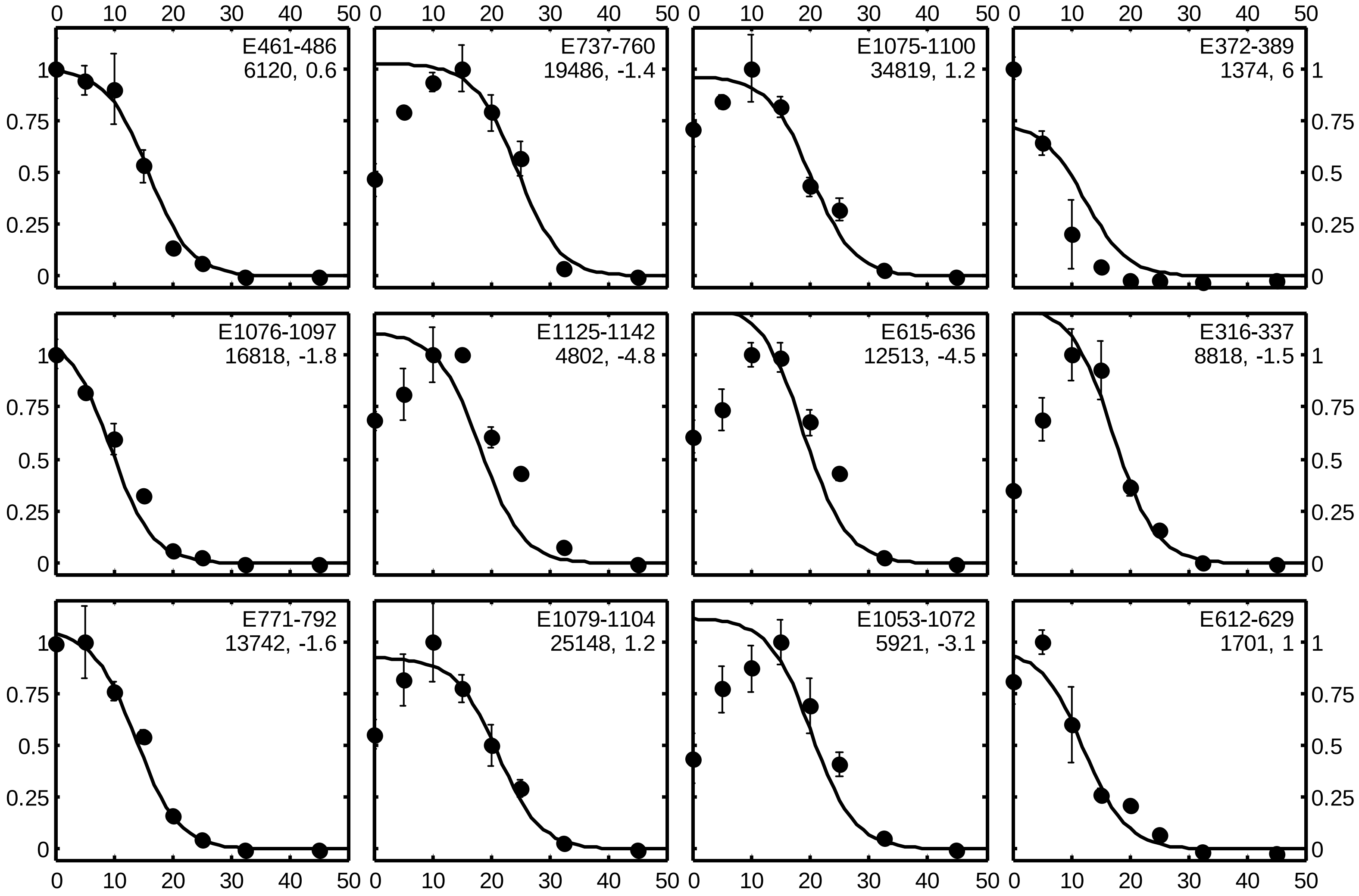
E461-486
6120, 0.6
E737-760
19486, -1.4
E1075-1100
34819, 1.2
E372-389
1374, 6
E1076-1097
16818, -1.8
E1125-1142
4802, -4.8
E615-636
12513, -4.5
E316-337
8818, -1.5
E771-792
13742, -1.6
E1079-1104
25148, 1.2
E1053-1072
5921, -3.1
E612-629
1701, 1
0
10
20
30
40
50
0
0.25
0.5
0.75
1

Figure S1A

Probes 45-56:

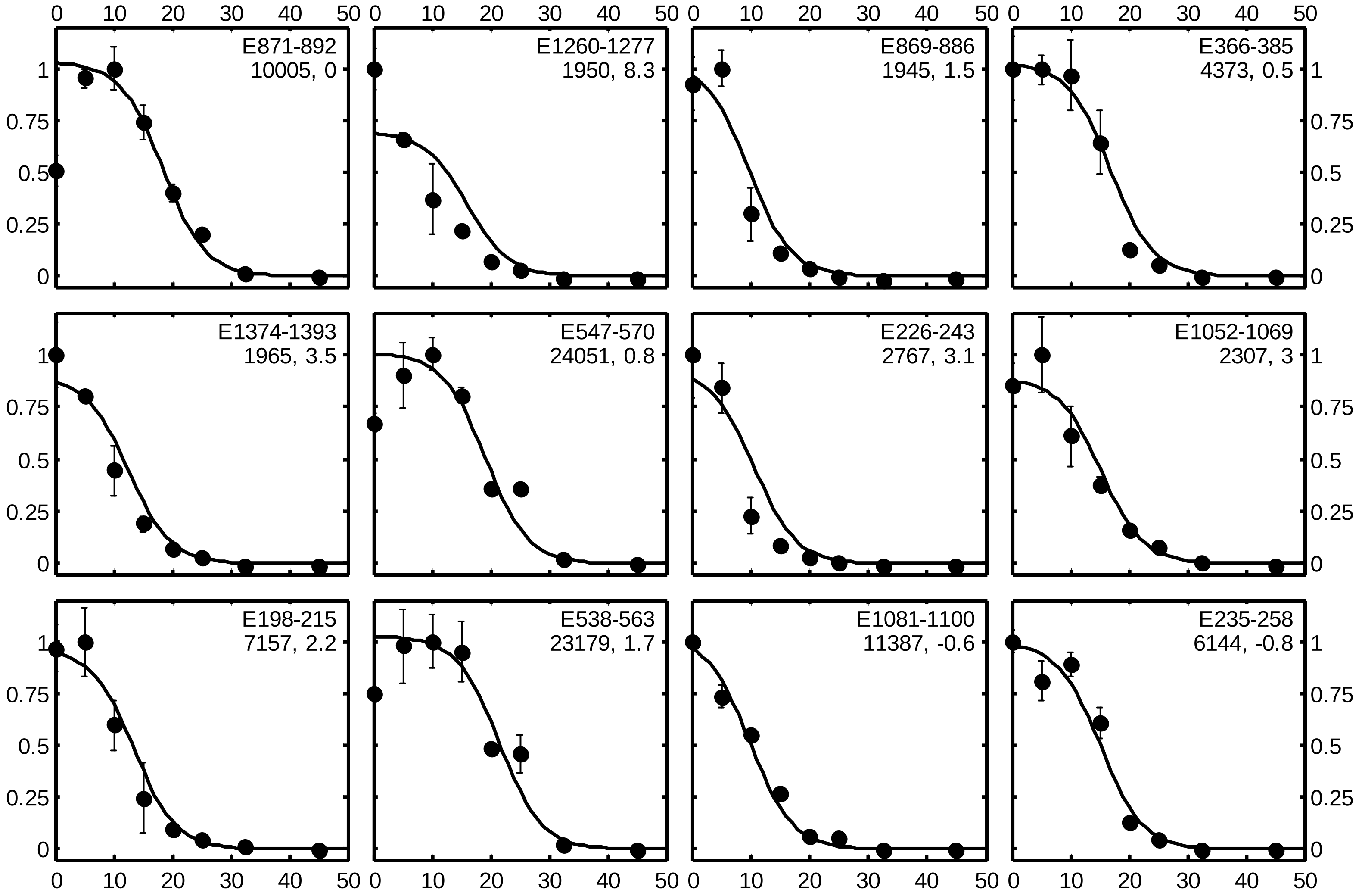

Figure S1A

Probes 57-68:

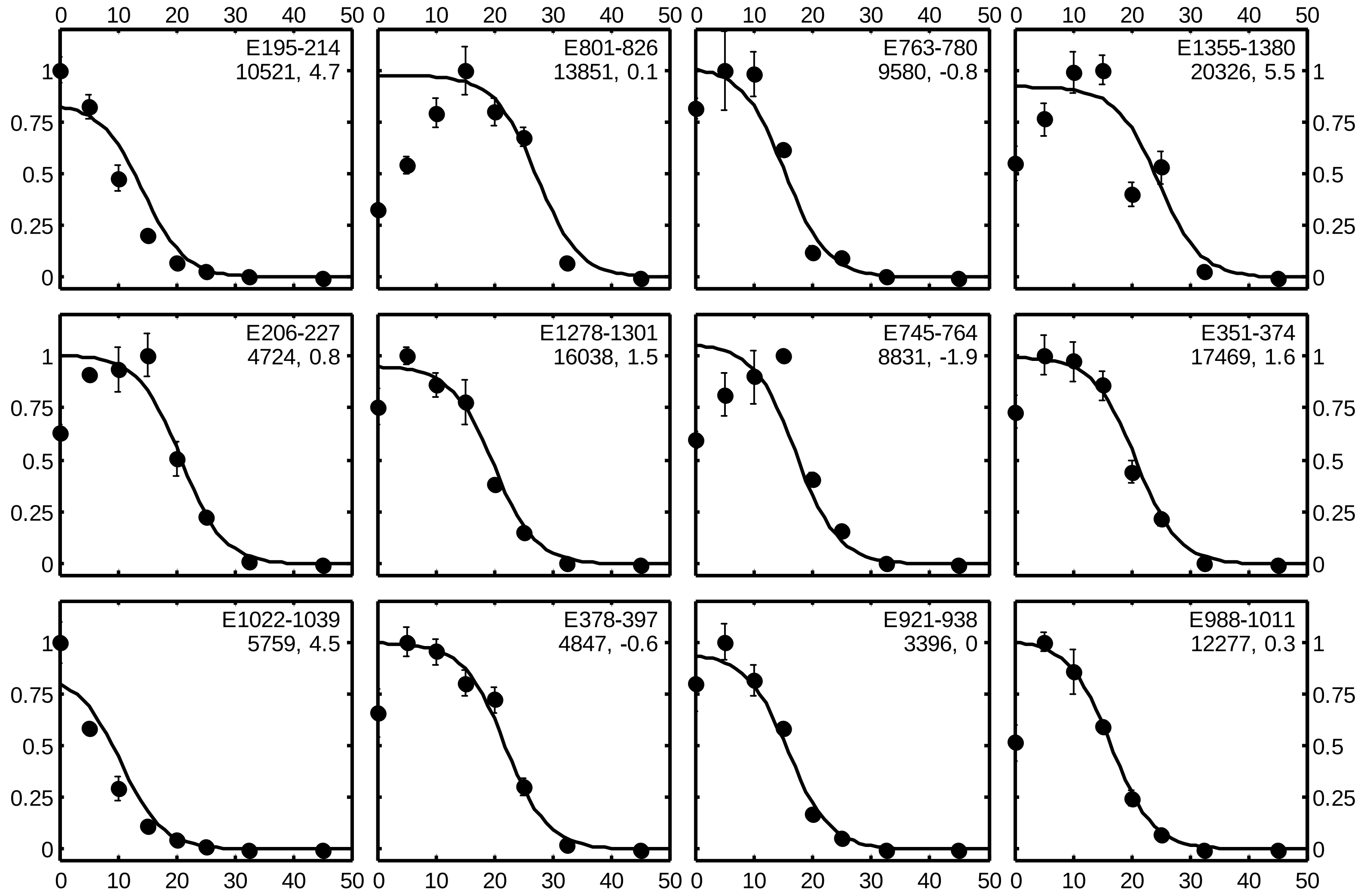

Figure S1A

Probes 69-80:

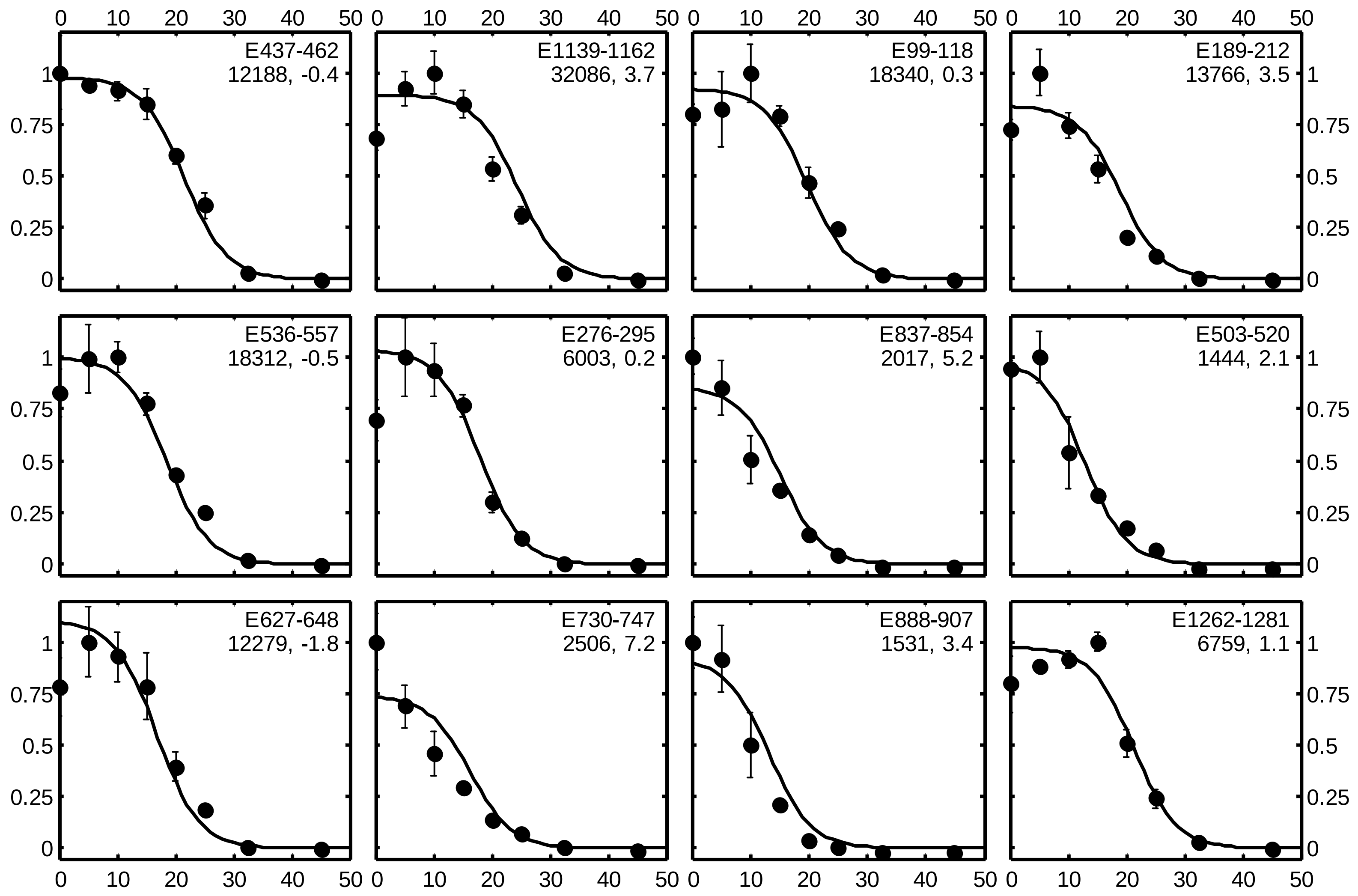

Figure S1A

Probes 81-92:

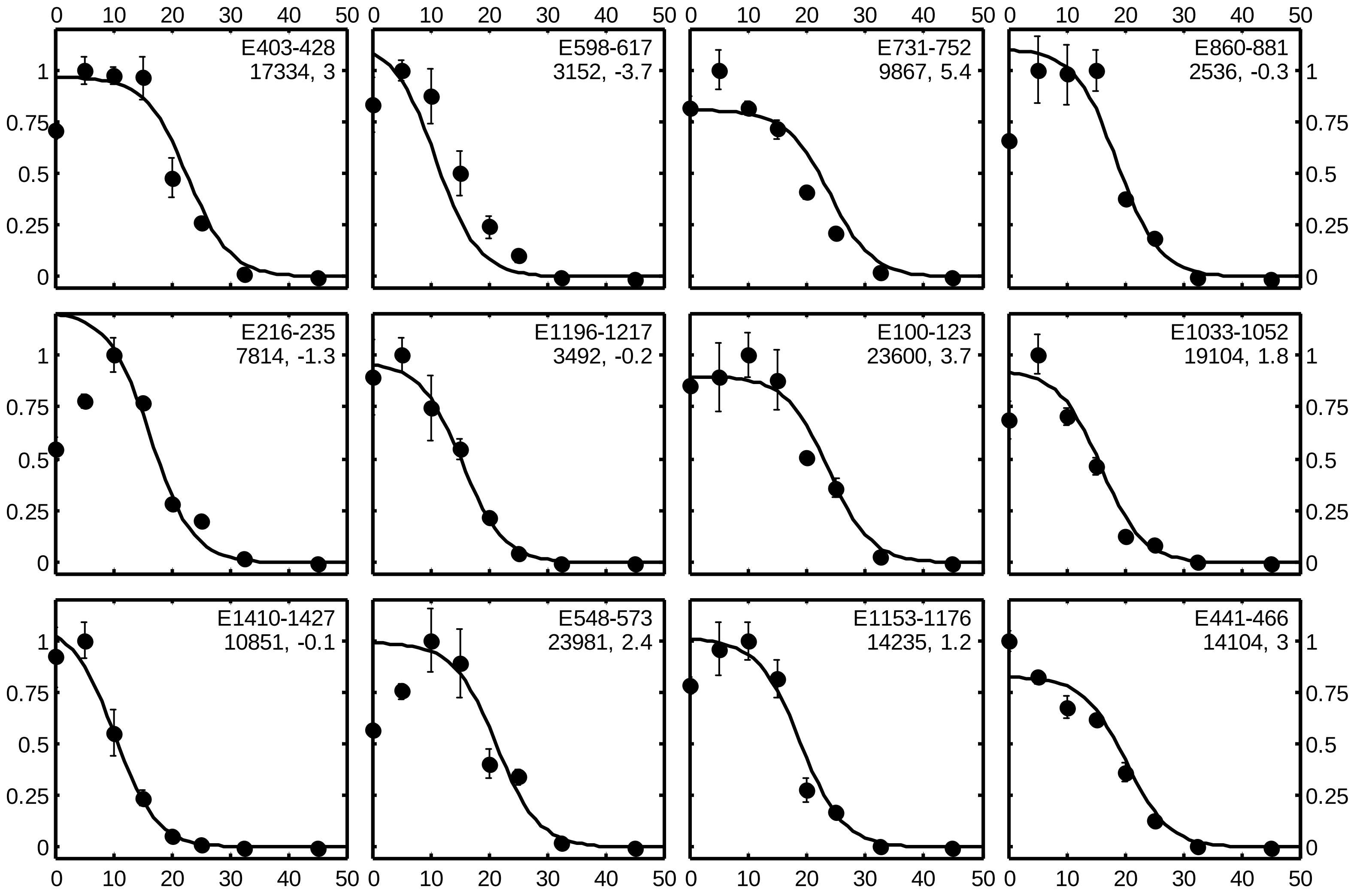

Probes 93-100:

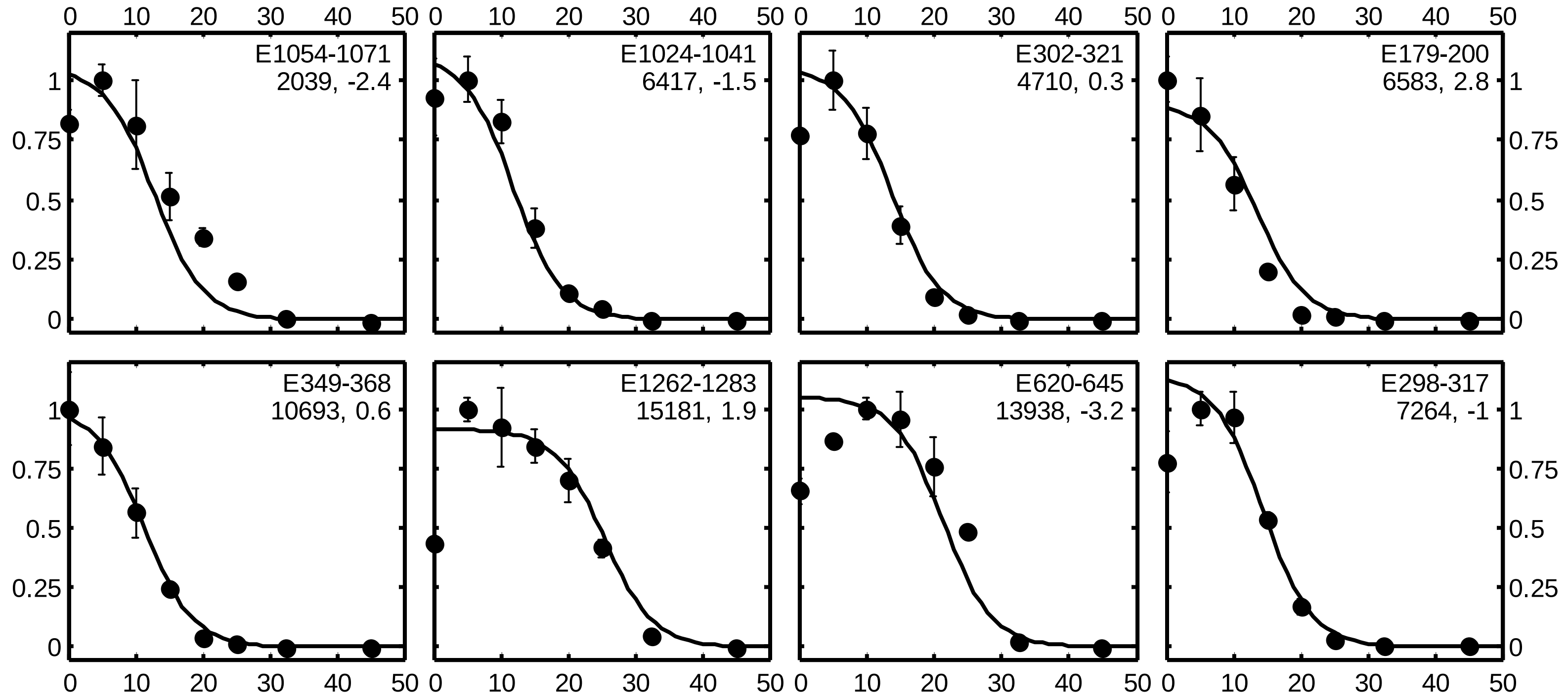

Figure S1B. Experimental (circles) and theoretical (curves) formamide dissociation profiles of 100 randomly selected probes from OneM set (Table 2). The labels (upper right corner of each graph) indicate probe name (top), I_{max} (bottom, first number), and err[FA]$_{1/2}$ value (bottom, second number). See main manuscript for the descriptions of probe names and I_{max} and err[FA]$_{1/2}$ values. x-axis, formamide concentration; y-axis, normalized signal intensity; error bars, standard deviations.

Probes 1-8:

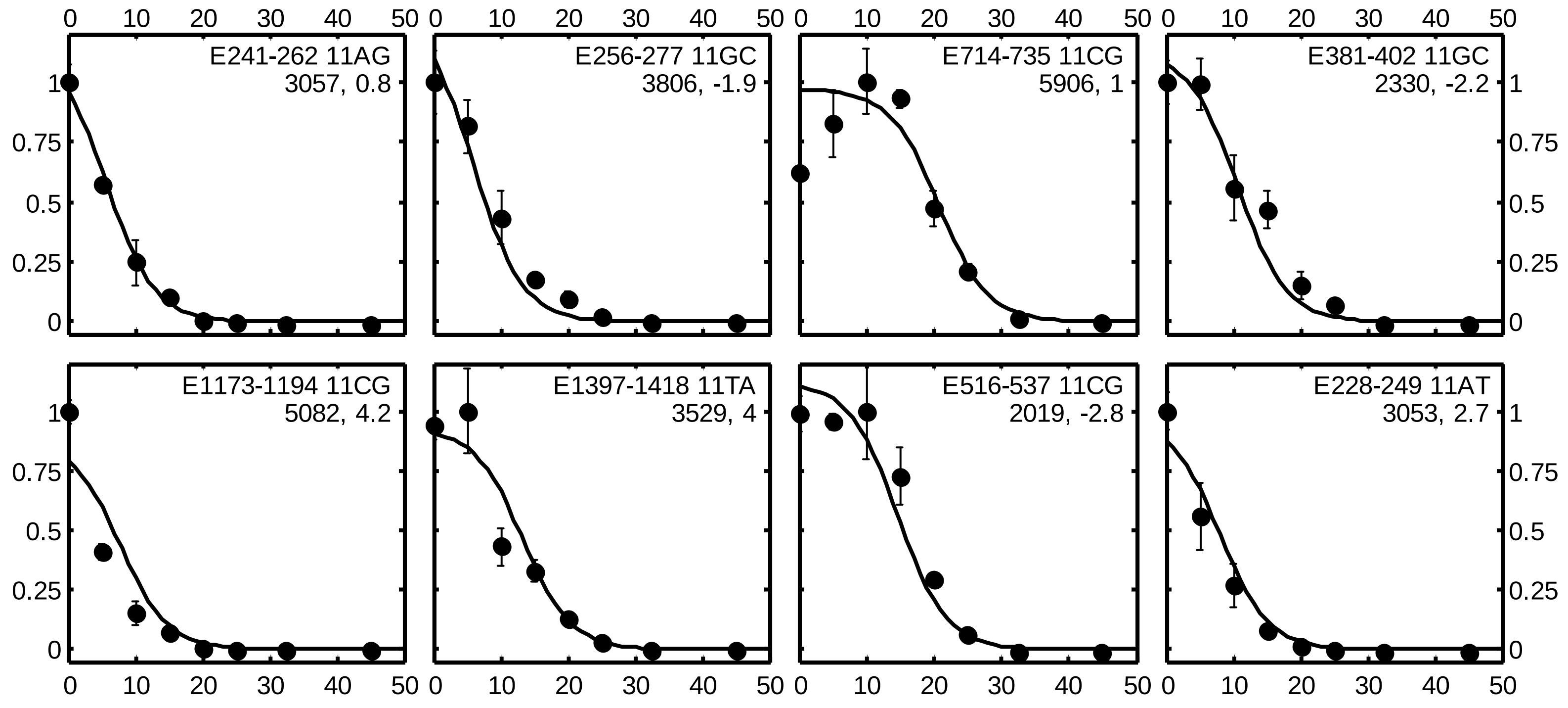

Probes 9-20:

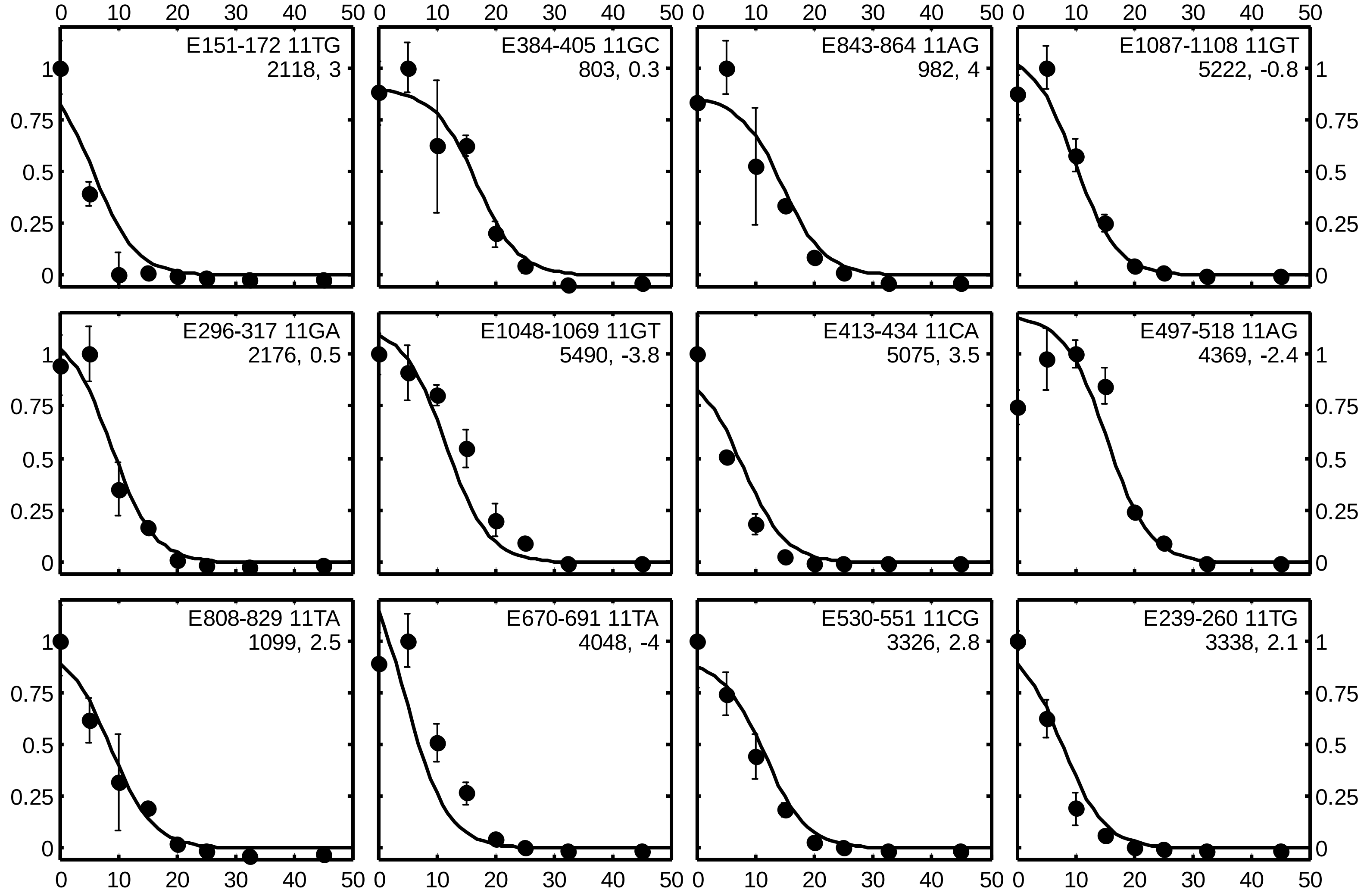
E151-172 11TG
2118, 3
E384-405 11GC
803, 0.3
E843-864 11AG
982, 4
E1087-1108 11GT
5222, -0.8
E296-317 11GA
2176, 0.5
E1048-1069 11GT
5490, -3.8
E413-434 11CA
5075, 3.5
E497-518 11AG
4369, -2.4
E808-829 11TA
1099, 2.5
E670-691 11TA
4048, -4
E530-551 11CG
3326, 2.8
E239-260 11TG
3338, 2.1
0 10 20 30 40 50
0 0.25 0.5 0.75 1

Probes 21-32:

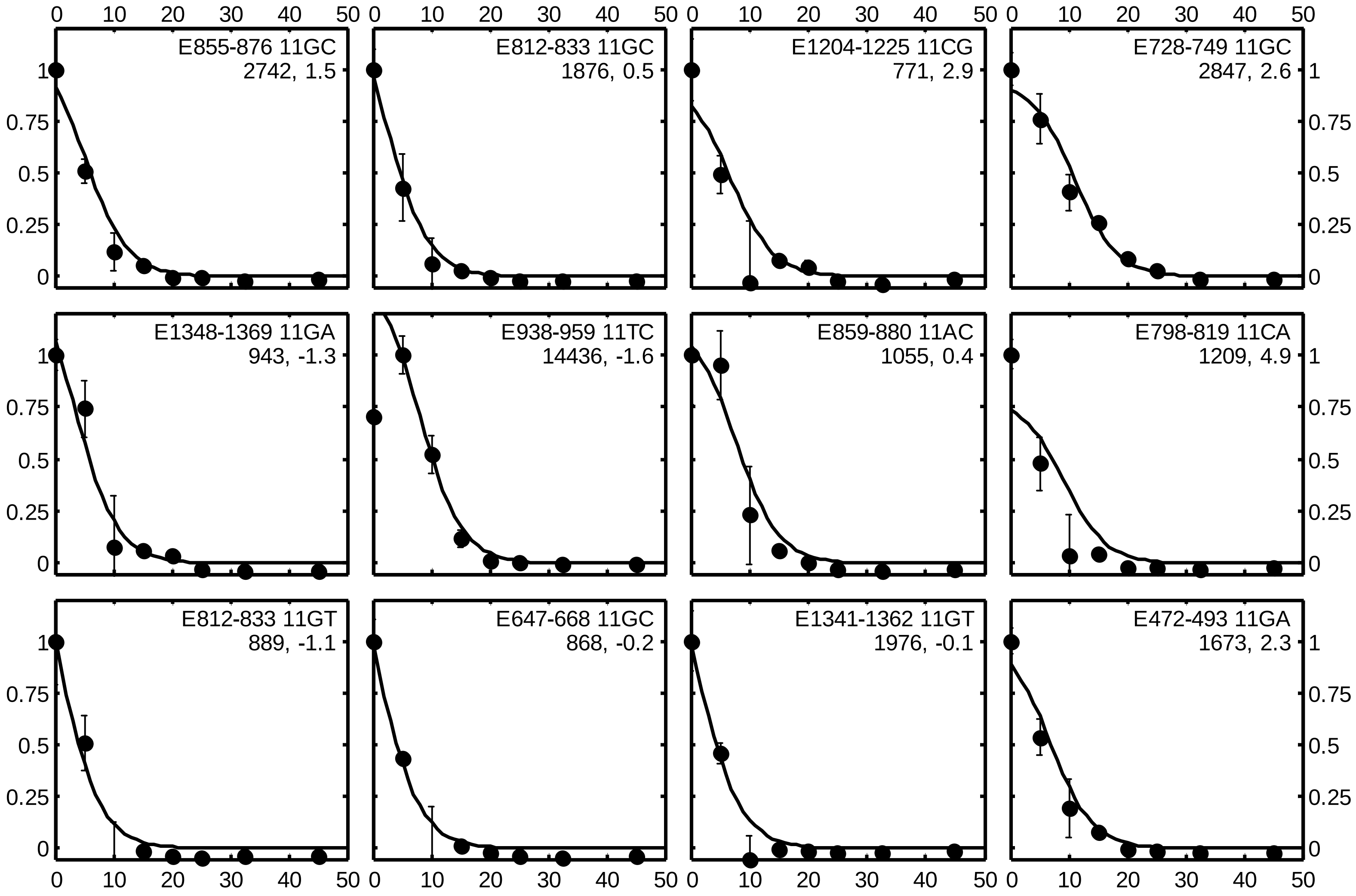
E855-876 11GC
2742, 1.5
E812-833 11GC
1876, 0.5
E1204-1225 11CG
771, 2.9
E728-749 11GC
2847, 2.6
E1348-1369 11GA
943, -1.3
E938-959 11TC
14436, -1.6
E859-880 11AC
1055, 0.4
E798-819 11CA
1209, 4.9
E812-833 11GT
889, -1.1
E647-668 11GC
868, -0.2
E1341-1362 11GT
1976, -0.1
E472-493 11GA
1673, 2.3
0
10
20
30
40
50
0
0.25
0.5
0.75
1

Figure S1B

Probes 33-44:

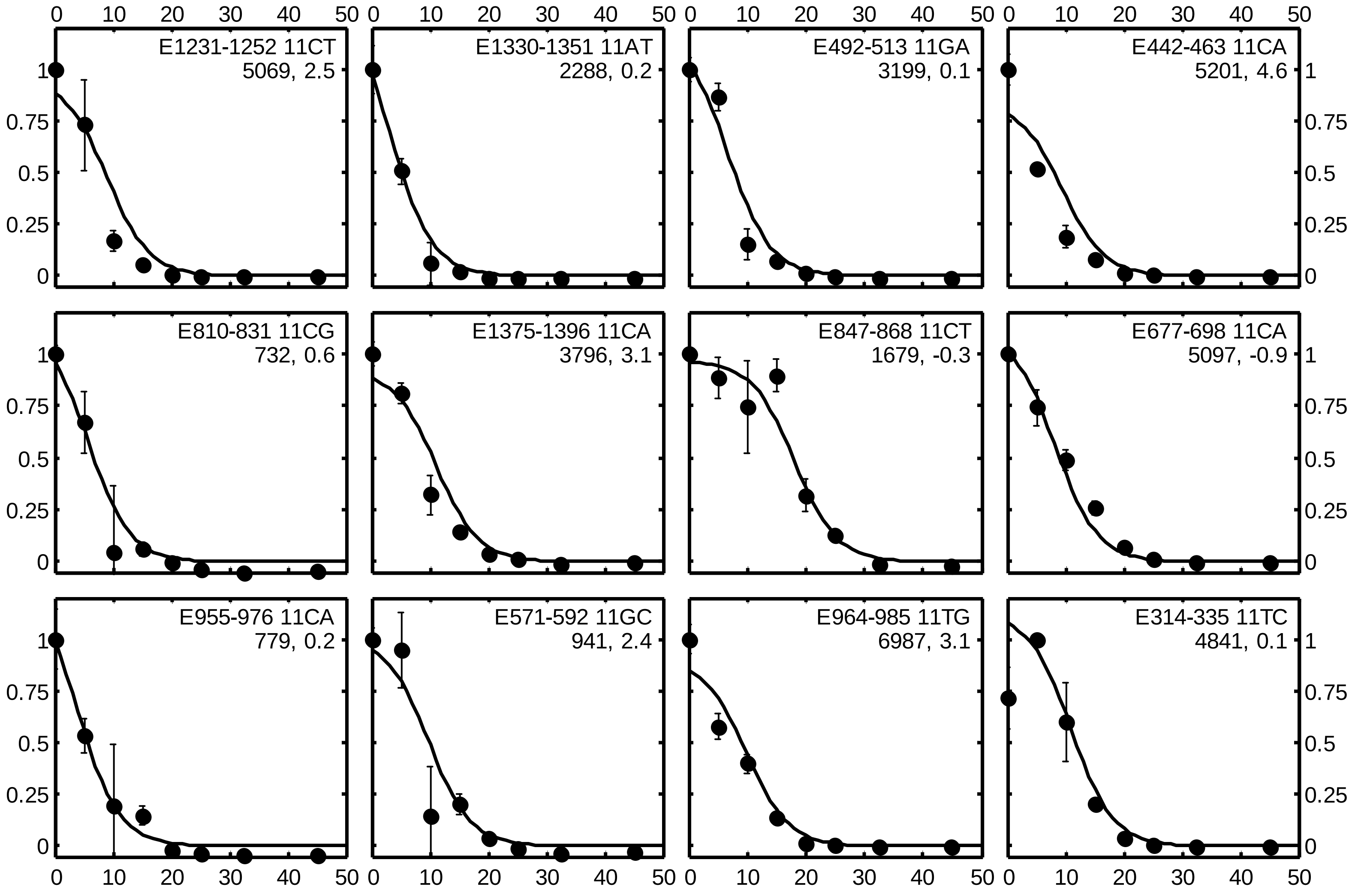

Probes 45-56:

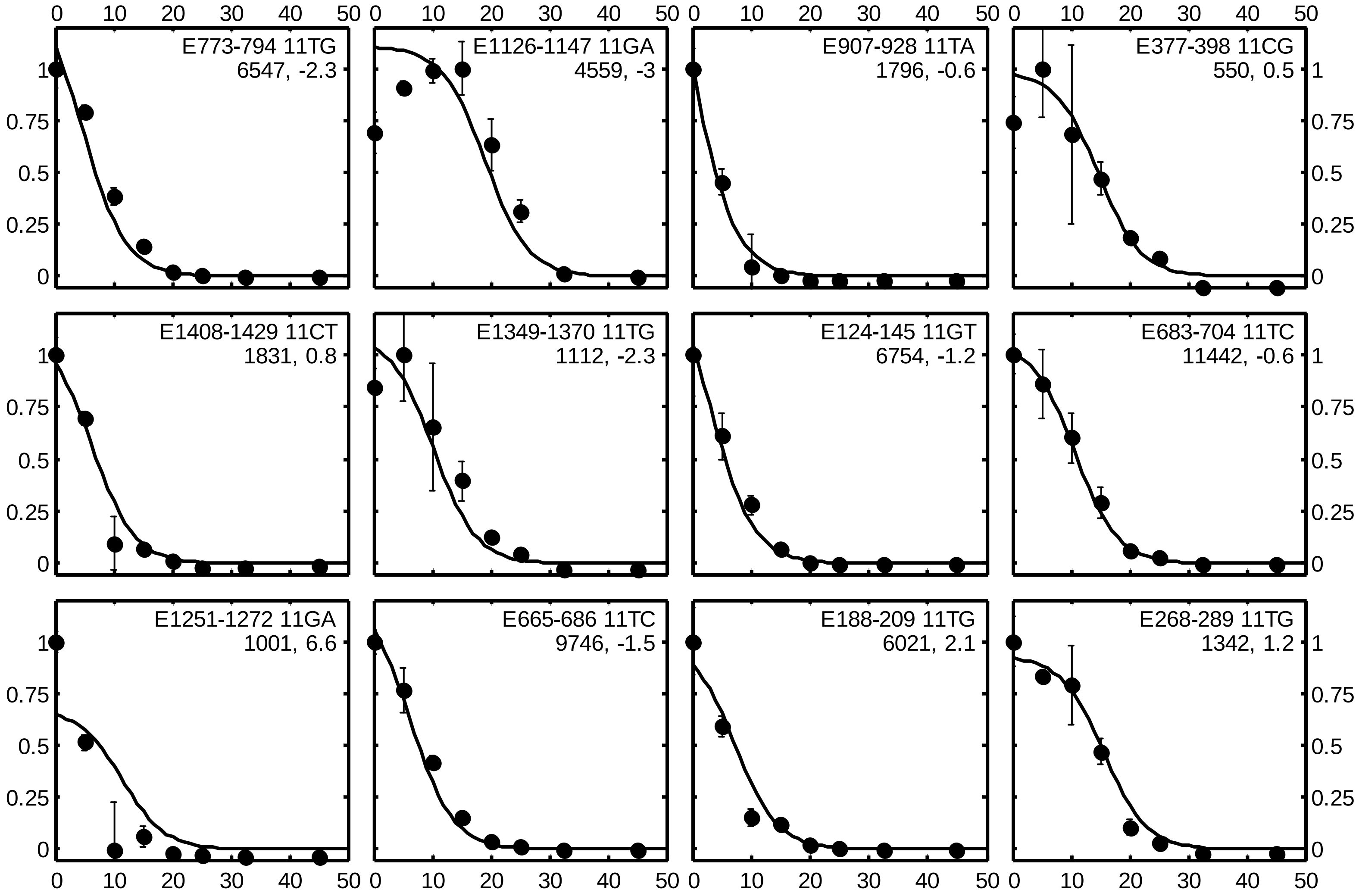
E773-794 11TG
6547, -2.3
E1126-1147 11GA
4559, -3
E907-928 11TA
1796, -0.6
E377-398 11CG
550, 0.5
E1408-1429 11CT
1831, 0.8
E1349-1370 11TG
1112, -2.3
E124-145 11GT
6754, -1.2
E683-704 11TC
11442, -0.6
E1251-1272 11GA
1001, 6.6
E665-686 11TC
9746, -1.5
E188-209 11TG
6021, 2.1
E268-289 11TG
1342, 1.2

Figure S1B

Probes 57-68:

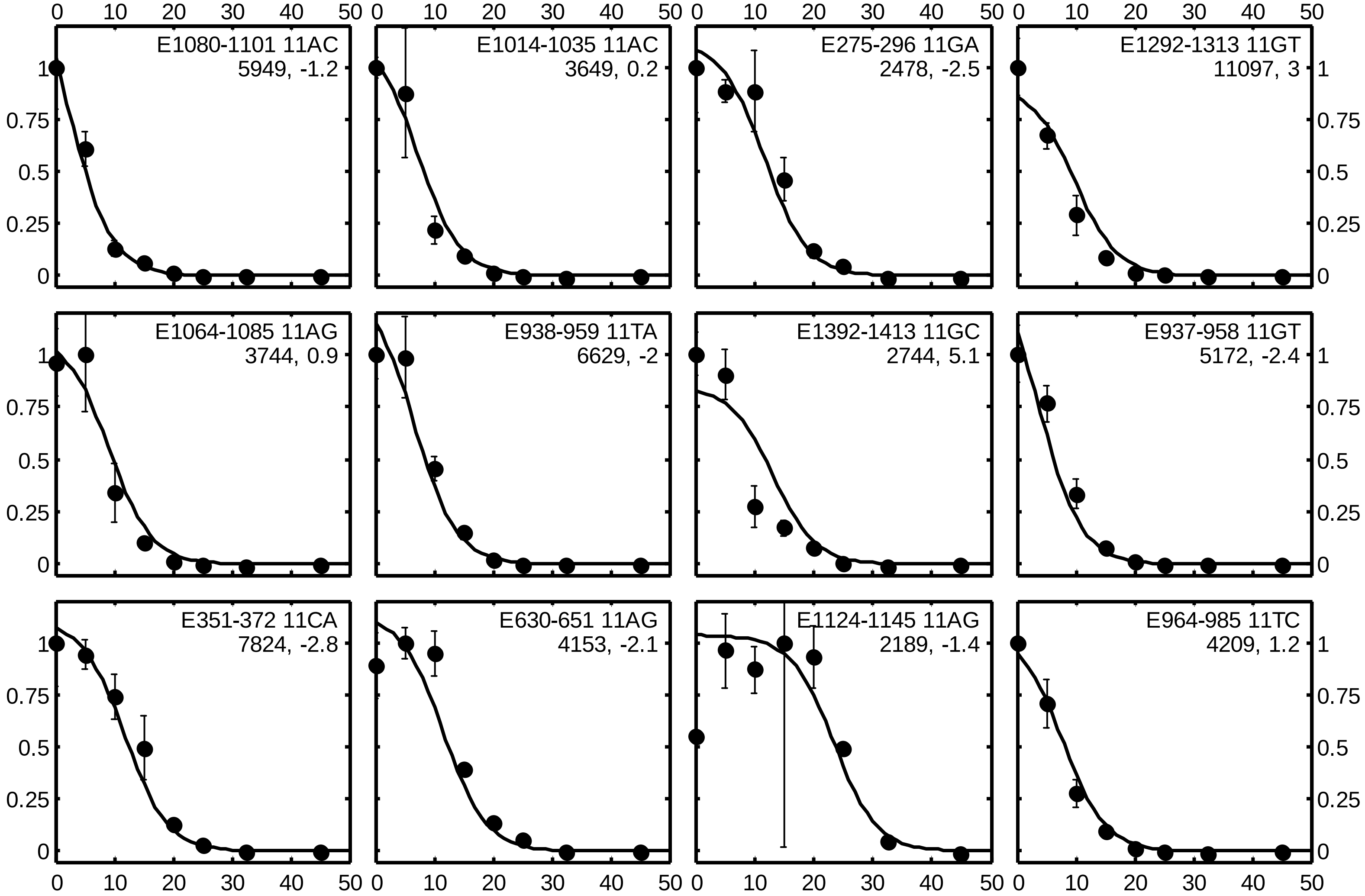

Probes 69-80:

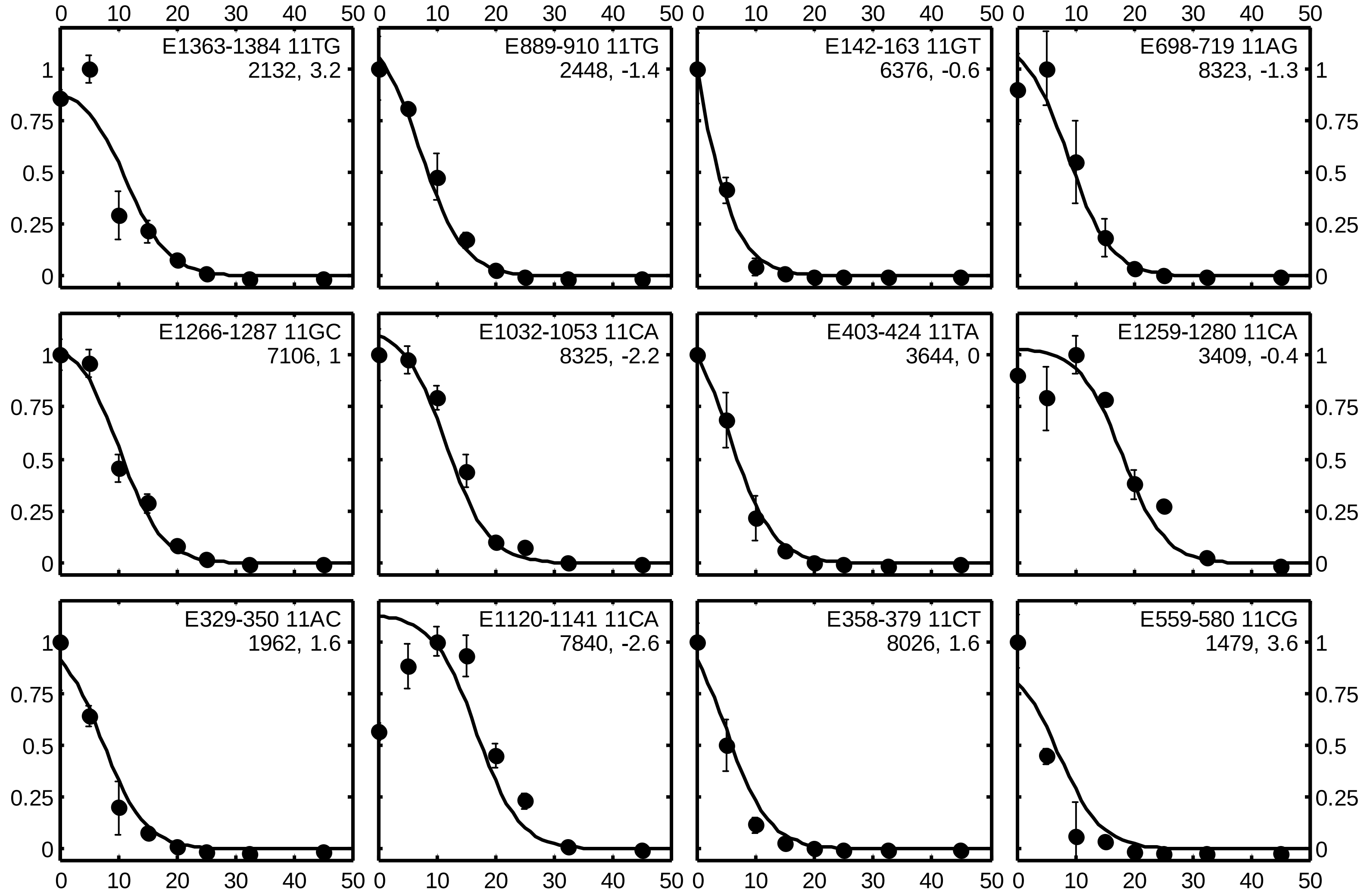
E1363-1384 11TG
2132, 3.2
E889-910 11TG
2448, -1.4
E142-163 11GT
6376, -0.6
E698-719 11AG
8323, -1.3
E1266-1287 11GC
7106, 1
E1032-1053 11CA
8325, -2.2
E403-424 11TA
3644, 0
E1259-1280 11CA
3409, -0.4
E329-350 11AC
1962, 1.6
E1120-1141 11CA
7840, -2.6
E358-379 11CT
8026, 1.6
E559-580 11CG
1479, 3.6
0 10 20 30 40 50
0 0.25 0.5 0.75 1

Probes 81-92:

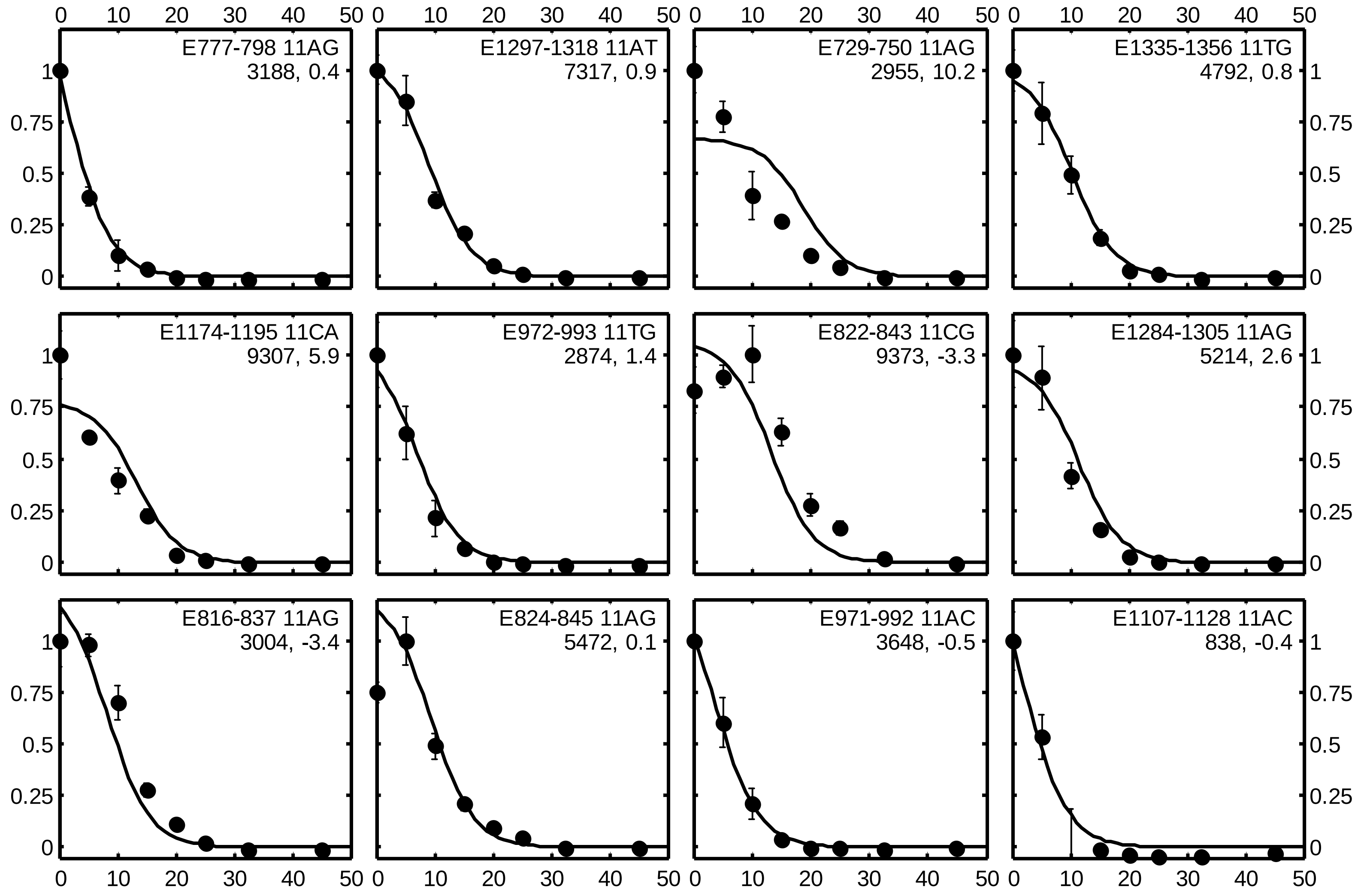
E777-798 11AG
3188, 0.4
E1297-1318 11AT
7317, 0.9
E729-750 11AG
2955, 10.2
E1335-1356 11TG
4792, 0.8
E1174-1195 11CA
9307, 5.9
E972-993 11TG
2874, 1.4
E822-843 11CG
9373, -3.3
E1284-1305 11AG
5214, 2.6
E816-837 11AG
3004, -3.4
E824-845 11AG
5472, 0.1
E971-992 11AC
3648, -0.5
E1107-1128 11AC
838, -0.4

Probes 93-100:

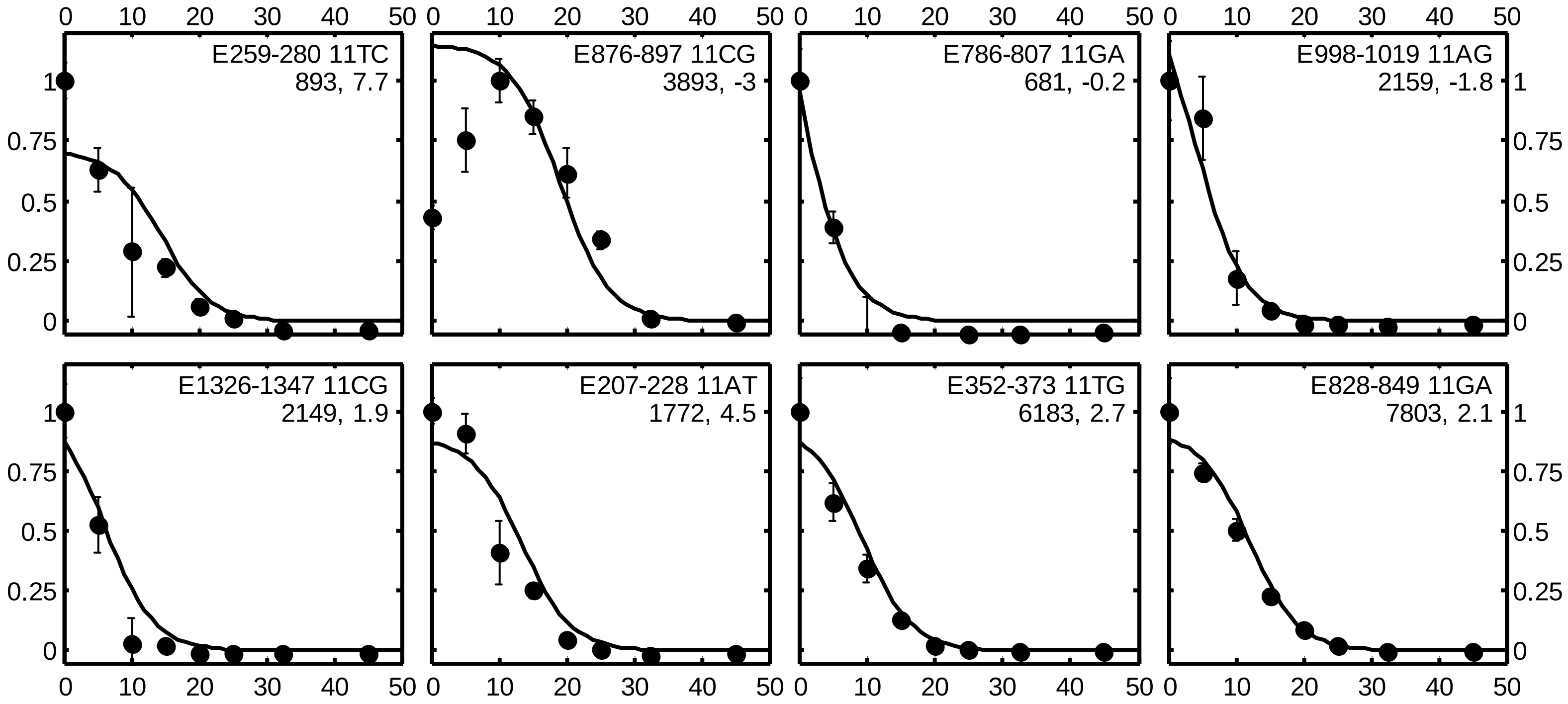

Figure S1C. Experimental (circles) and theoretical (curves) formamide dissociation profiles of 100 randomly selected probes from PosM set (Table 2). The labels (upper right corner of each graph) indicate probe name (top), I_{max} (bottom, first number), and $err[FA]_{1/2}$ value (bottom, second number). See main manuscript for the descriptions of probe names and I_{max} and $err[FA]_{1/2}$ values. x-axis, formamide concentration; y-axis, normalized signal intensity; error bars, standard deviations.

Probes 1-8:

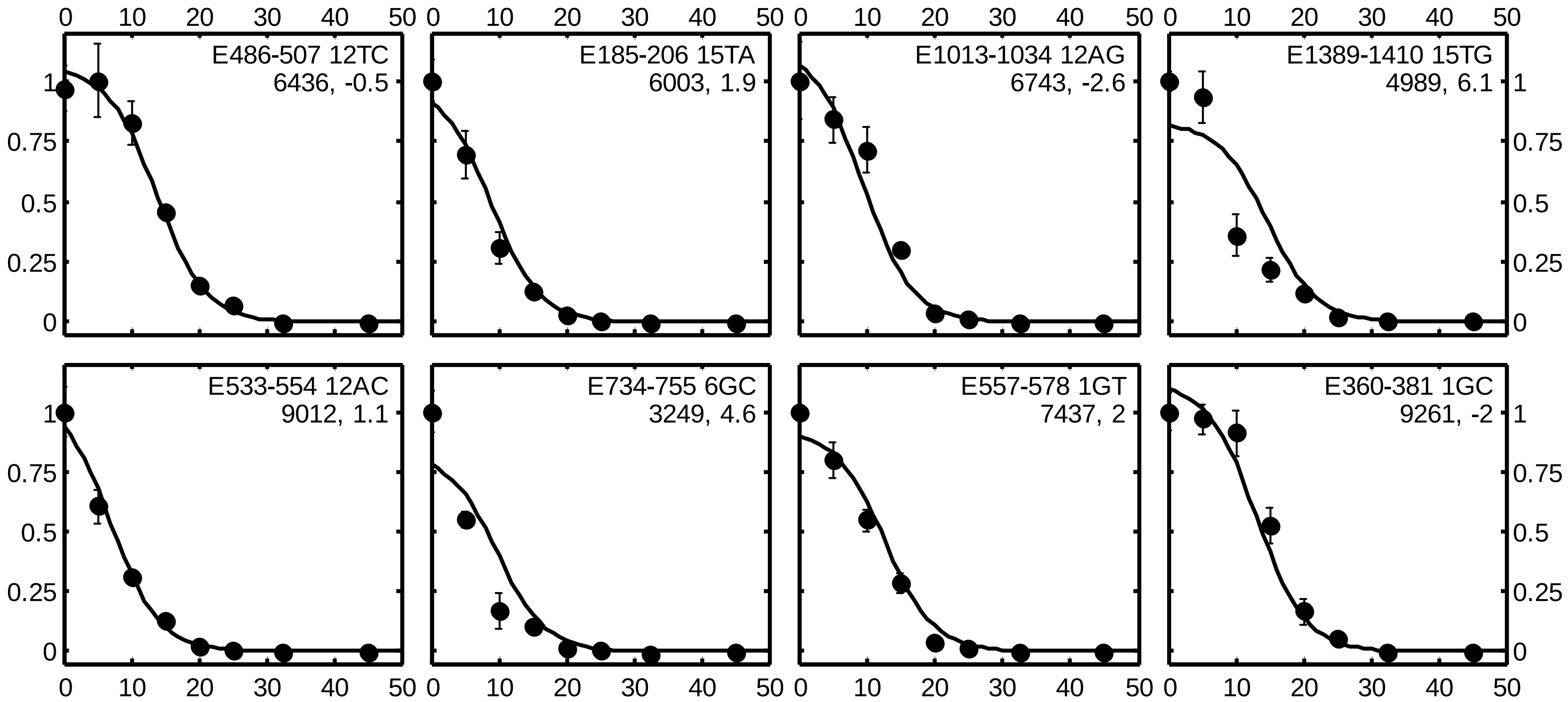

Figure S1C

Probes 9-20:

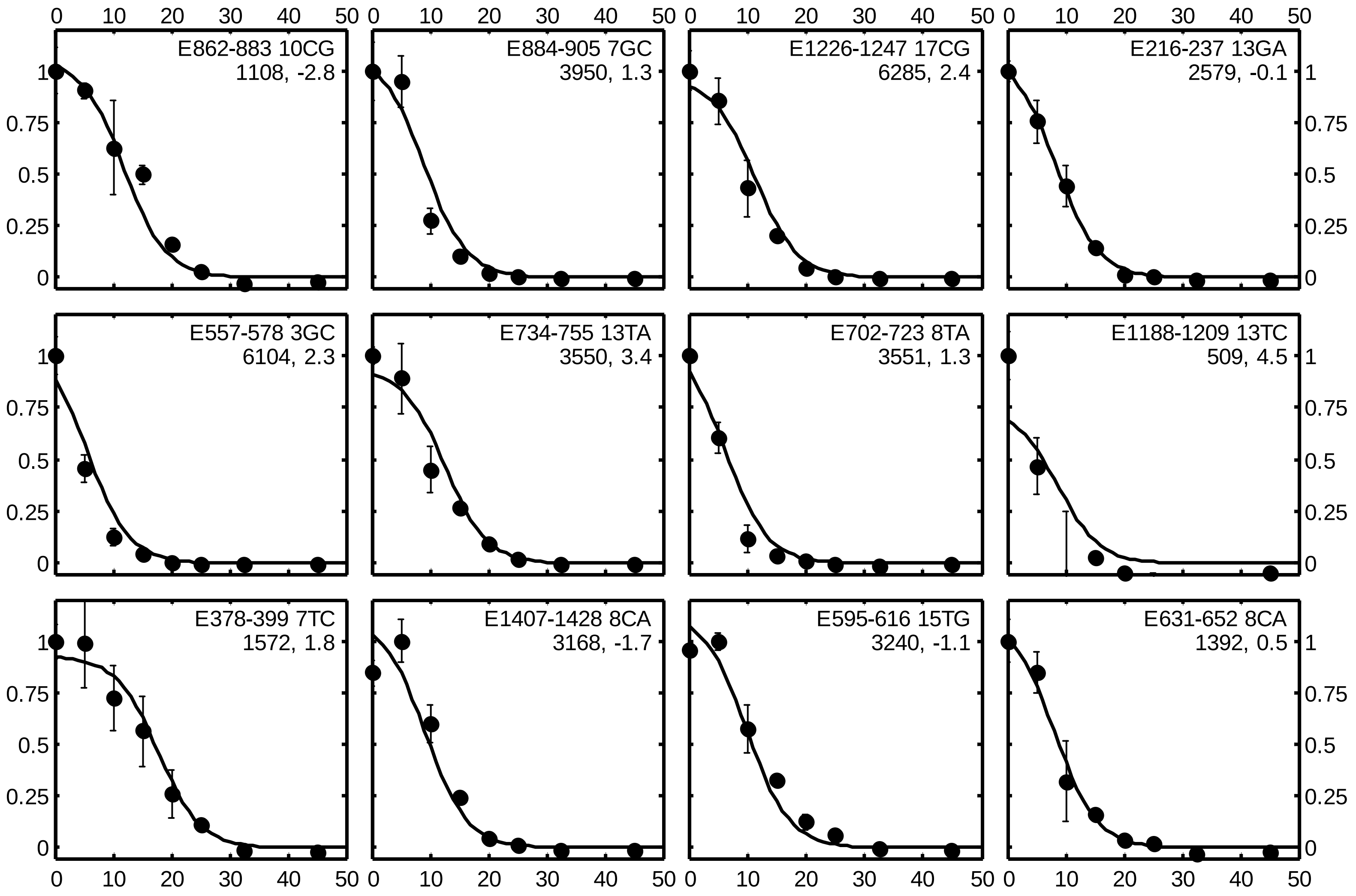

Figure S1C

Probes 21-32:

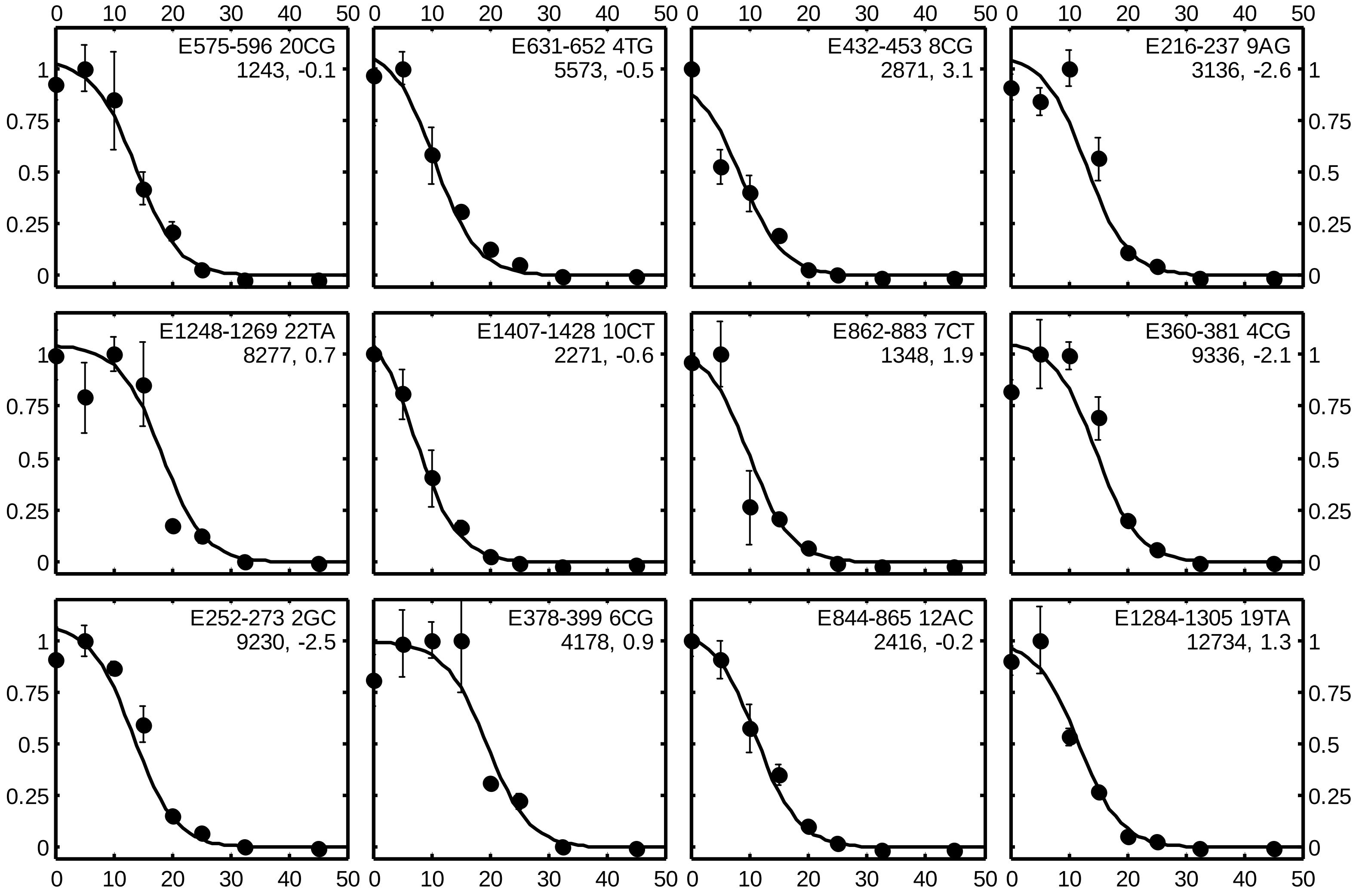

Probes 33-44:

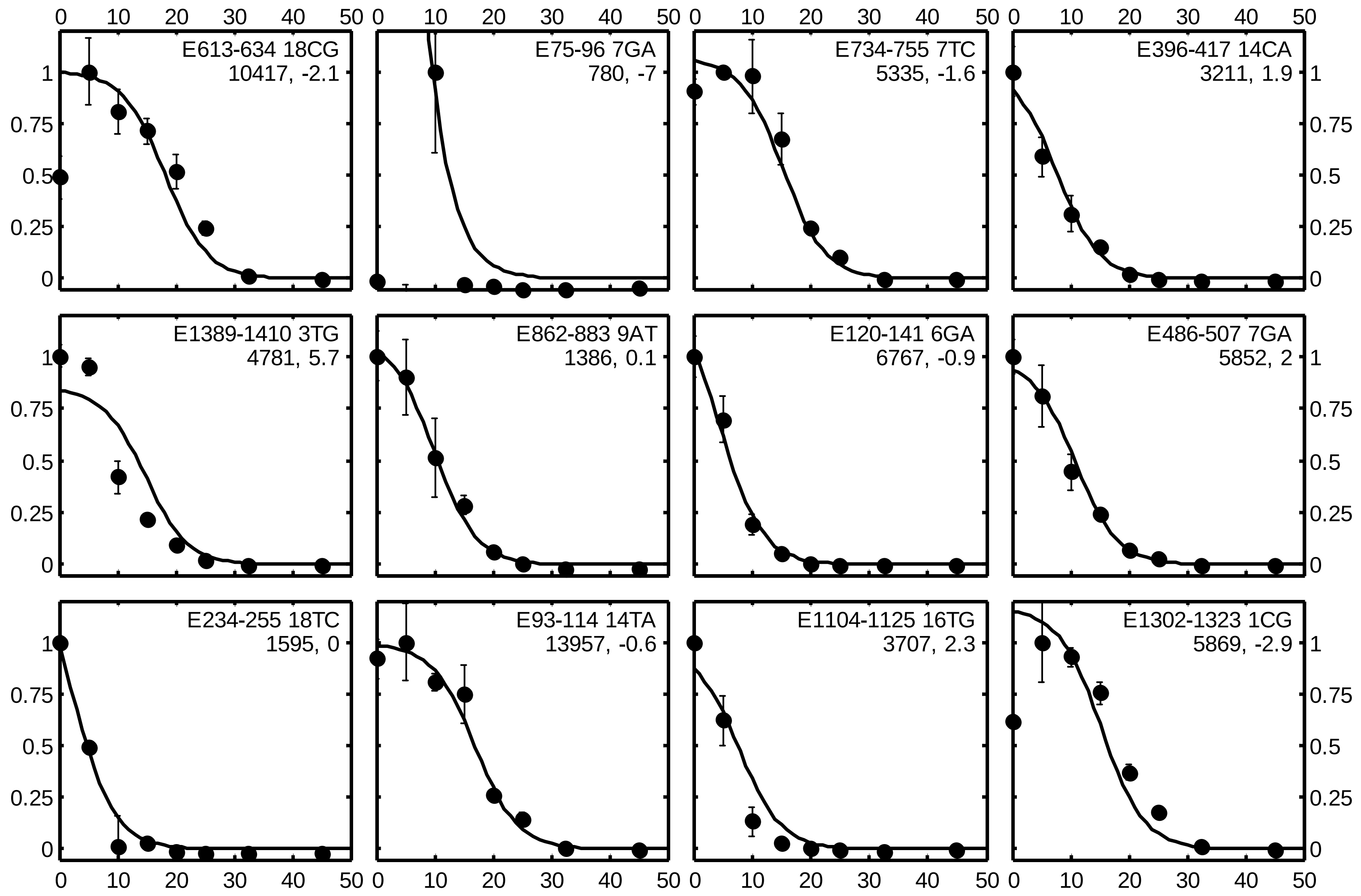
E613-634 18CG
10417, -2.1
E75-96 7GA
780, -7
E734-755 7TC
5335, -1.6
E396-417 14CA
3211, 1.9
E1389-1410 3TG
4781, 5.7
E862-883 9AT
1386, 0.1
E120-141 6GA
6767, -0.9
E486-507 7GA
5852, 2
E234-255 18TC
1595, 0
E93-114 14TA
13957, -0.6
E1104-1125 16TG
3707, 2.3
E1302-1323 1CG
5869, -2.9

Probes 45-56:

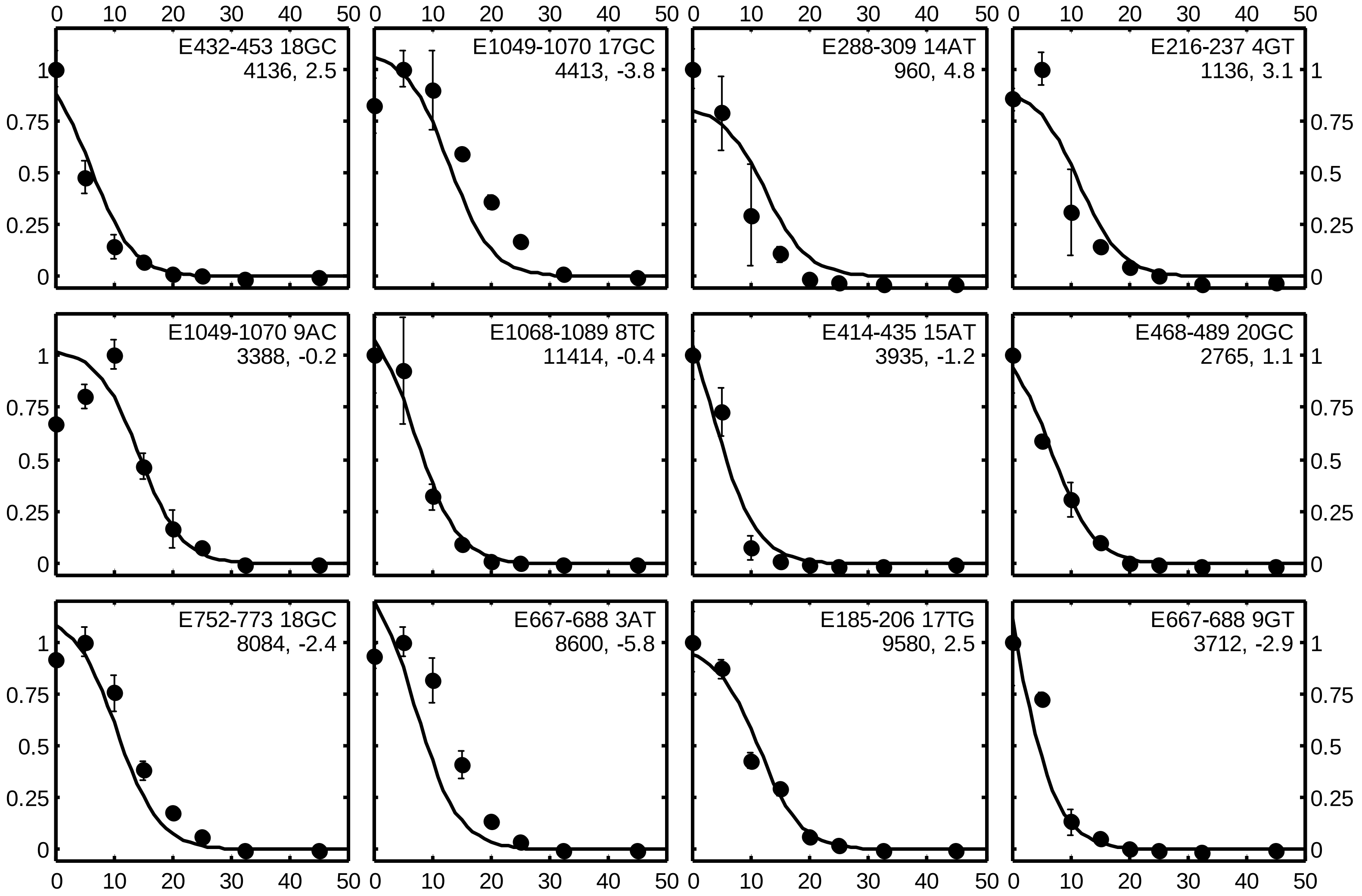
E432-453 18GC
4136, 2.5
E1049-1070 17GC
4413, -3.8
E288-309 14AT
960, 4.8
E216-237 4GT
1136, 3.1
E1049-1070 9AC
3388, -0.2
E1068-1089 8TC
11414, -0.4
E414-435 15AT
3935, -1.2
E468-489 20GC
2765, 1.1
E752-773 18GC
8084, -2.4
E667-688 3AT
8600, -5.8
E185-206 17TG
9580, 2.5
E667-688 9GT
3712, -2.9

Probes 57-68:

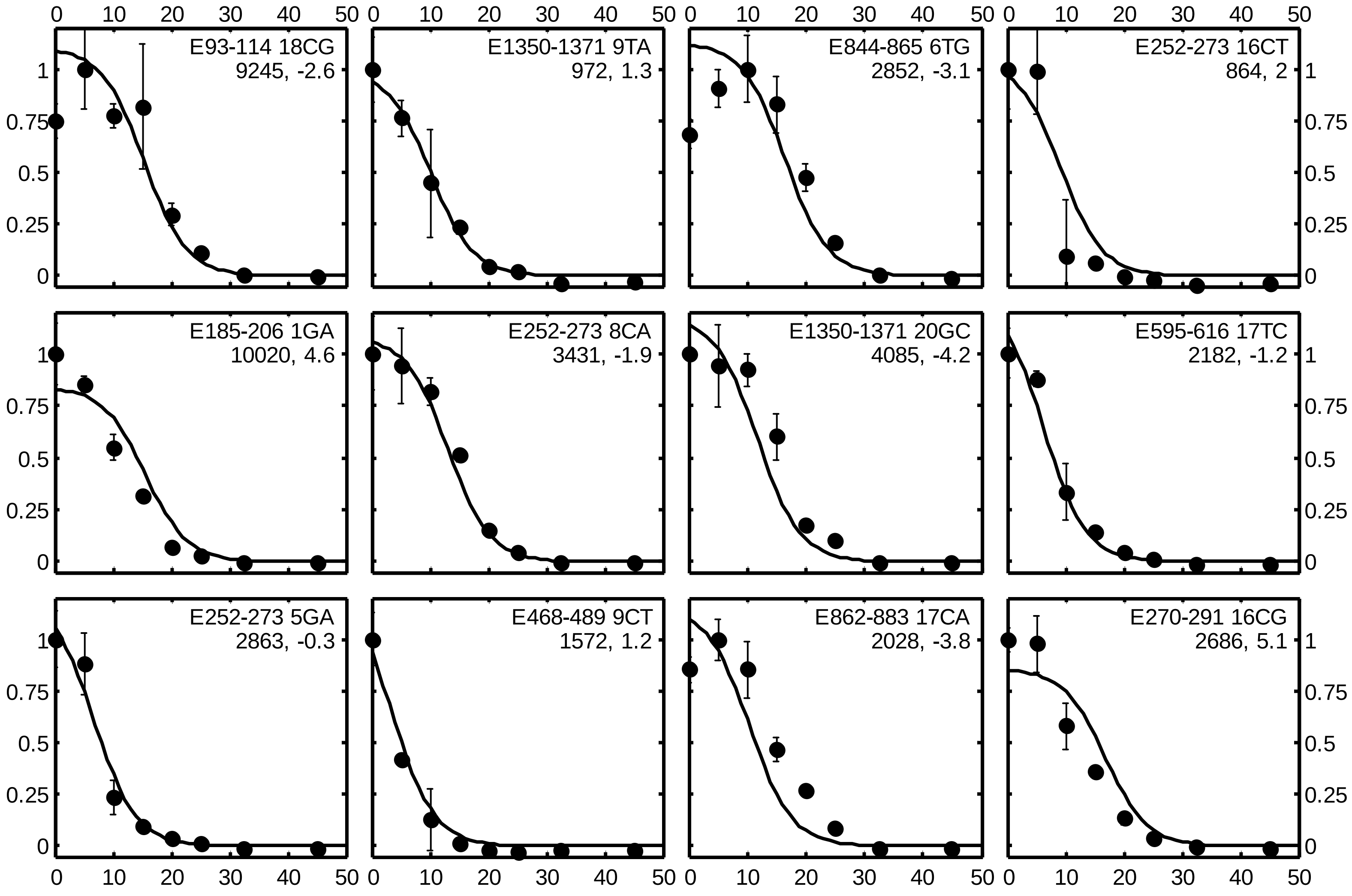
E93-114 18CG
9245, -2.6
E1350-1371 9TA
972, 1.3
E844-865 6TG
2852, -3.1
E252-273 16CT
864, 2
E185-206 1GA
10020, 4.6
E252-273 8CA
3431, -1.9
E1350-1371 20GC
4085, -4.2
E595-616 17TC
2182, -1.2
E252-273 5GA
2863, -0.3
E468-489 9CT
1572, 1.2
E862-883 17CA
2028, -3.8
E270-291 16CG
2686, 5.1
0 10 20 30 40 50
0 0.25 0.5 0.75 1

Probes 69-80:

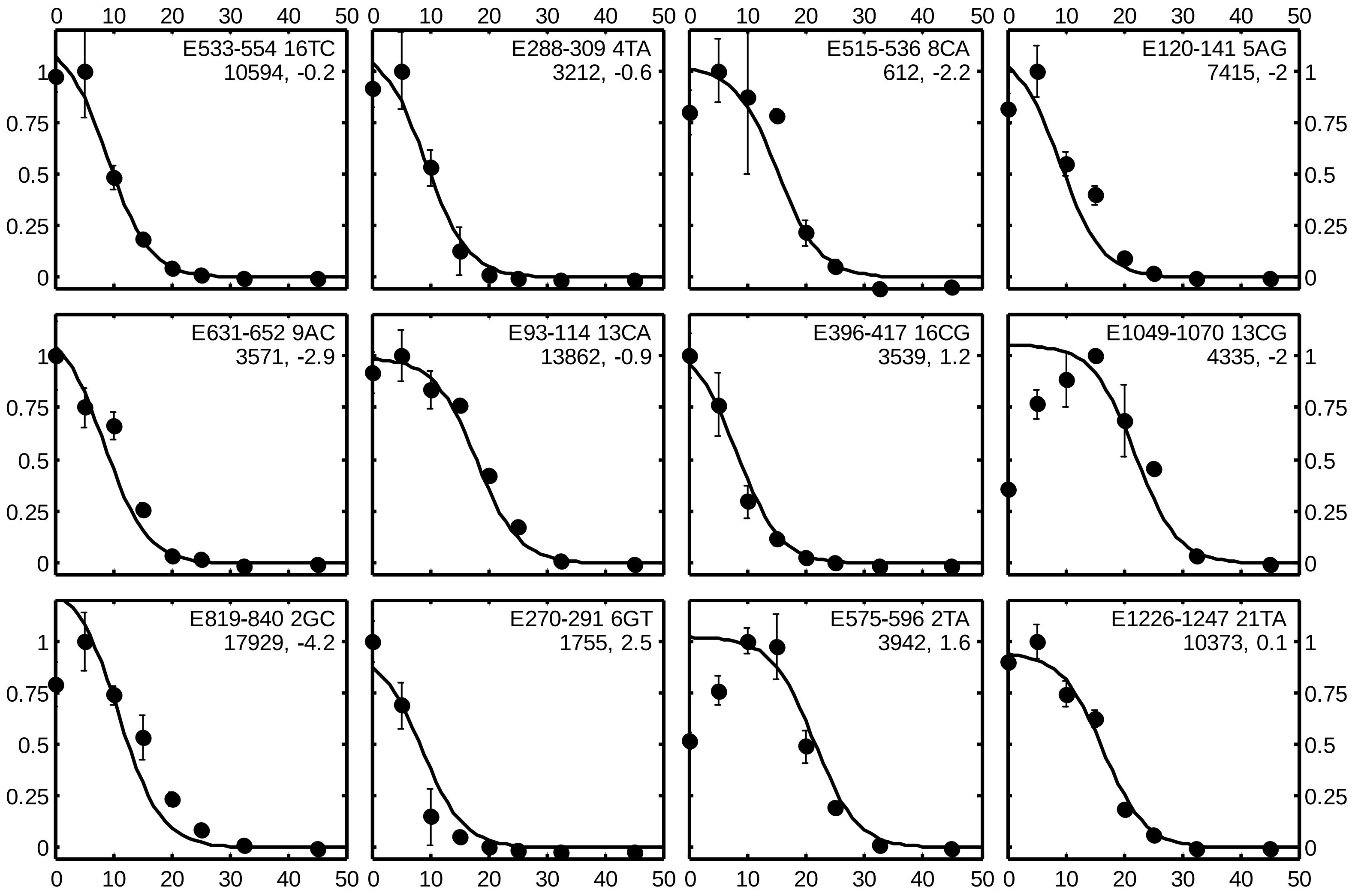
E533-554 16TC
10594, -0.2
E288-309 4TA
3212, -0.6
E515-536 8CA
612, -2.2
E120-141 5AG
7415, -2
E631-652 9AC
3571, -2.9
E93-114 13CA
13862, -0.9
E396-417 16CG
3539, 1.2
E1049-1070 13CG
4335, -2
E819-840 2GC
17929, -4.2
E270-291 6GT
1755, 2.5
E575-596 2TA
3942, 1.6
E1226-1247 21TA
10373, 0.1

Probes 81-92:

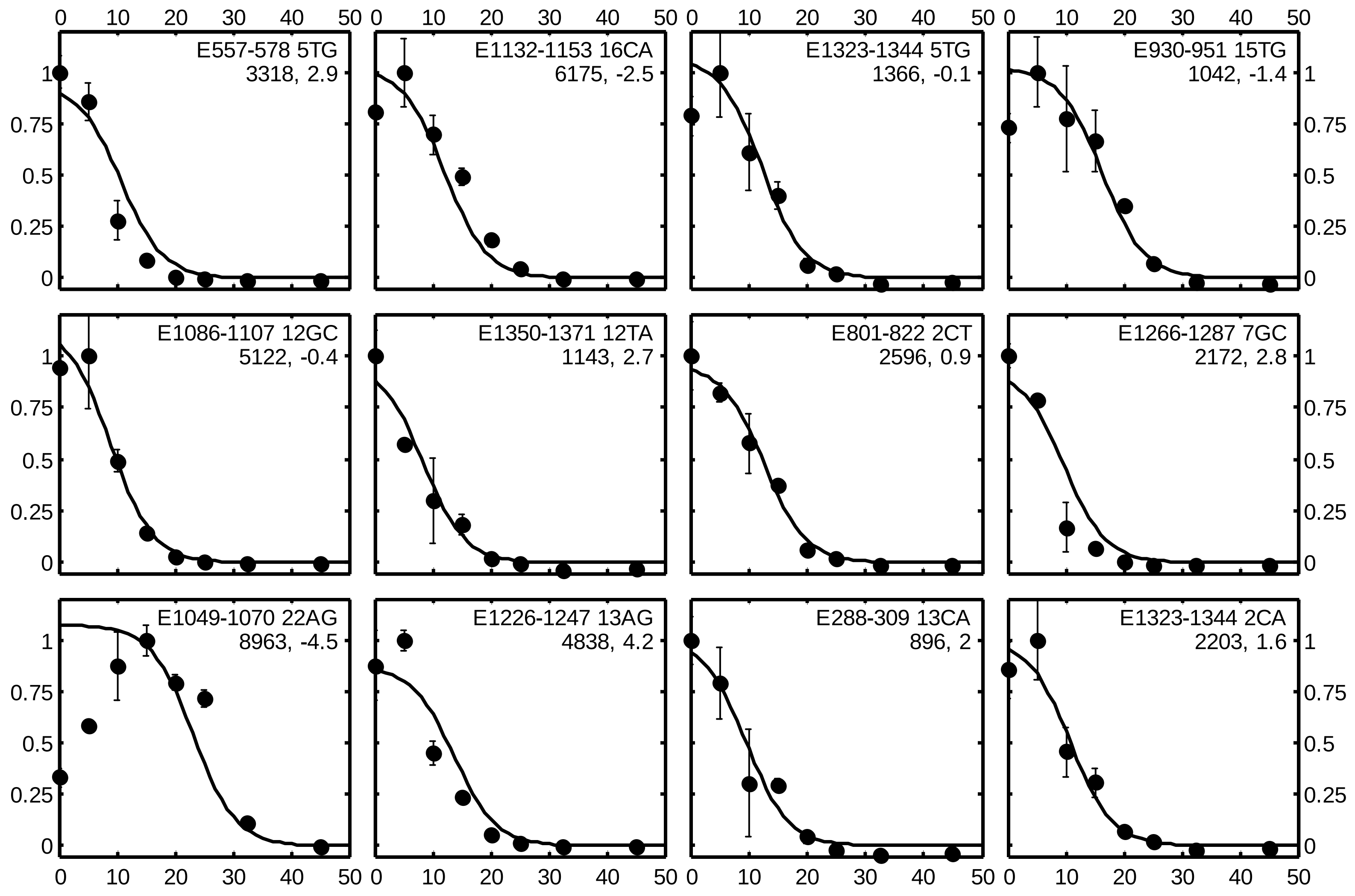
E557-578 5TG
3318, 2.9
E1132-1153 16CA
6175, -2.5
E1323-1344 5TG
1366, -0.1
E930-951 15TG
1042, -1.4
E1086-1107 12GC
5122, -0.4
E1350-1371 12TA
1143, 2.7
E801-822 2CT
2596, 0.9
E1266-1287 7GC
2172, 2.8
E1049-1070 22AG
8963, -4.5
E1226-1247 13AG
4838, 4.2
E288-309 13CA
896, 2
E1323-1344 2CA
2203, 1.6

Probes 93-100:

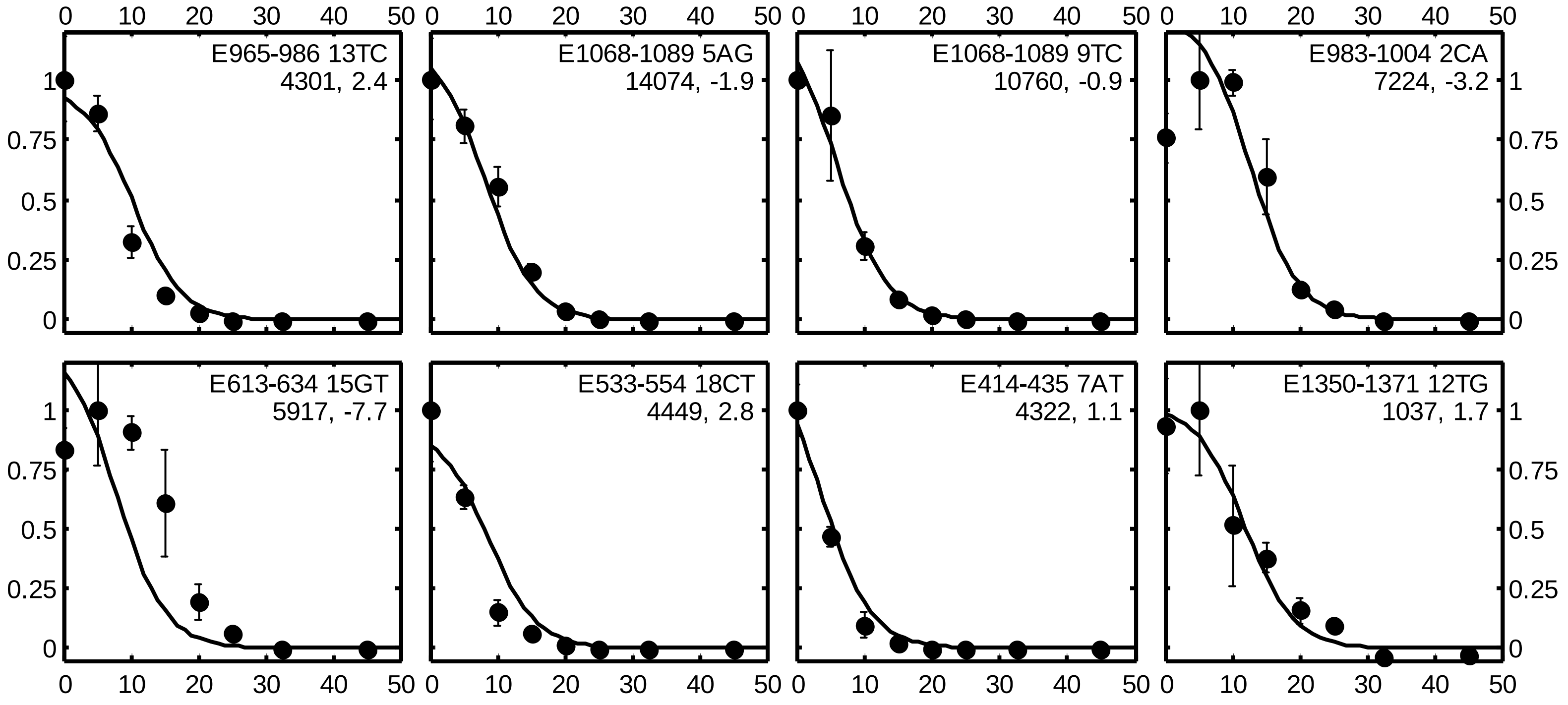

Figure S1D Experimental (circles) and theoretical (curves) formamide dissociation profiles of 100 randomly selected probes from Gap and Insertion sets (Table 2). The labels (upper right corner of each graph) indicate probe name (top), I_{max} (bottom, first number), and $err[FA]_{1/2}$ value (bottom, second number). See main manuscript for the descriptions of probe names and I_{max} and $err[FA]_{1/2}$ values. x-axis, formamide concentration; y-axis, normalized signal intensity; error bars, standard deviations.

Probes 1-8:

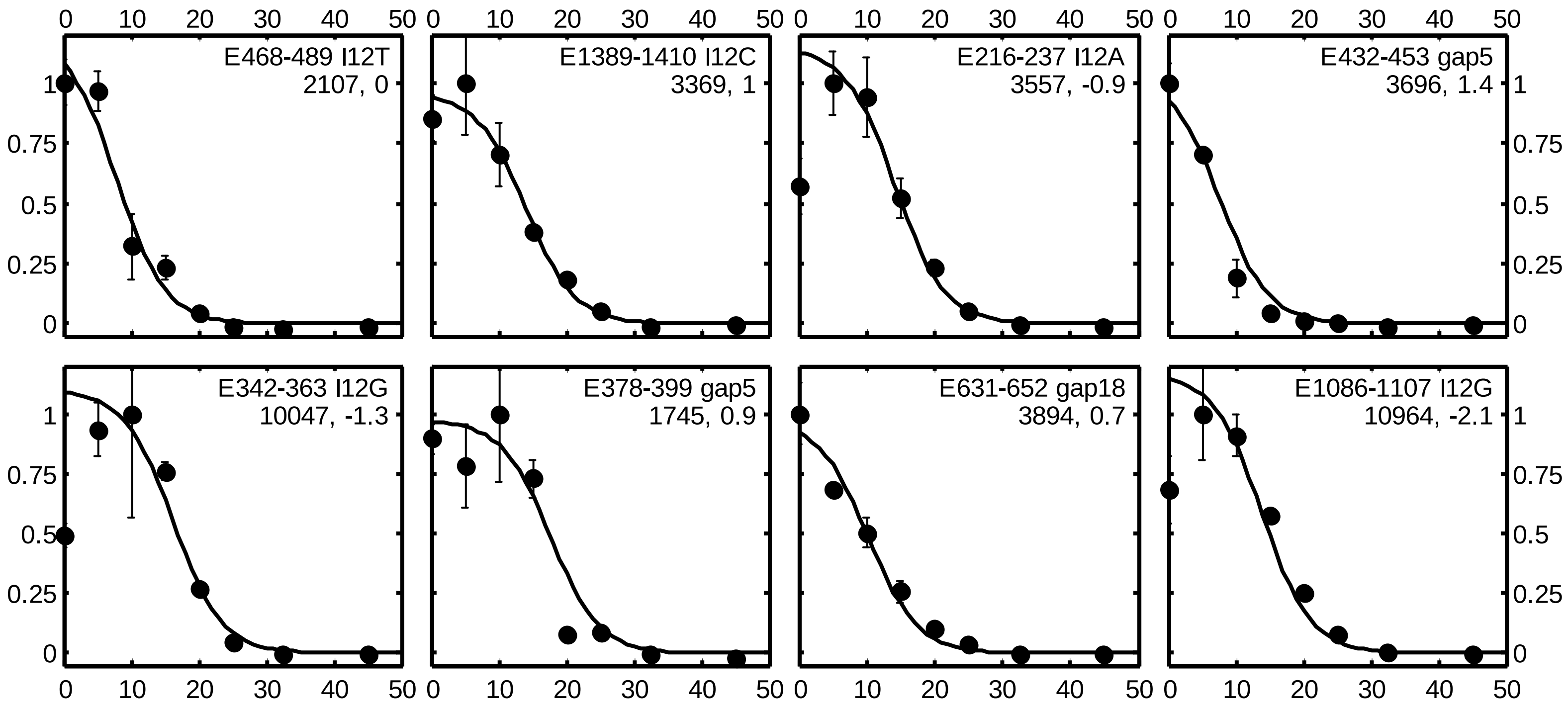

Figure S1D

Probes 9-20:

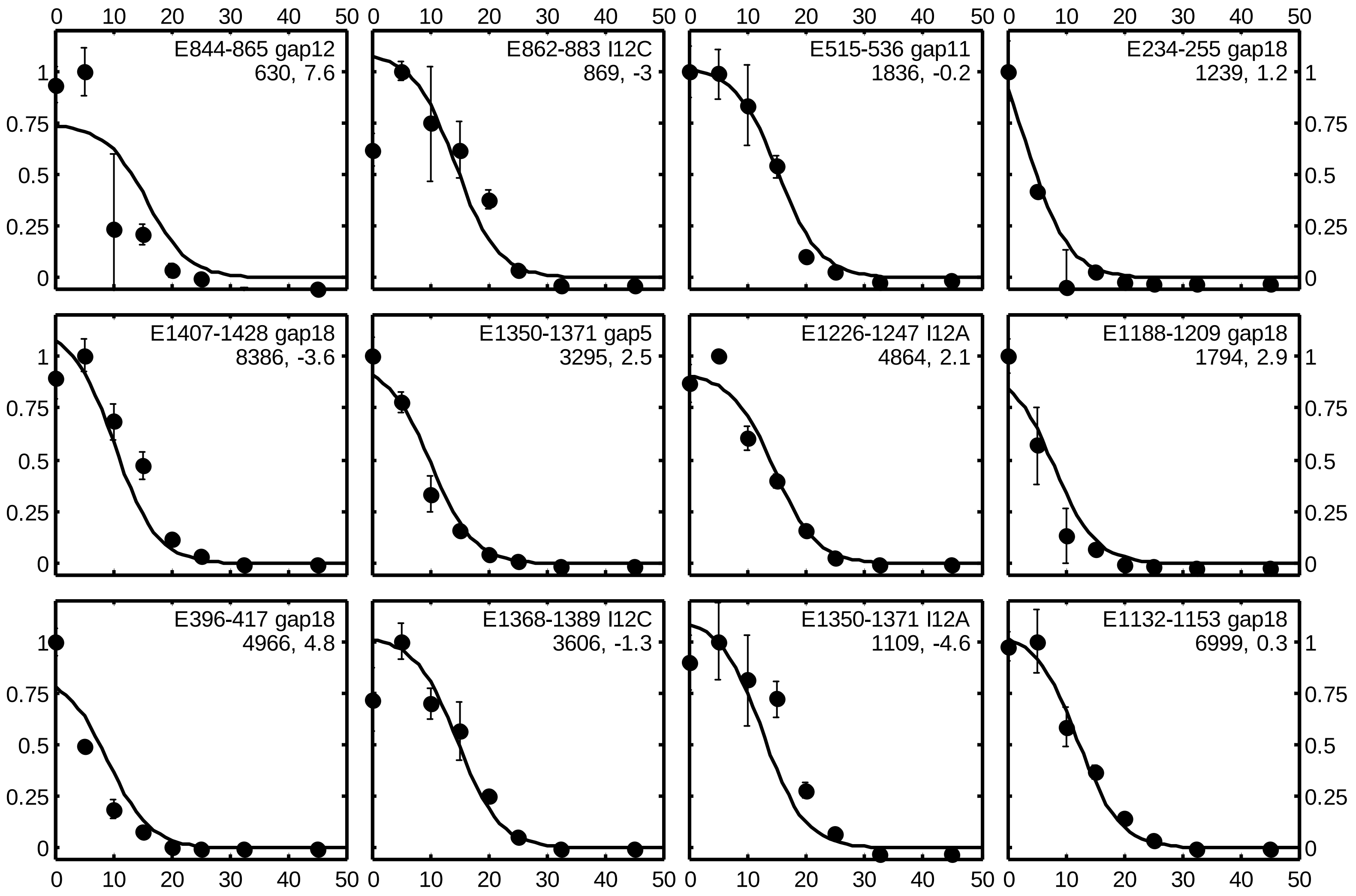

Probes 21-32:

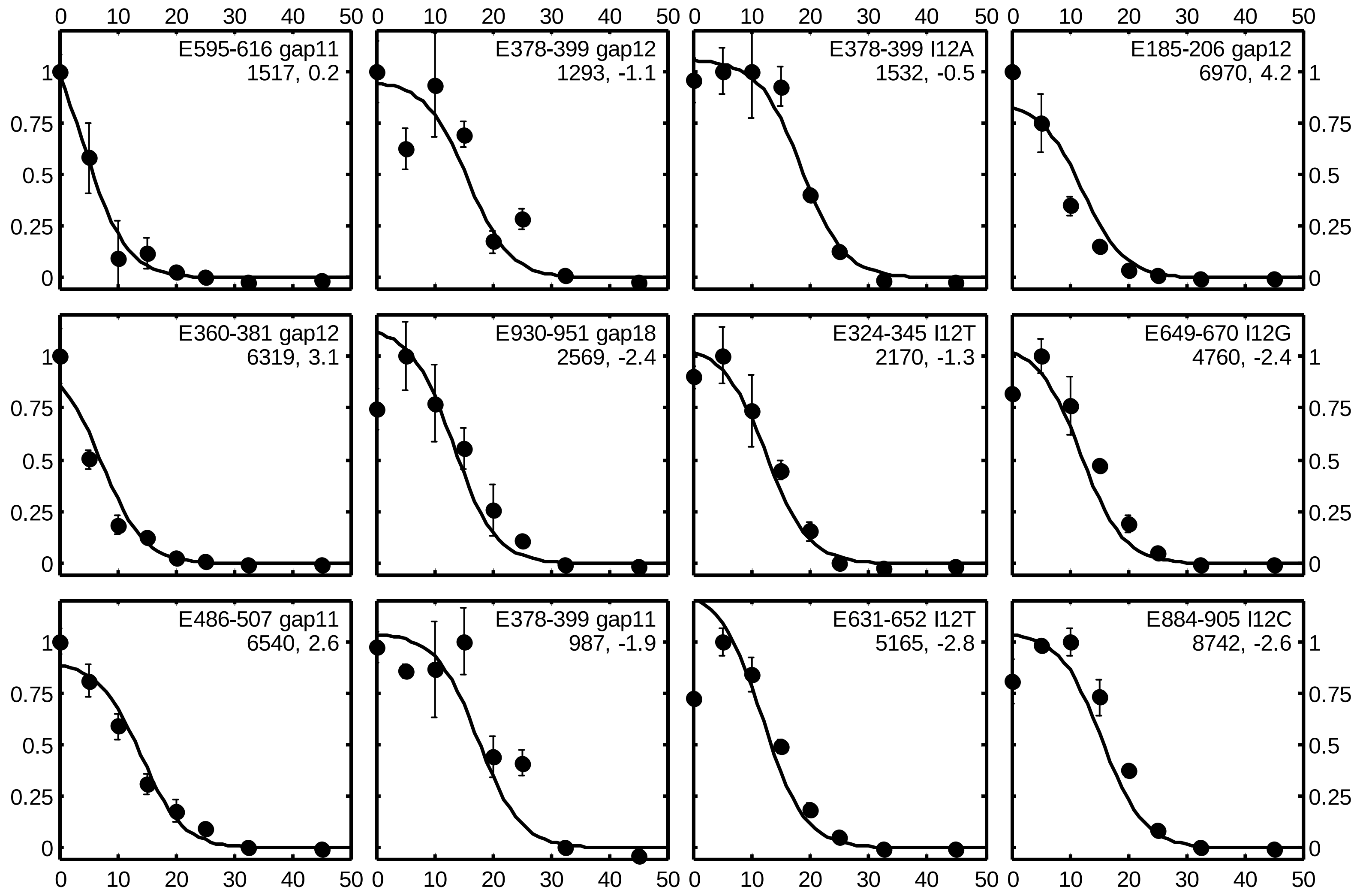
E595-616 gap11
1517, 0.2
E378-399 gap12
1293, -1.1
E378-399 I12A
1532, -0.5
E185-206 gap12
6970, 4.2
E360-381 gap12
6319, 3.1
E930-951 gap18
2569, -2.4
E324-345 I12T
2170, -1.3
E649-670 I12G
4760, -2.4
E486-507 gap11
6540, 2.6
E378-399 gap11
987, -1.9
E631-652 I12T
5165, -2.8
E884-905 I12C
8742, -2.6
0
10
20
30
40
50
0
0.25
0.5
0.75
1

Figure S1D

Probes 33-44:

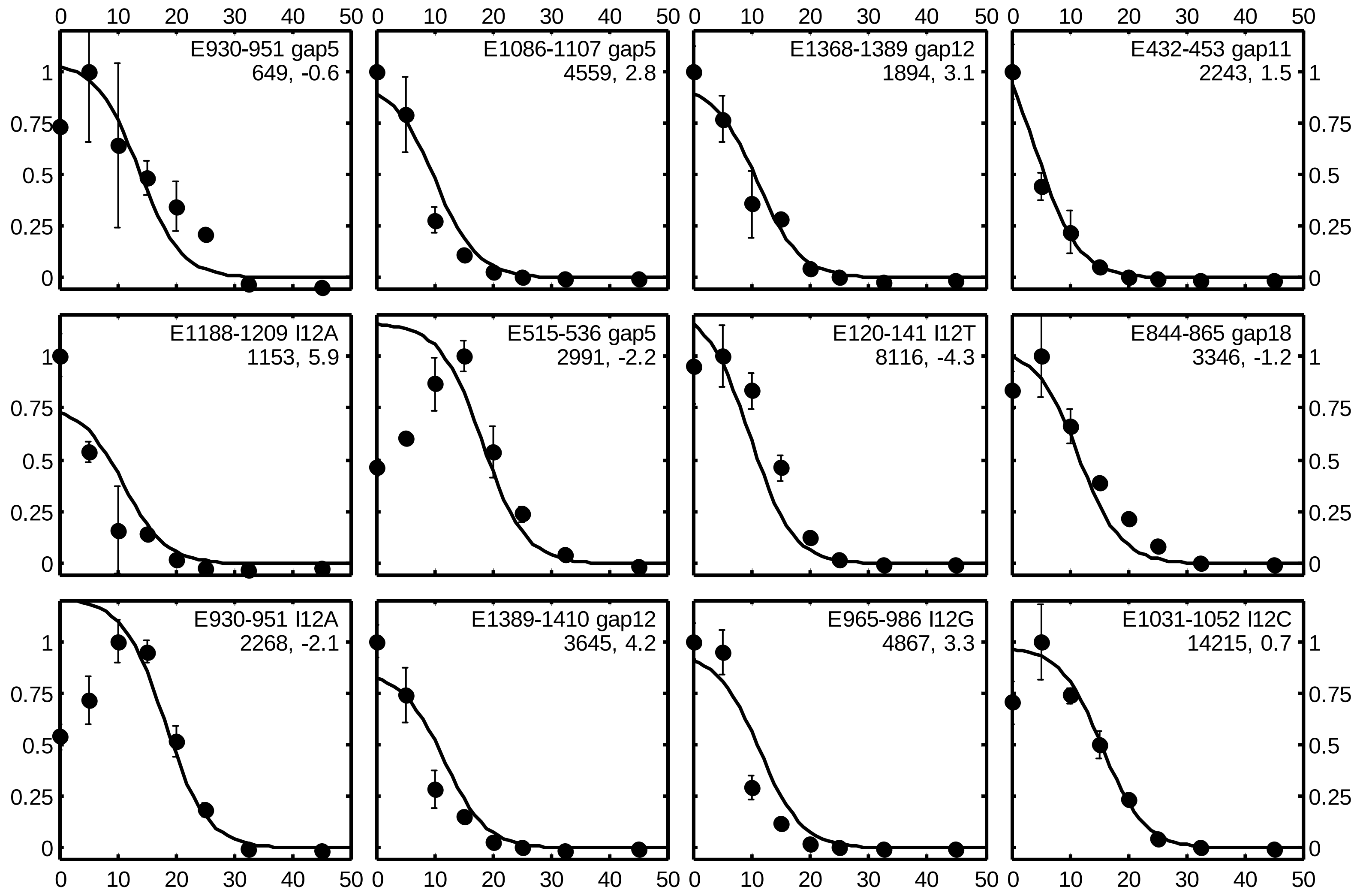

Probes 45-56:

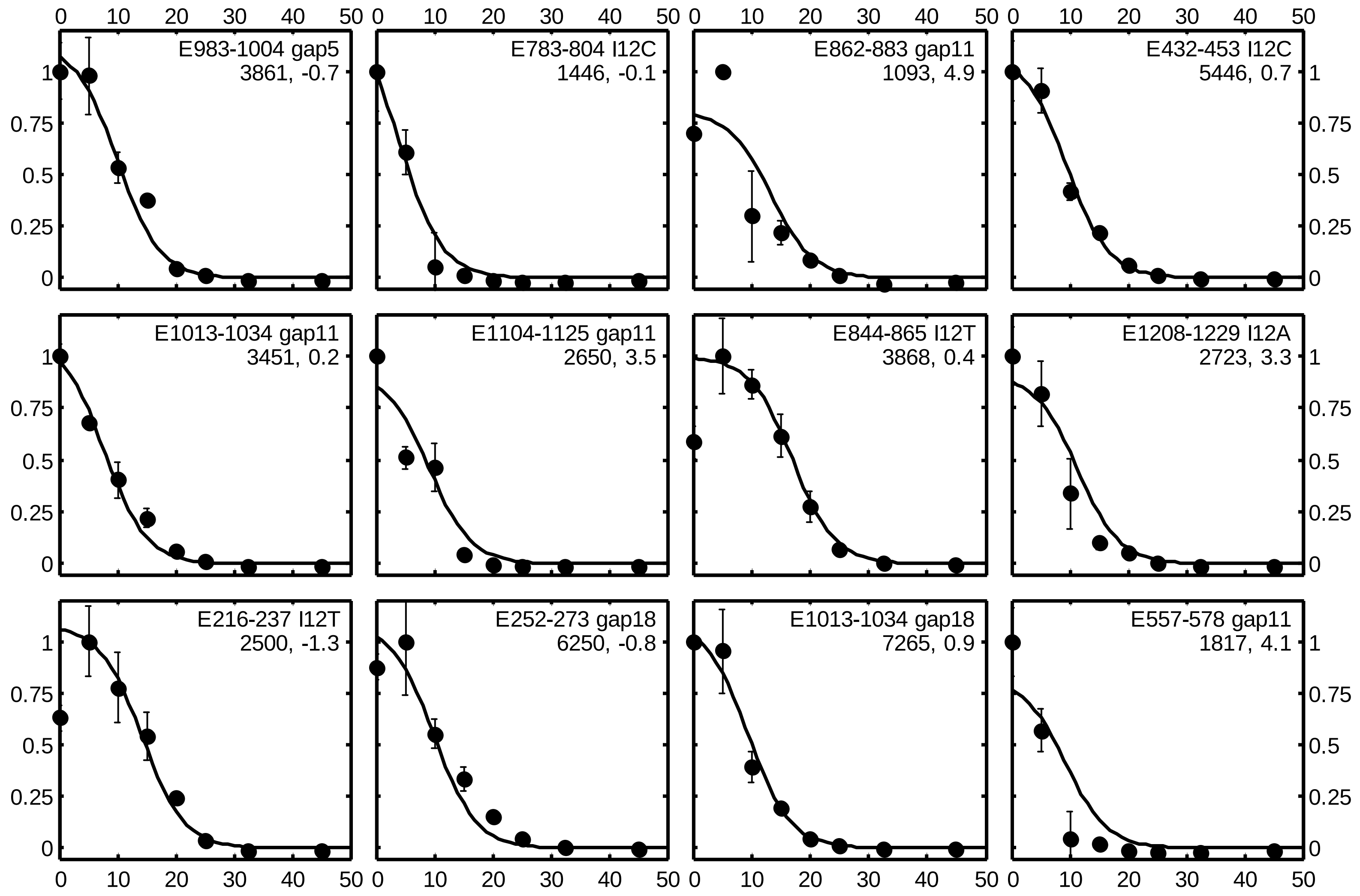
E983-1004 gap5
3861, -0.7
E783-804 I12C
1446, -0.1
E862-883 gap11
1093, 4.9
E432-453 I12C
5446, 0.7
E1013-1034 gap11
3451, 0.2
E1104-1125 gap11
2650, 3.5
E844-865 I12T
3868, 0.4
E1208-1229 I12A
2723, 3.3
E216-237 I12T
2500, -1.3
E252-273 gap18
6250, -0.8
E1013-1034 gap18
7265, 0.9
E557-578 gap11
1817, 4.1
0 10 20 30 40 50
0 0.25 0.5 0.75 1

Figure S1D

Probes 57-68:

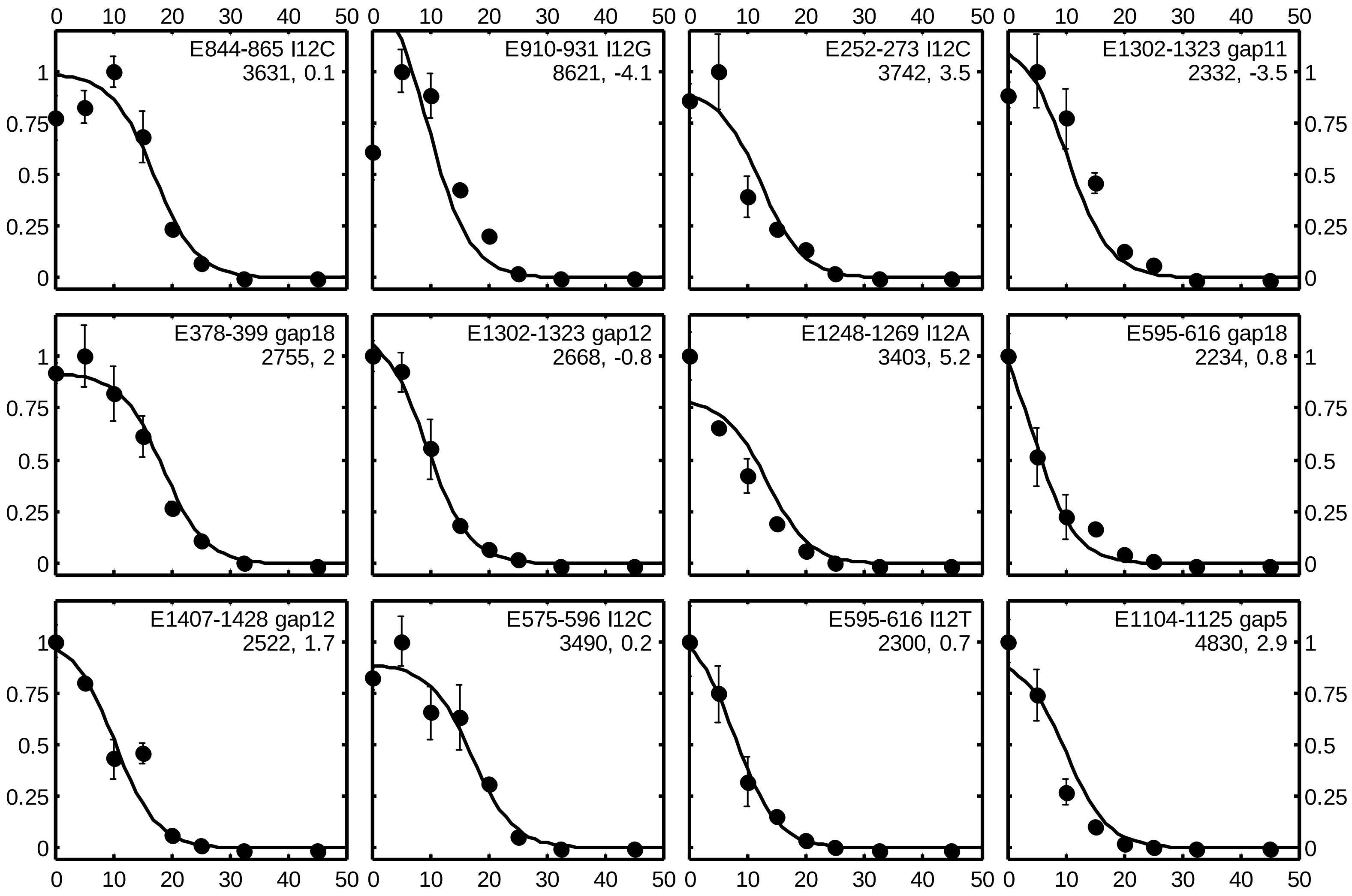

Probes 69-80:

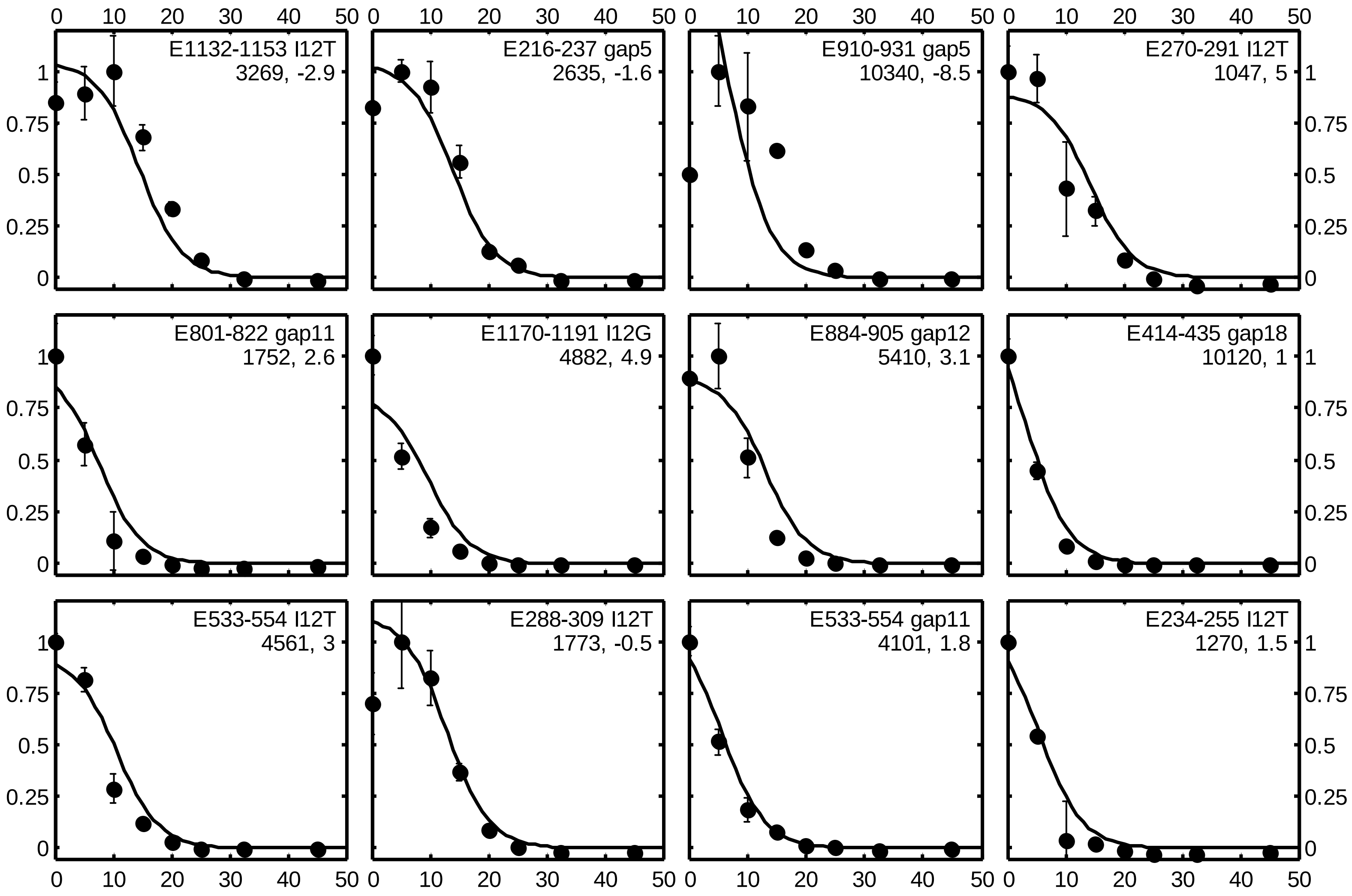
E1132-1153 I12T
3269, -2.9
E216-237 gap5
2635, -1.6
E910-931 gap5
10340, -8.5
E270-291 I12T
1047, 5
E801-822 gap11
1752, 2.6
E1170-1191 I12G
4882, 4.9
E884-905 gap12
5410, 3.1
E414-435 gap18
10120, 1
E533-554 I12T
4561, 3
E288-309 I12T
1773, -0.5
E533-554 gap11
4101, 1.8
E234-255 I12T
1270, 1.5

Figure S1D

Probes 81-92:

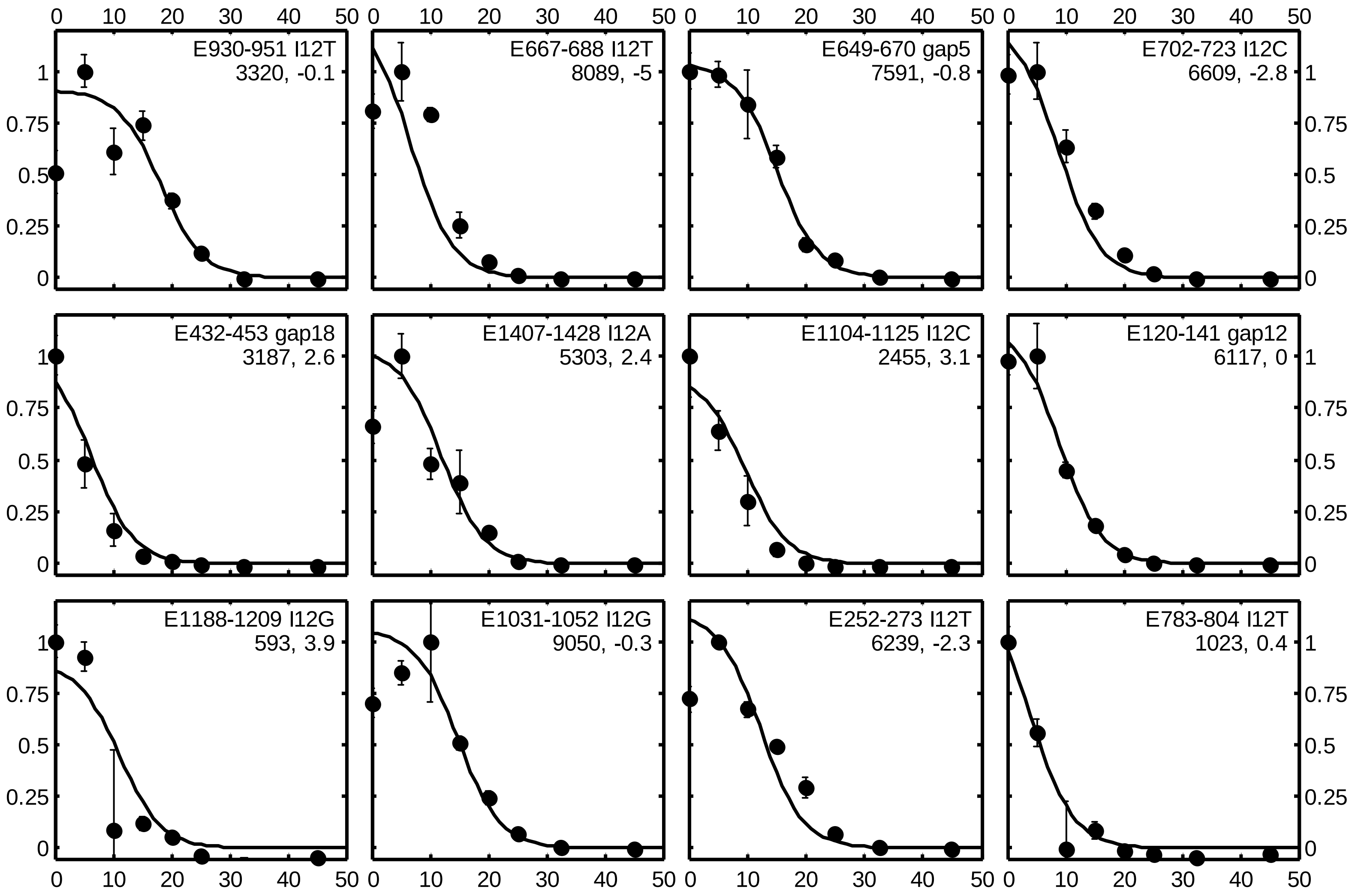

Figure S1D

Probes 93-100:

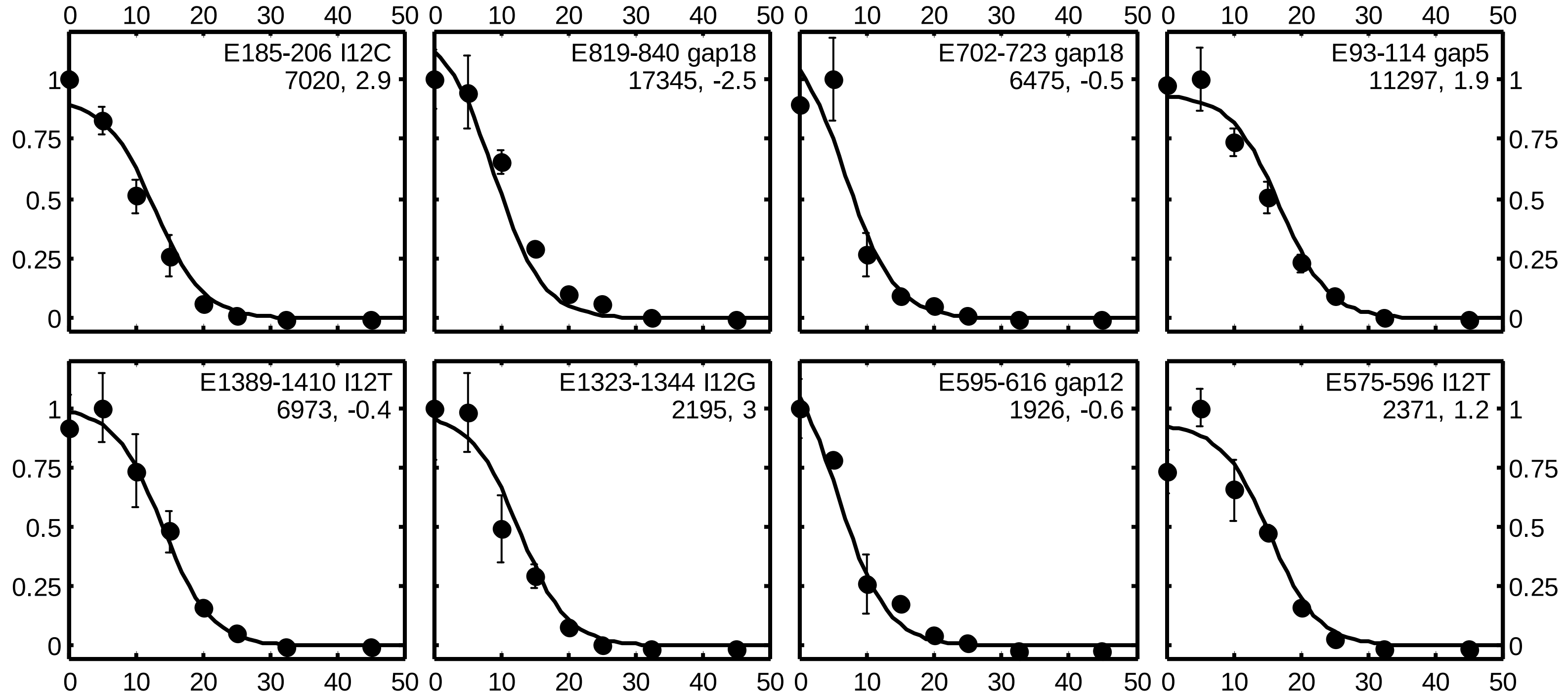

Figure S1E. Experimental (circles) and theoretical (curves) formamide dissociation profiles of 100 randomly selected probes from TwoM set (Table 2). The labels (upper right corner of each graph) indicate probe name (top), I_{max} (bottom, first number), and err$[FA]_{1/2}$ value (bottom, second number). See main manuscript for the descriptions of probe names and I_{max} and err$[FA]_{1/2}$ values. x-axis, formamide concentration; y-axis, normalized signal intensity; error bars, standard deviations.

Probes 1-8:

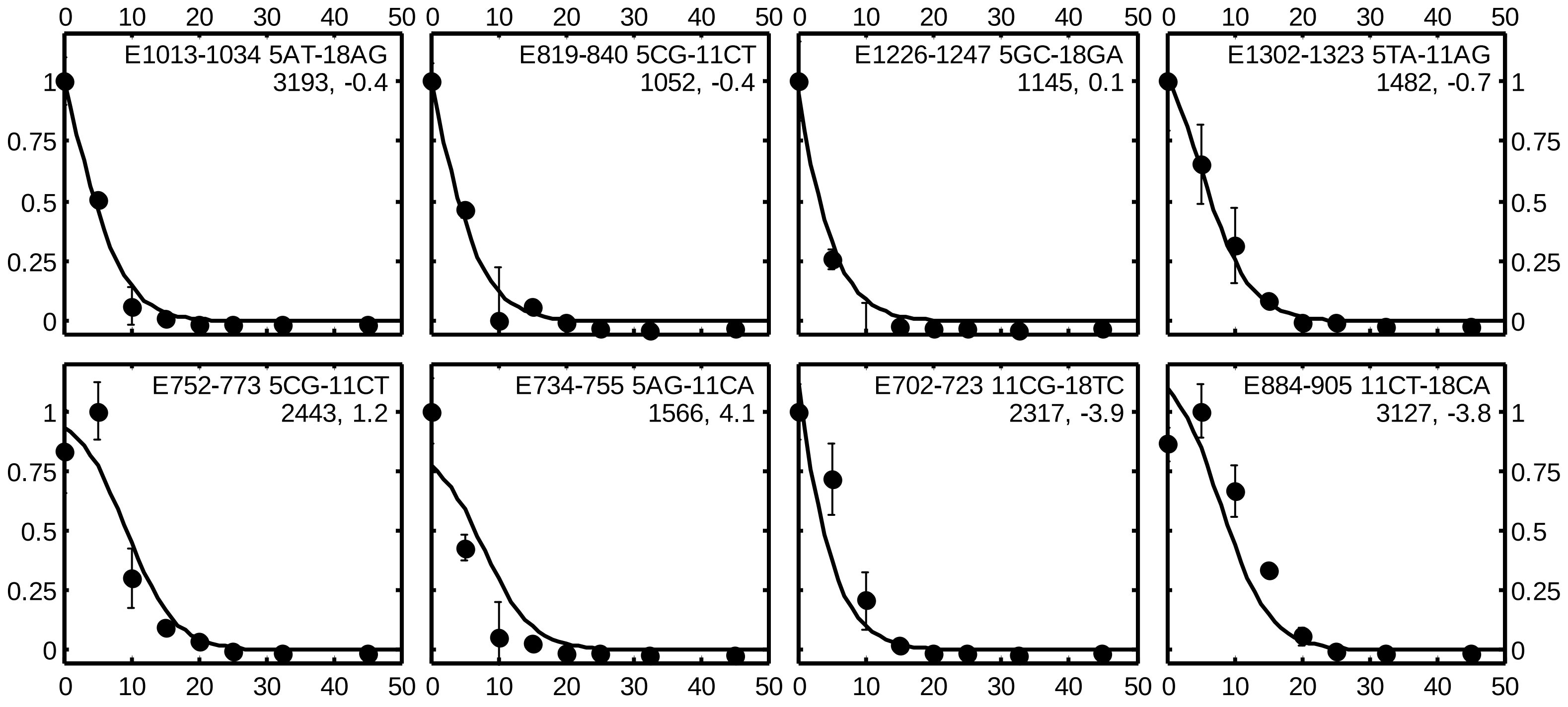

Probes 9-20:

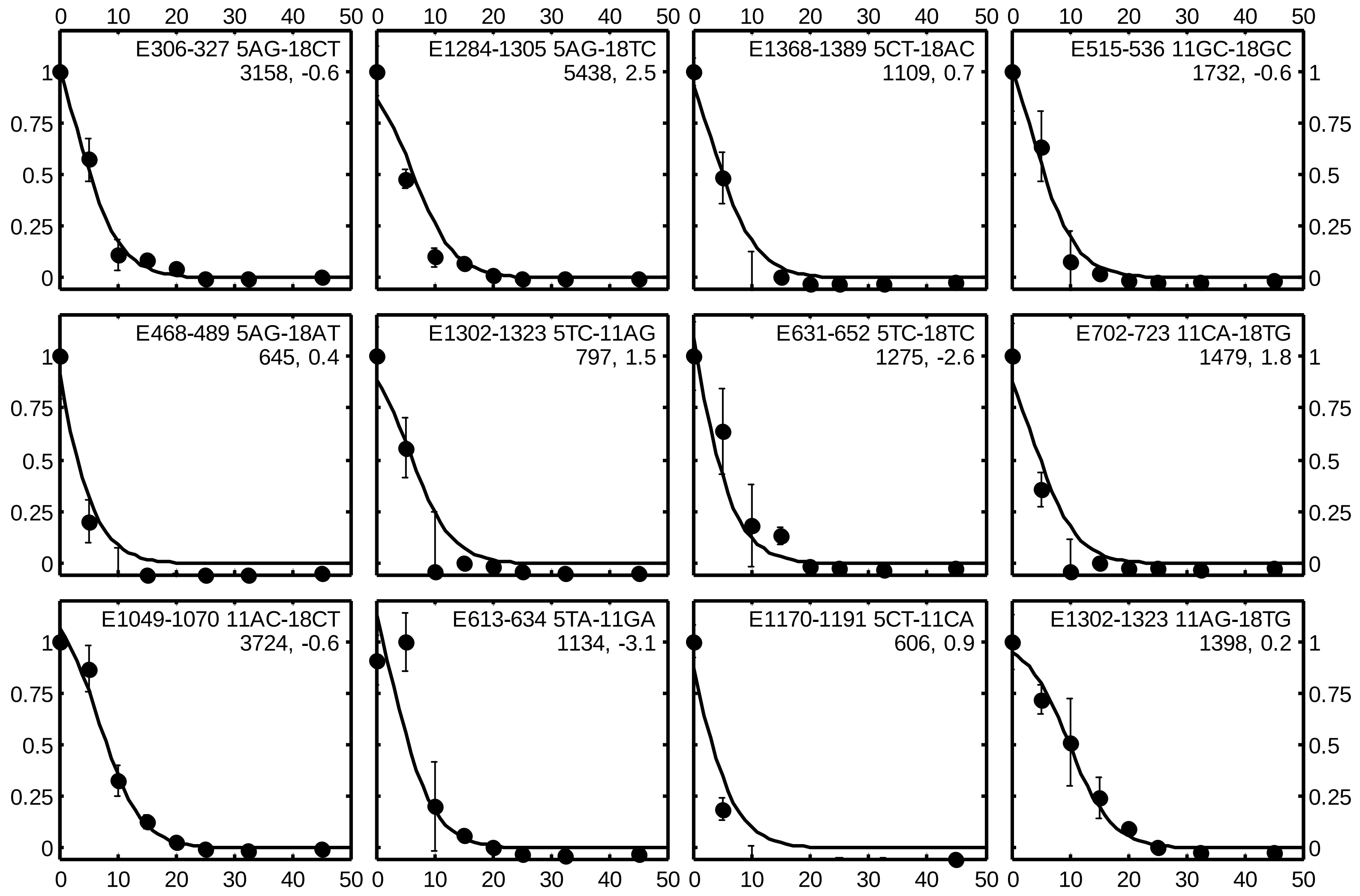
E306-327 5AG-18CT
3158, -0.6
E1284-1305 5AG-18TC
5438, 2.5
E1368-1389 5CT-18AC
1109, 0.7
E515-536 11GC-18GC
1732, -0.6
E468-489 5AG-18AT
645, 0.4
E1302-1323 5TC-11AG
797, 1.5
E631-652 5TC-18TC
1275, -2.6
E702-723 11CA-18TG
1479, 1.8
E1049-1070 11AC-18CT
3724, -0.6
E613-634 5TA-11GA
1134, -3.1
E1170-1191 5CT-11CA
606, 0.9
E1302-1323 11AG-18TG
1398, 0.2

Probes 21-32:

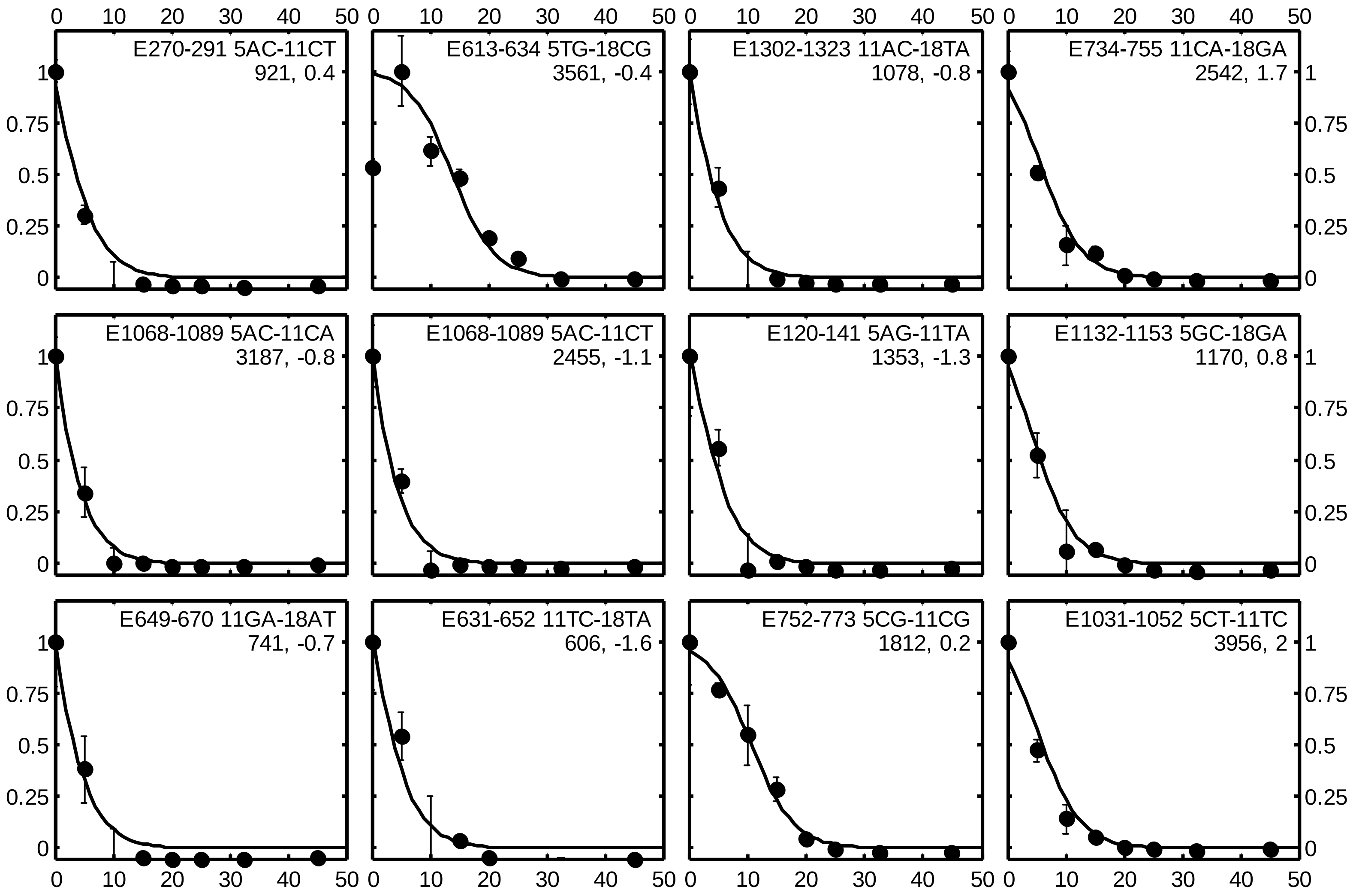
E270-291 5AC-11CT
921, 0.4
E613-634 5TG-18CG
3561, -0.4
E1302-1323 11AC-18TA
1078, -0.8
E734-755 11CA-18GA
2542, 1.7
E1068-1089 5AC-11CA
3187, -0.8
E1068-1089 5AC-11CT
2455, -1.1
E120-141 5AG-11TA
1353, -1.3
E1132-1153 5GC-18GA
1170, 0.8
E649-670 11GA-18AT
741, -0.7
E631-652 11TC-18TA
606, -1.6
E752-773 5CG-11CG
1812, 0.2
E1031-1052 5CT-11TC
3956, 2
0
10
20
30
40
50
0
0.25
0.5
0.75
1

Probes 33-44:

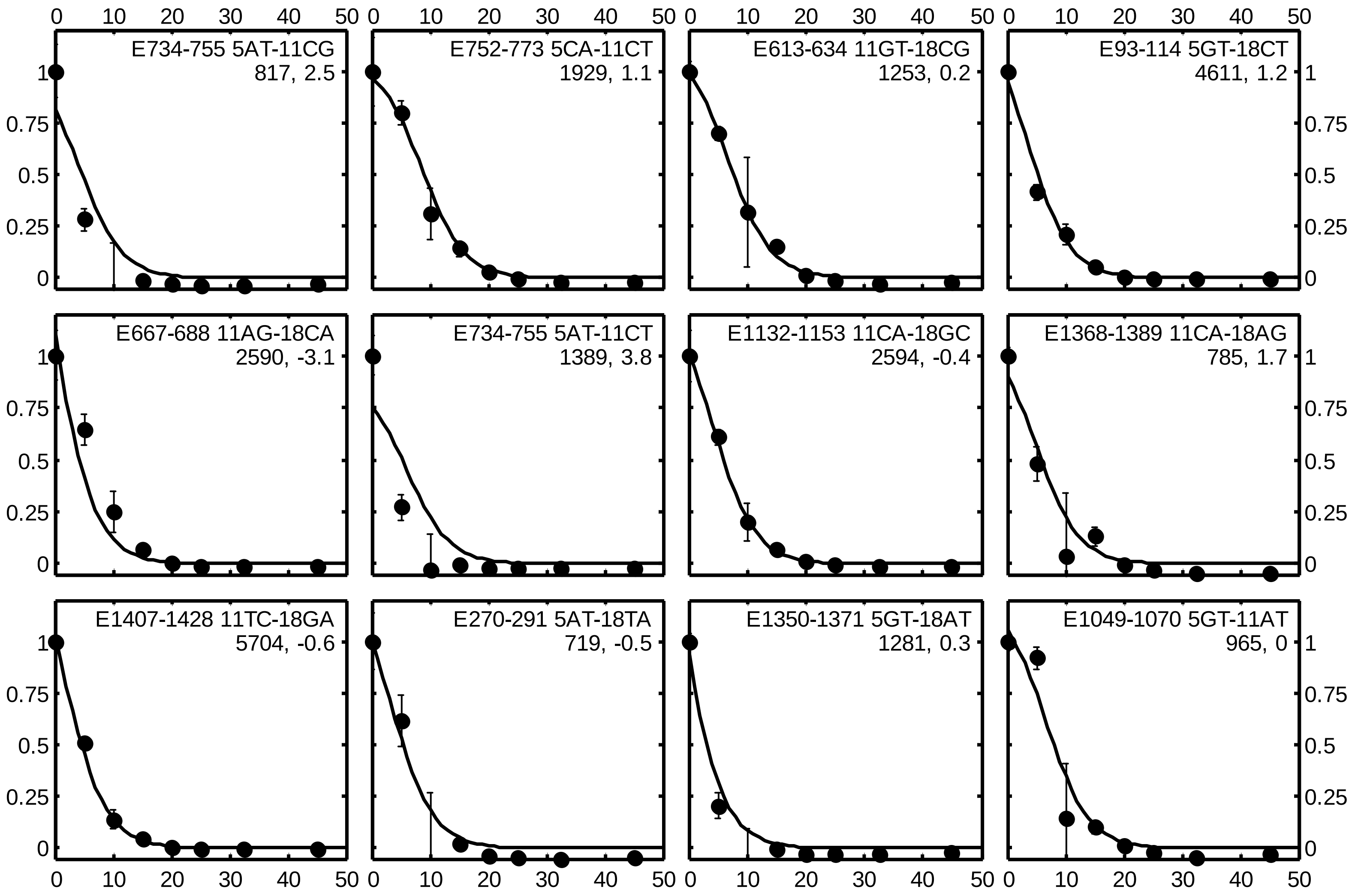
E734-755 5AT-11CG
817, 2.5
E752-773 5CA-11CT
1929, 1.1
E613-634 11GT-18CG
1253, 0.2
E93-114 5GT-18CT
4611, 1.2
E667-688 11AG-18CA
2590, -3.1
E734-755 5AT-11CT
1389, 3.8
E1132-1153 11CA-18GC
2594, -0.4
E1368-1389 11CA-18AG
785, 1.7
E1407-1428 11TC-18GA
5704, -0.6
E270-291 5AT-18TA
719, -0.5
E1350-1371 5GT-18AT
1281, 0.3
E1049-1070 5GT-11AT
965, 0

Probes 45-56:

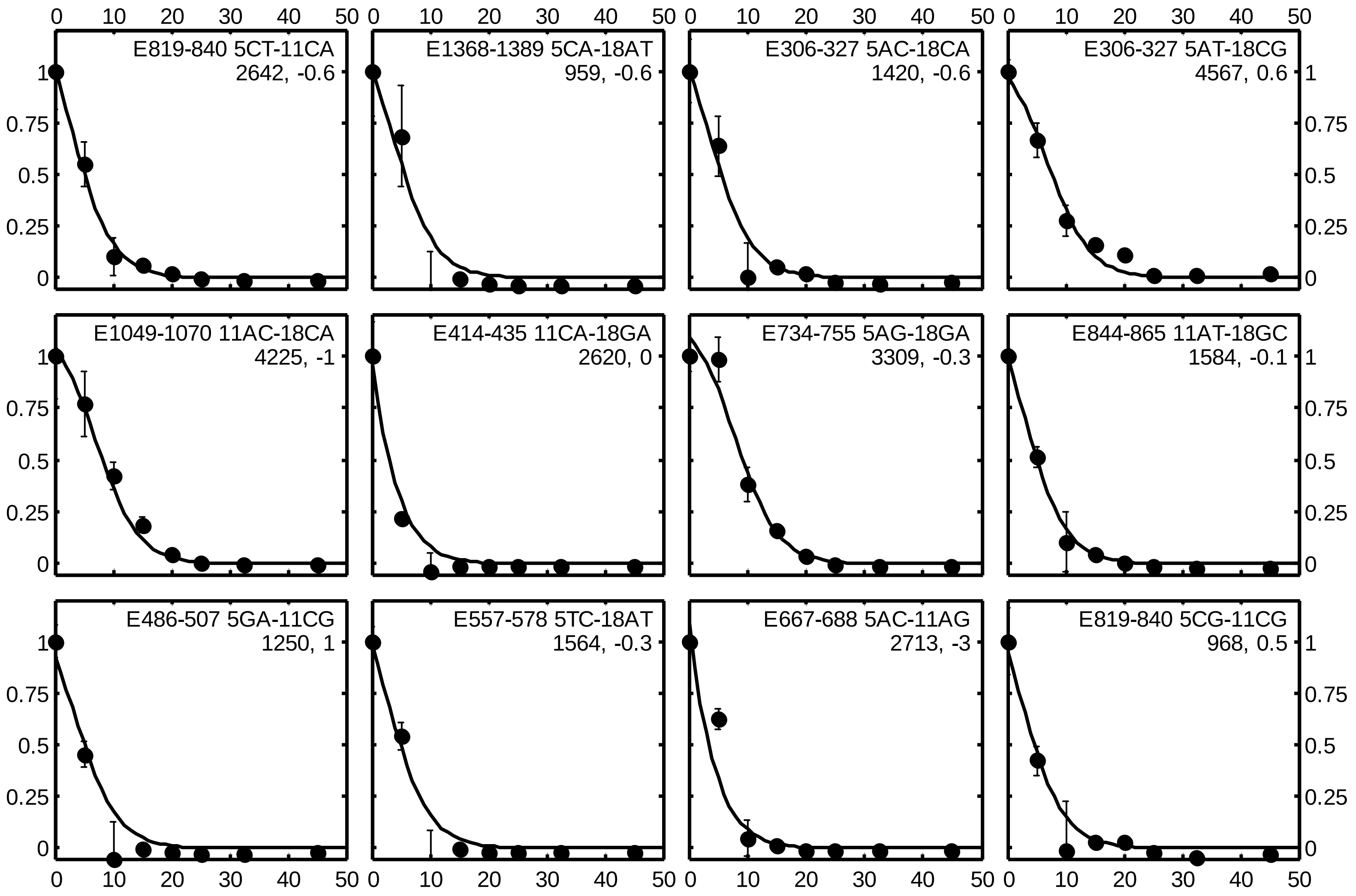
E819-840 5CT-11CA
2642, -0.6
E1368-1389 5CA-18AT
959, -0.6
E306-327 5AC-18CA
1420, -0.6
E306-327 5AT-18CG
4567, 0.6
E1049-1070 11AC-18CA
4225, -1
E414-435 11CA-18GA
2620, 0
E734-755 5AG-18GA
3309, -0.3
E844-865 11AT-18GC
1584, -0.1
E486-507 5GA-11CG
1250, 1
E557-578 5TC-18AT
1564, -0.3
E667-688 5AC-11AG
2713, -3
E819-840 5CG-11CG
968, 0.5
0
10
20
30
40
50
0
0.25
0.5
0.75
1

Probes 57-68:

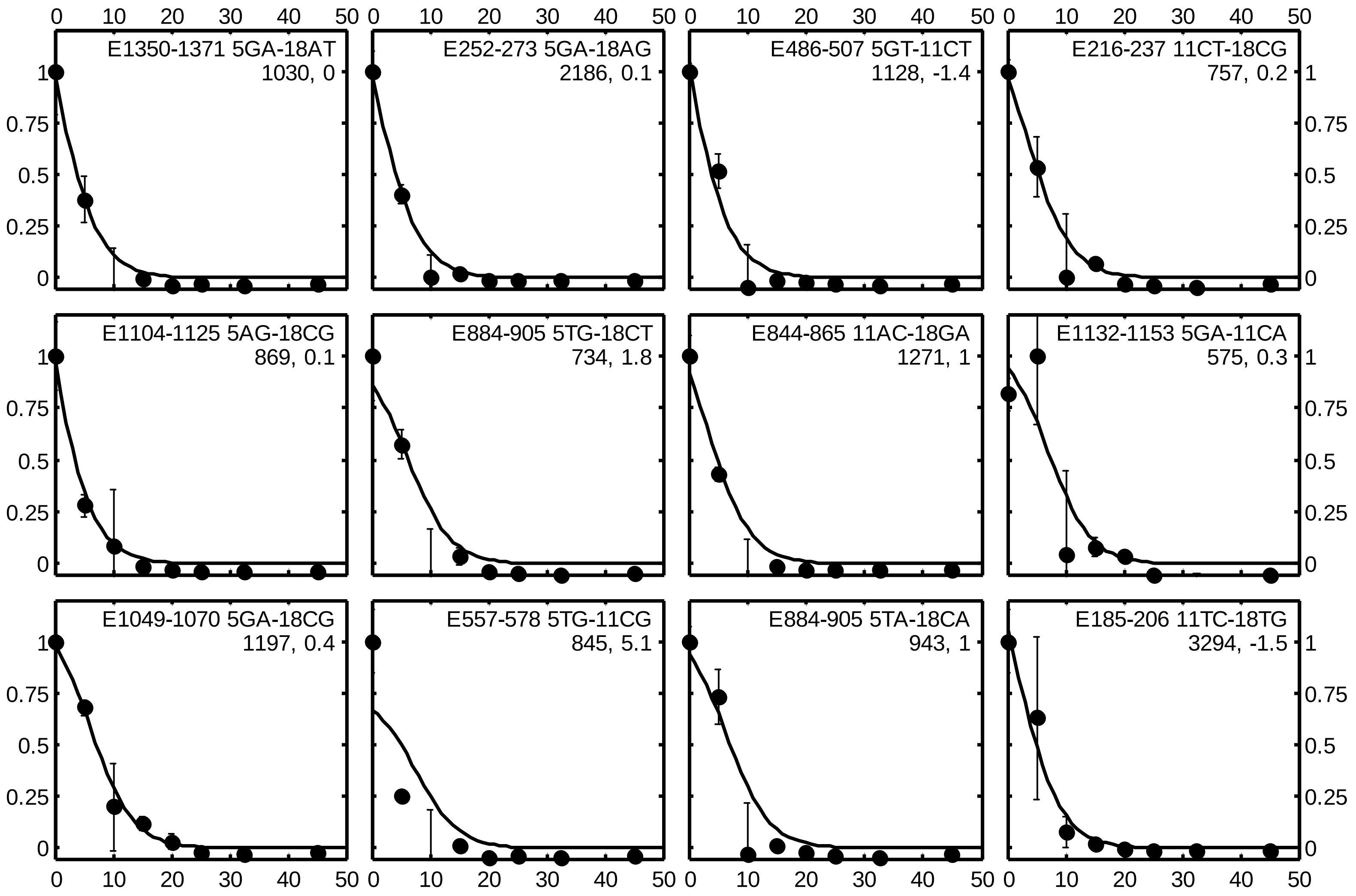
E1350-1371 5GA-18AT
1030, 0
E252-273 5GA-18AG
2186, 0.1
E486-507 5GT-11CT
1128, -1.4
E216-237 11CT-18CG
757, 0.2
E1104-1125 5AG-18CG
869, 0.1
E884-905 5TG-18CT
734, 1.8
E844-865 11AC-18GA
1271, 1
E1132-1153 5GA-11CA
575, 0.3
E1049-1070 5GA-18CG
1197, 0.4
E557-578 5TG-11CG
845, 5.1
E884-905 5TA-18CA
943, 1
E185-206 11TC-18TG
3294, -1.5

Figure S1E

Probes 69-80:

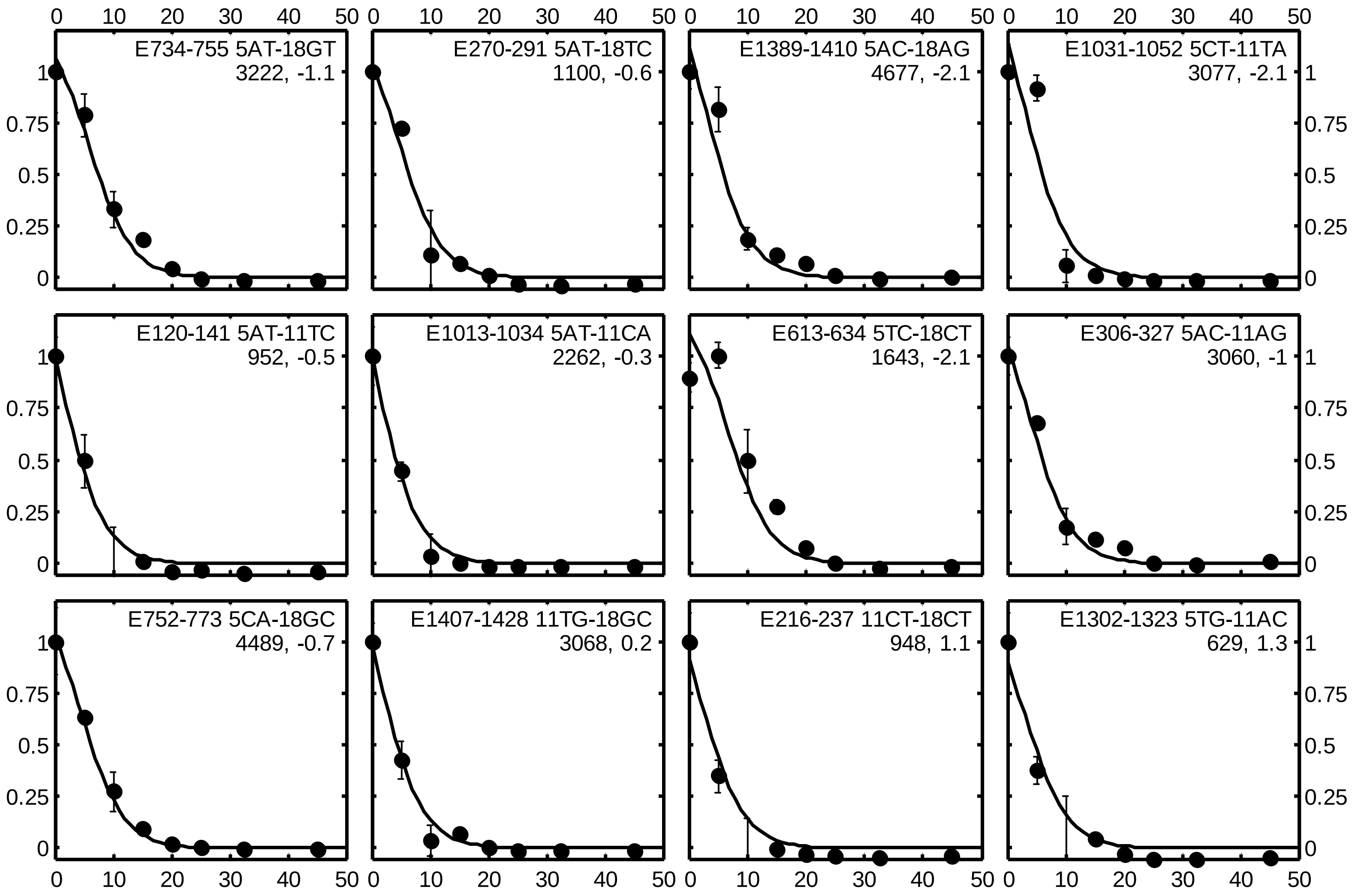

Probes 81-92:

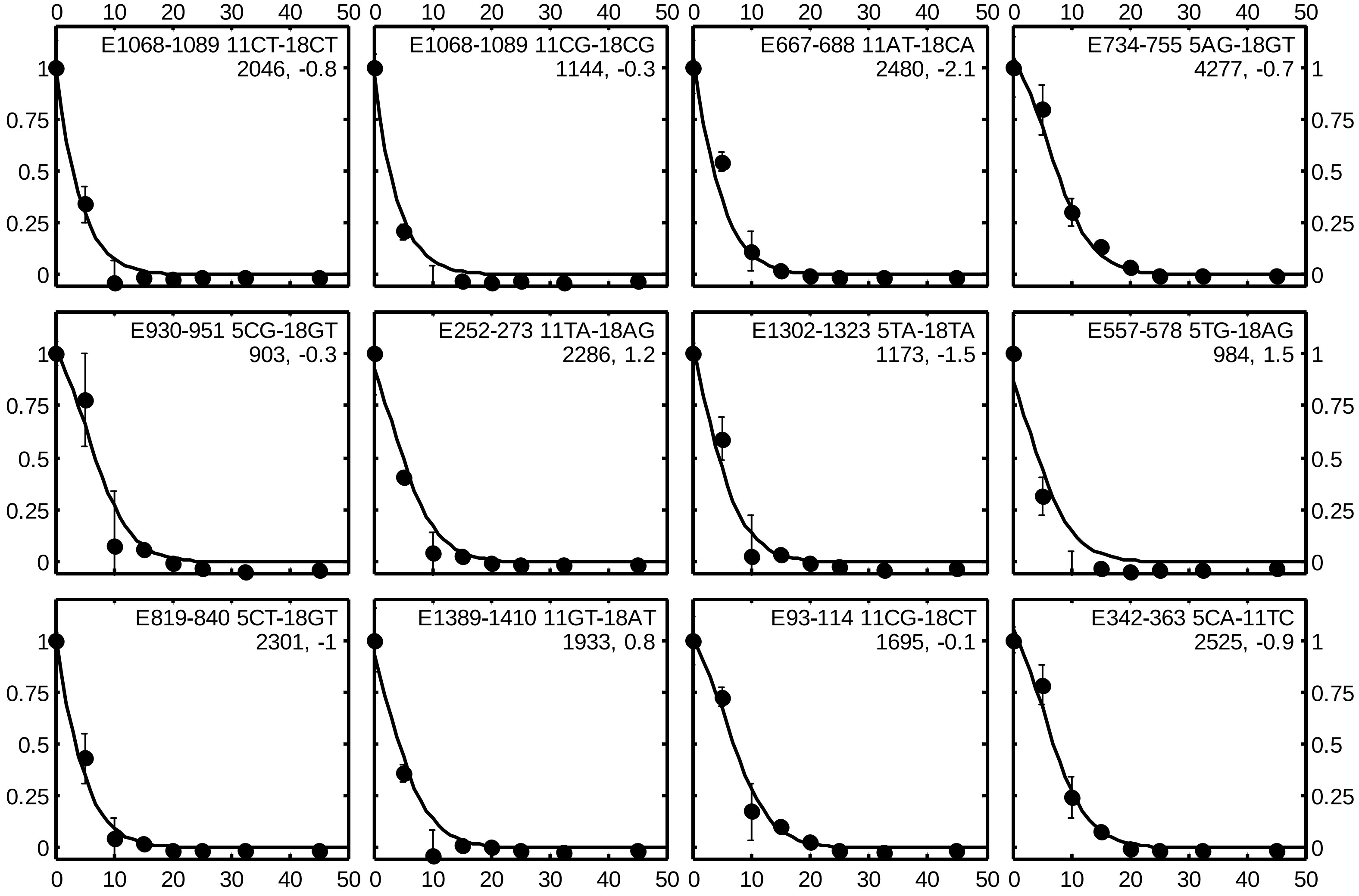
E1068-1089 11CT-18CT
2046, -0.8
E1068-1089 11CG-18CG
1144, -0.3
E667-688 11AT-18CA
2480, -2.1
E734-755 5AG-18GT
4277, -0.7
E930-951 5CG-18GT
903, -0.3
E252-273 11TA-18AG
2286, 1.2
E1302-1323 5TA-18TA
1173, -1.5
E557-578 5TG-18AG
984, 1.5
E819-840 5CT-18GT
2301, -1
E1389-1410 11GT-18AT
1933, 0.8
E93-114 11CG-18CT
1695, -0.1
E342-363 5CA-11TC
2525, -0.9

Probes 93-100:

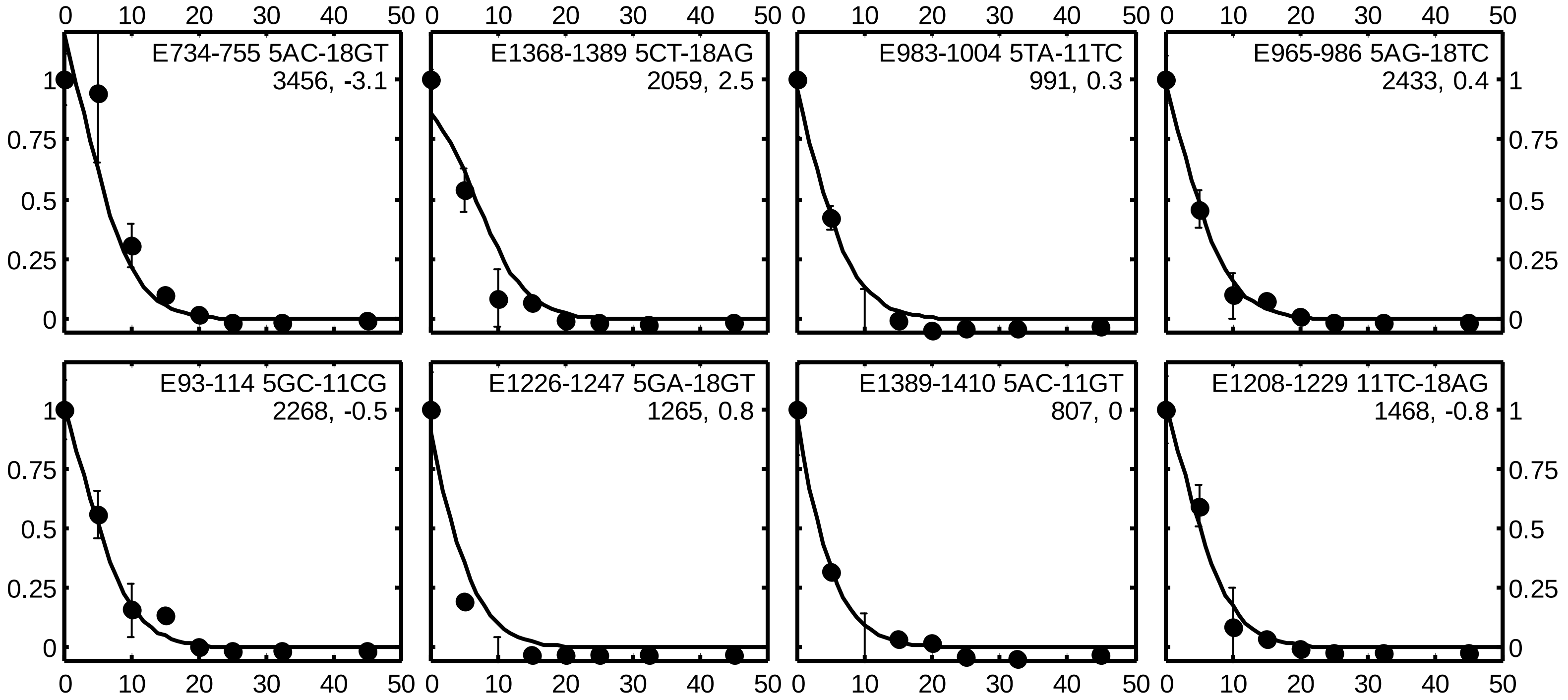

Figure S1F. Experimental (circles) and theoretical (curves) formamide dissociation profiles of 100 randomly selected probes from Tandem set (Table 2). The labels (upper right corner of each graph) indicate probe name (top), I_{max} (bottom, first number), and err[FA]$_{1/2}$ value (bottom, second number). See main manuscript for the descriptions of probe names and I_{max} and err[FA]$_{1/2}$ values. x-axis, formamide concentration; y-axis, normalized signal intensity; error bars, standard deviations.

Probes 1-8:

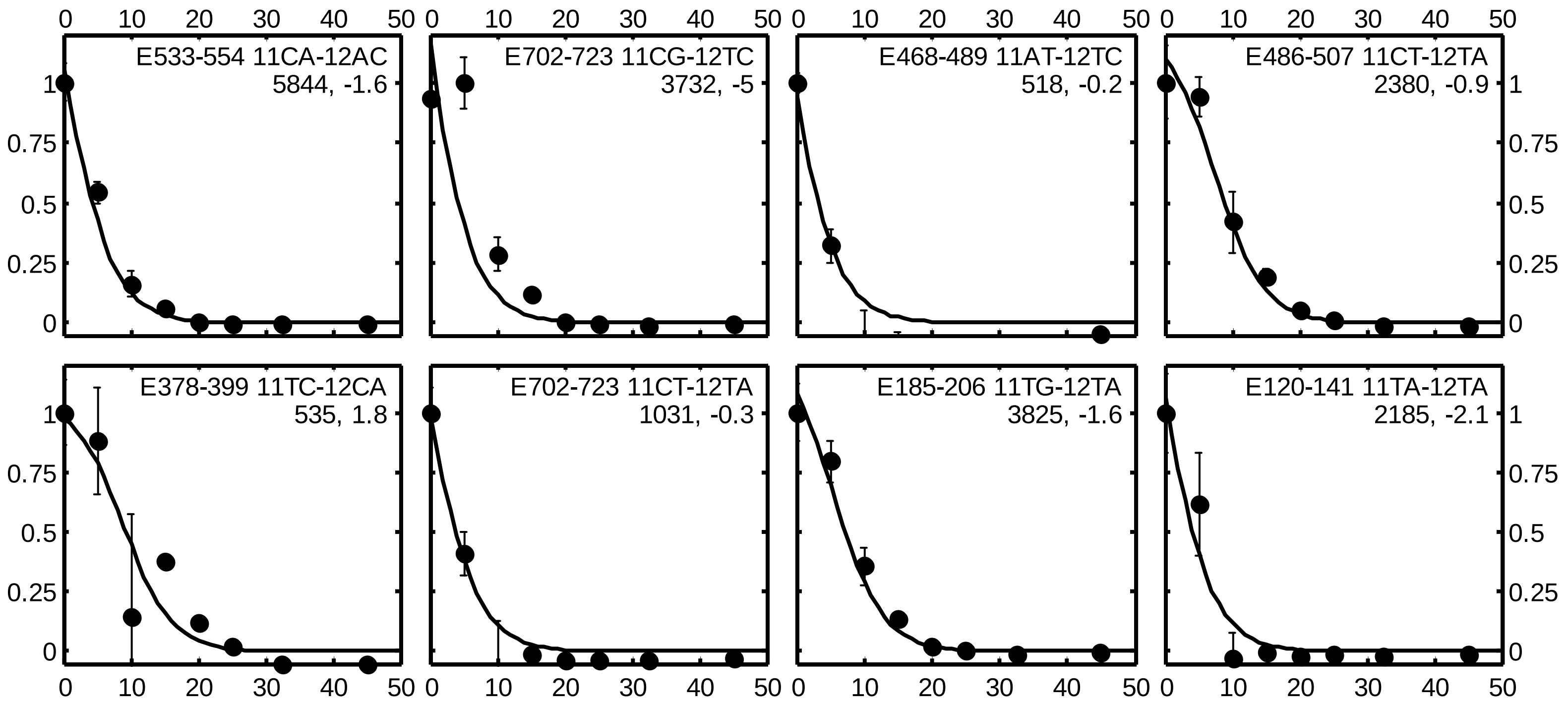

Probes 9-20:

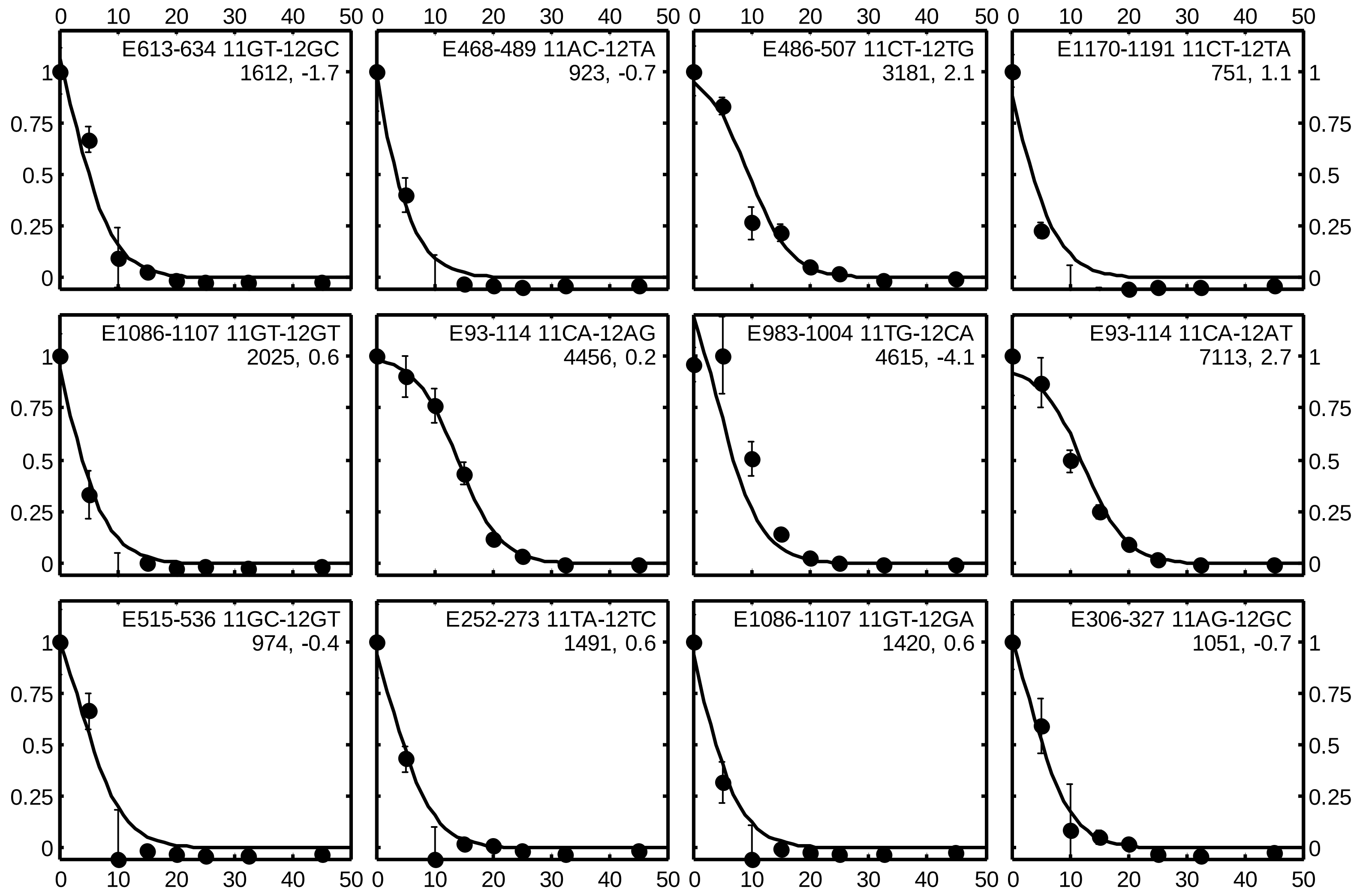
E613-634 11GT-12GC
1612, -1.7
E468-489 11AC-12TA
923, -0.7
E486-507 11CT-12TG
3181, 2.1
E1170-1191 11CT-12TA
751, 1.1
E1086-1107 11GT-12GT
2025, 0.6
E93-114 11CA-12AG
4456, 0.2
E983-1004 11TG-12CA
4615, -4.1
E93-114 11CA-12AT
7113, 2.7
E515-536 11GC-12GT
974, -0.4
E252-273 11TA-12TC
1491, 0.6
E1086-1107 11GT-12GA
1420, 0.6
E306-327 11AG-12GC
1051, -0.7
0 10 20 30 40 50
1 0.75 0.5 0.25 0

Probes 21-32:

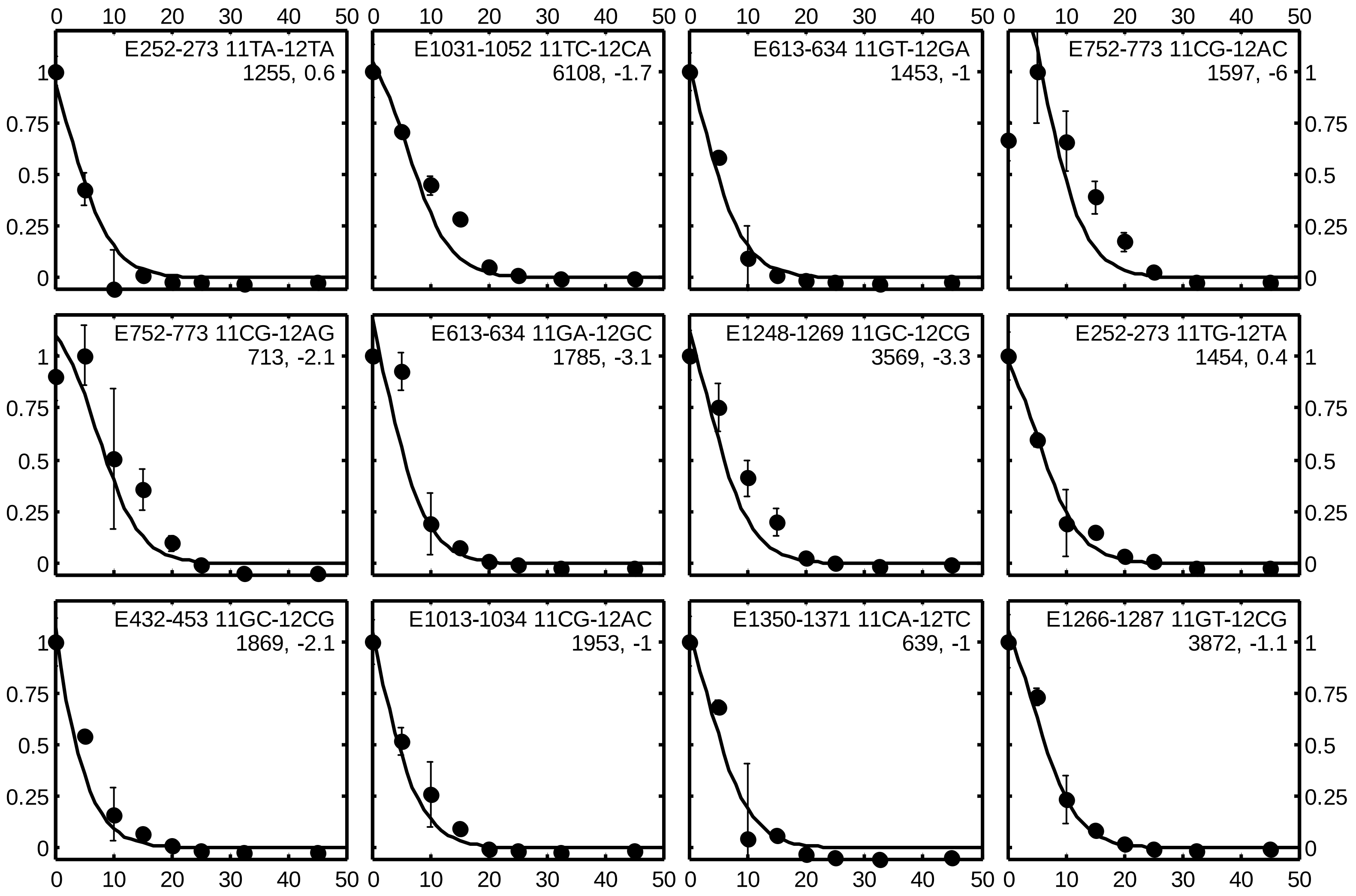
E252-273 11TA-12TA
1255, 0.6
E1031-1052 11TC-12CA
6108, -1.7
E613-634 11GT-12GA
1453, -1
E752-773 11CG-12AC
1597, -6
E752-773 11CG-12AG
713, -2.1
E613-634 11GA-12GC
1785, -3.1
E1248-1269 11GC-12CG
3569, -3.3
E252-273 11TG-12TA
1454, 0.4
E432-453 11GC-12CG
1869, -2.1
E1013-1034 11CG-12AC
1953, -1
E1350-1371 11CA-12TC
639, -1
E1266-1287 11GT-12CG
3872, -1.1
0 10 20 30 40 50
0 0.25 0.5 0.75 1

Figure S1F

Probes 33-44:

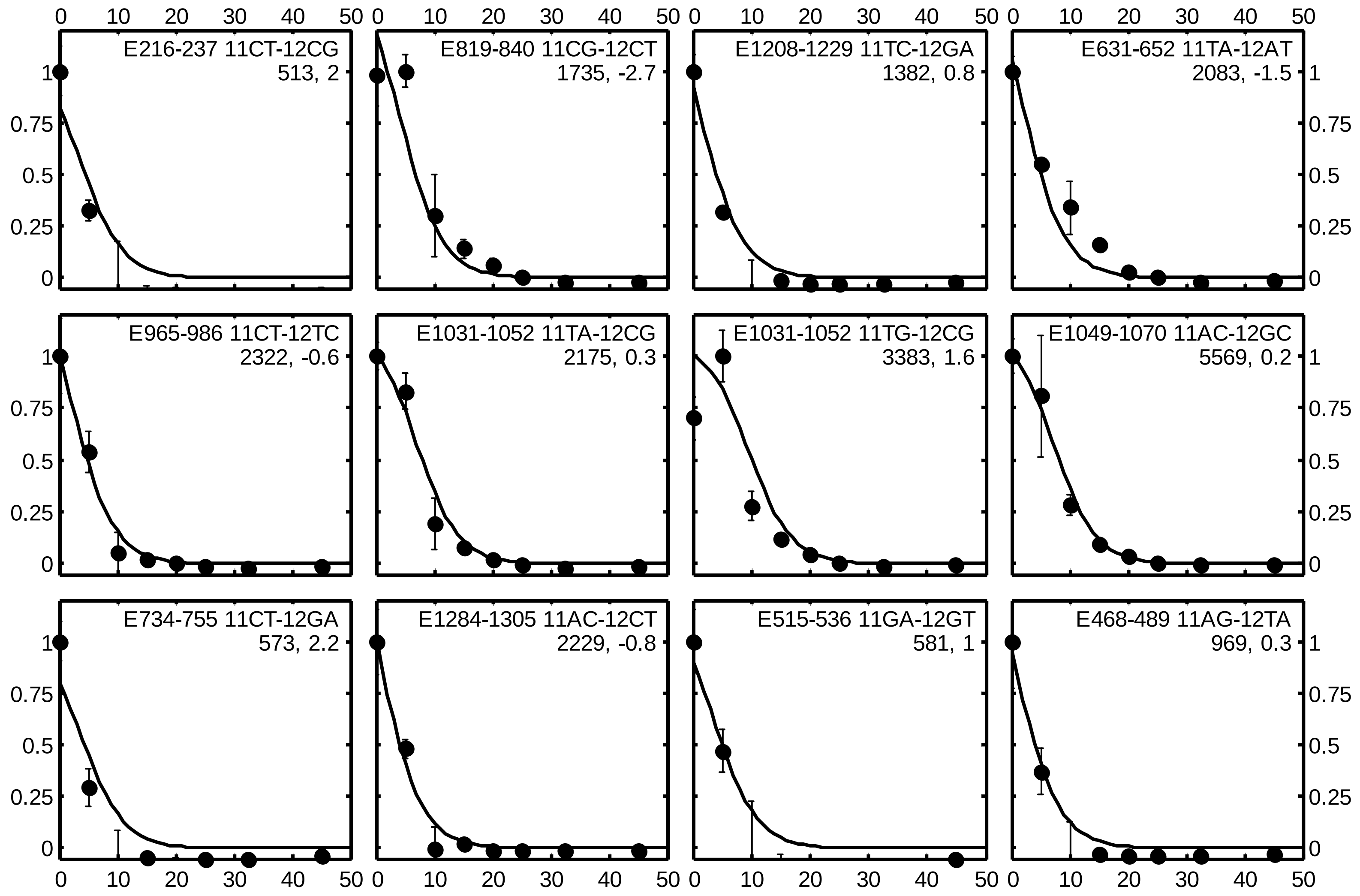

Probes 45-56:

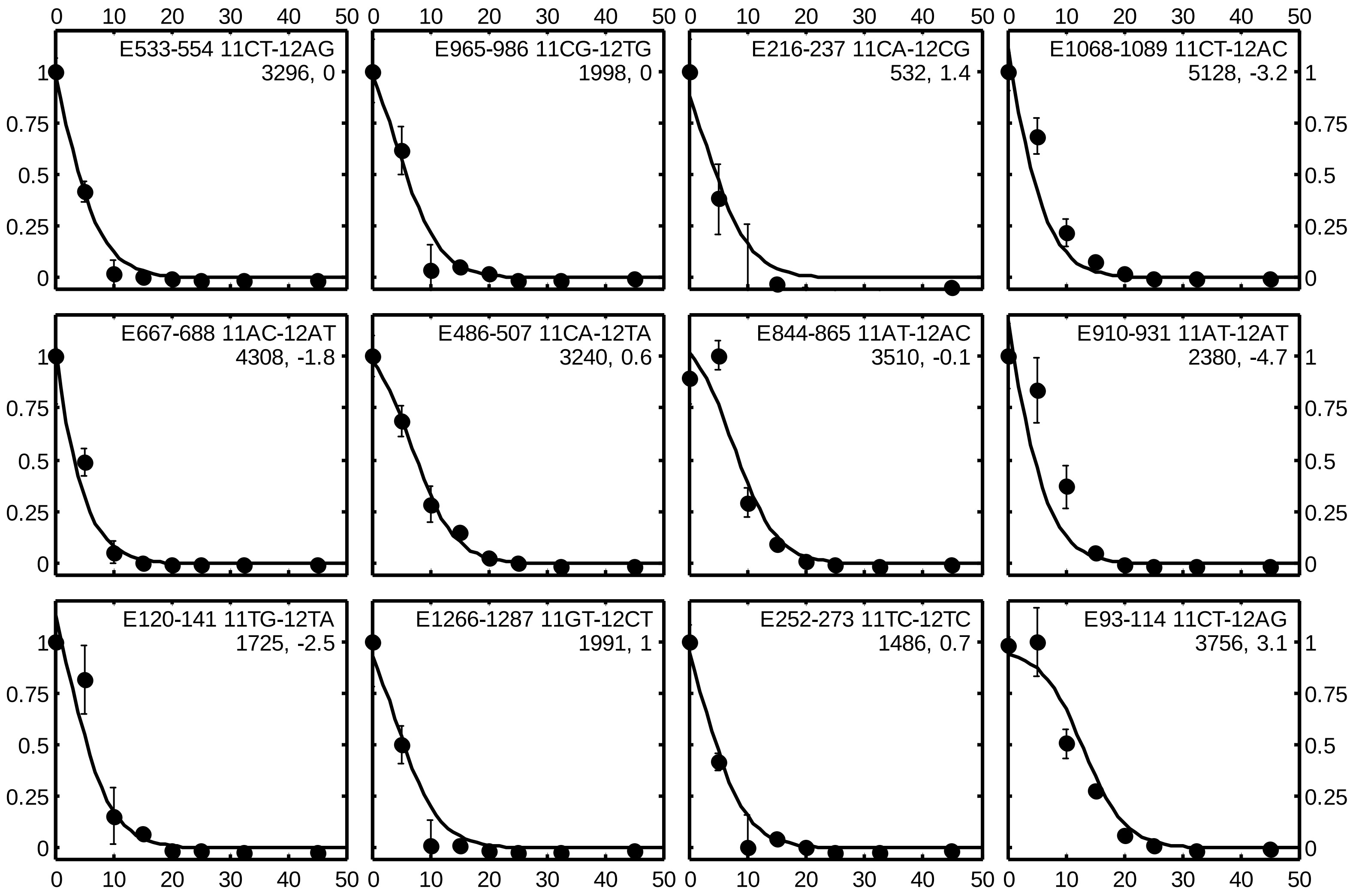
E533-554 11CT-12AG
3296, 0
E965-986 11CG-12TG
1998, 0
E216-237 11CA-12CG
532, 1.4
E1068-1089 11CT-12AC
5128, -3.2
E667-688 11AC-12AT
4308, -1.8
E486-507 11CA-12TA
3240, 0.6
E844-865 11AT-12AC
3510, -0.1
E910-931 11AT-12AT
2380, -4.7
E120-141 11TG-12TA
1725, -2.5
E1266-1287 11GT-12CT
1991, 1
E252-273 11TC-12TC
1486, 0.7
E93-114 11CT-12AG
3756, 3.1
0
10
20
30
40
50
0
0.25
0.5
0.75
1

Probes 57-68:

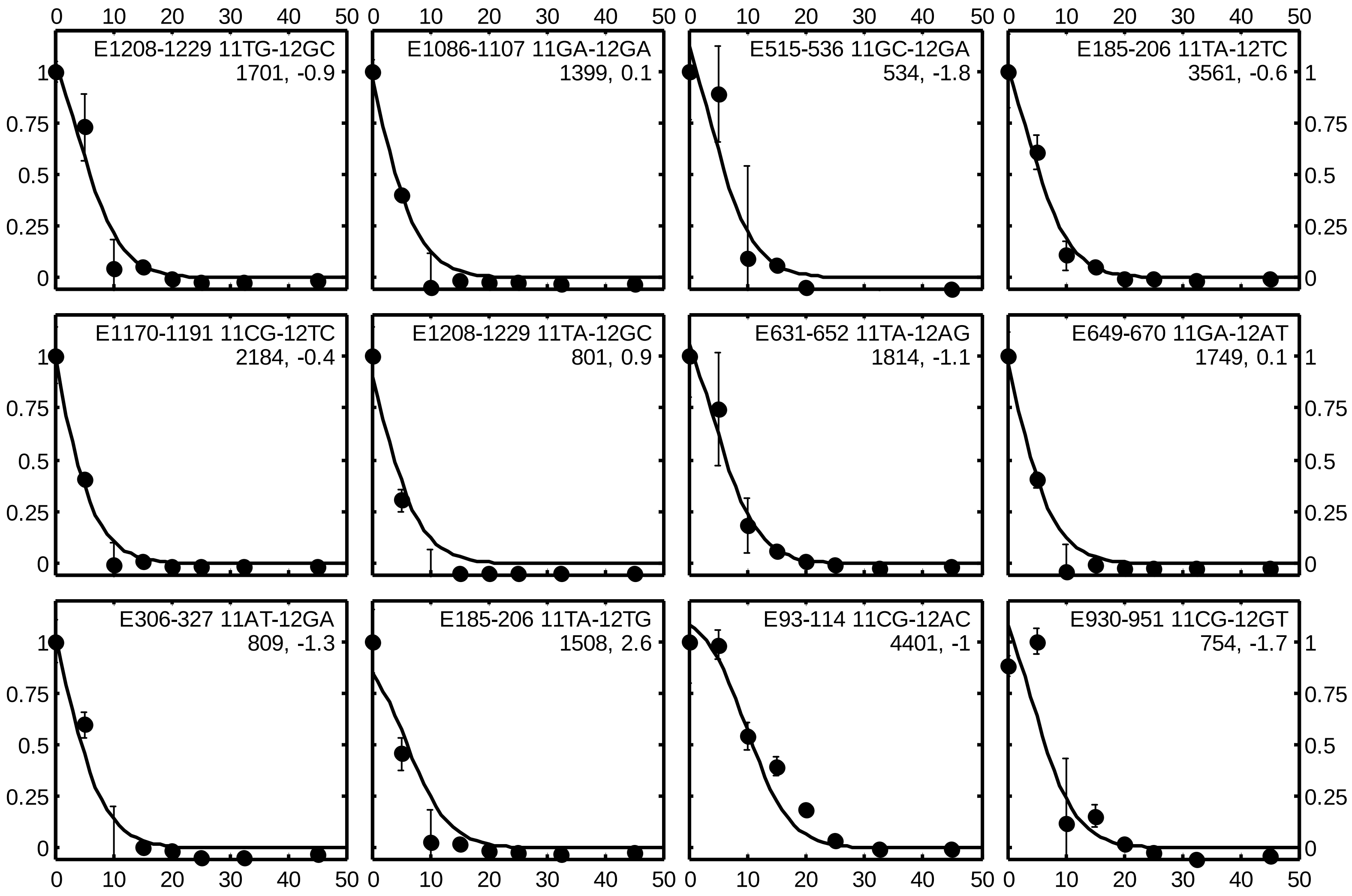
E1208-1229 11TG-12GC
1701, -0.9
E1086-1107 11GA-12GA
1399, 0.1
E515-536 11GC-12GA
534, -1.8
E185-206 11TA-12TC
3561, -0.6
E1170-1191 11CG-12TC
2184, -0.4
E1208-1229 11TA-12GC
801, 0.9
E631-652 11TA-12AG
1814, -1.1
E649-670 11GA-12AT
1749, 0.1
E306-327 11AT-12GA
809, -1.3
E185-206 11TA-12TG
1508, 2.6
E93-114 11CG-12AC
4401, -1
E930-951 11CG-12GT
754, -1.7

Probes 69-80:

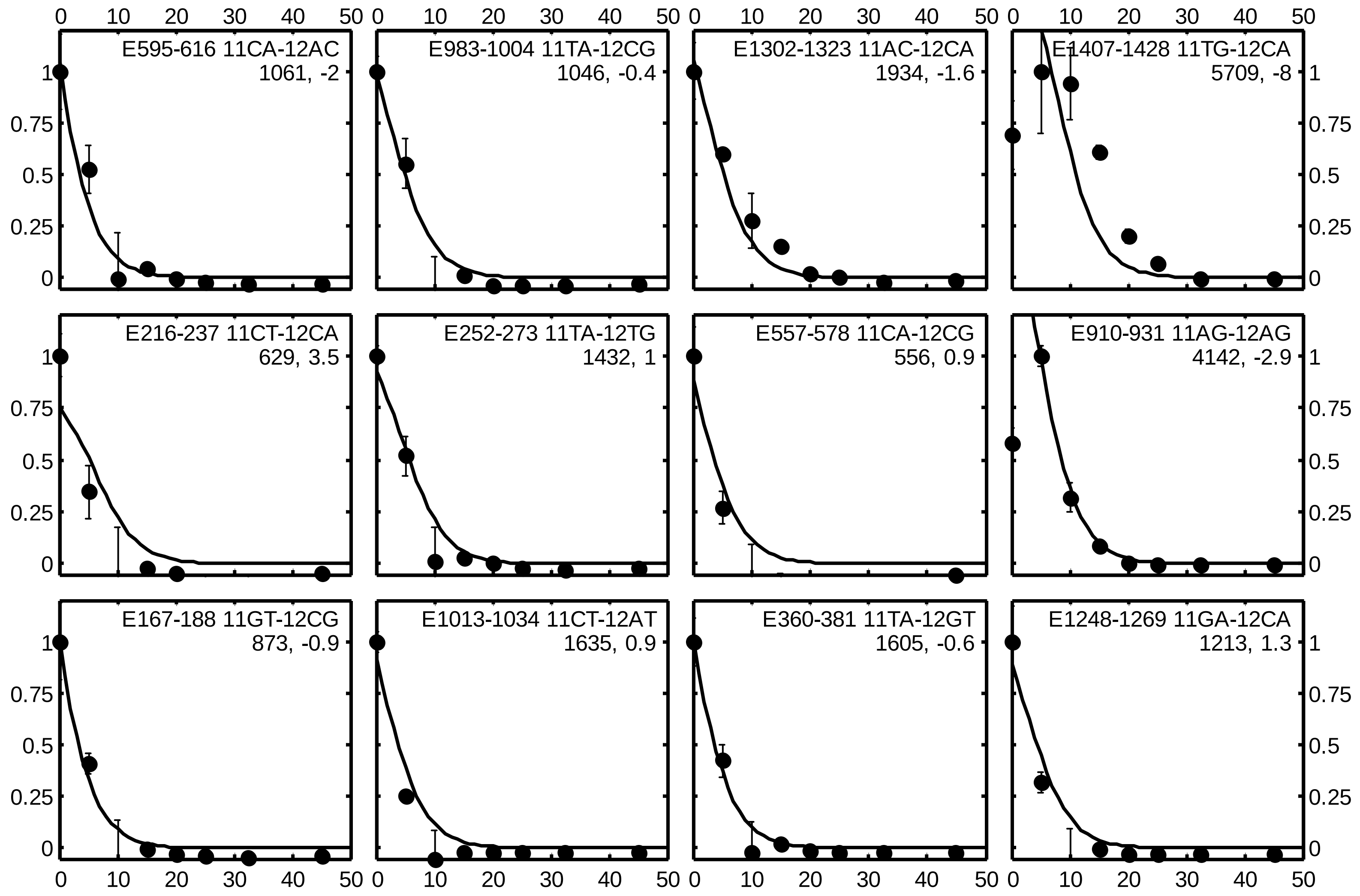
E595-616 11CA-12AC
1061, -2
E983-1004 11TA-12CG
1046, -0.4
E1302-1323 11AC-12CA
1934, -1.6
E1407-1428 11TG-12CA
5709, -8
E216-237 11CT-12CA
629, 3.5
E252-273 11TA-12TG
1432, 1
E557-578 11CA-12CG
556, 0.9
E910-931 11AG-12AG
4142, -2.9
E167-188 11GT-12CG
873, -0.9
E1013-1034 11CT-12AT
1635, 0.9
E360-381 11TA-12GT
1605, -0.6
E1248-1269 11GA-12CA
1213, 1.3
0 10 20 30 40 50
0 0.25 0.5 0.75 1

Probes 81-92:

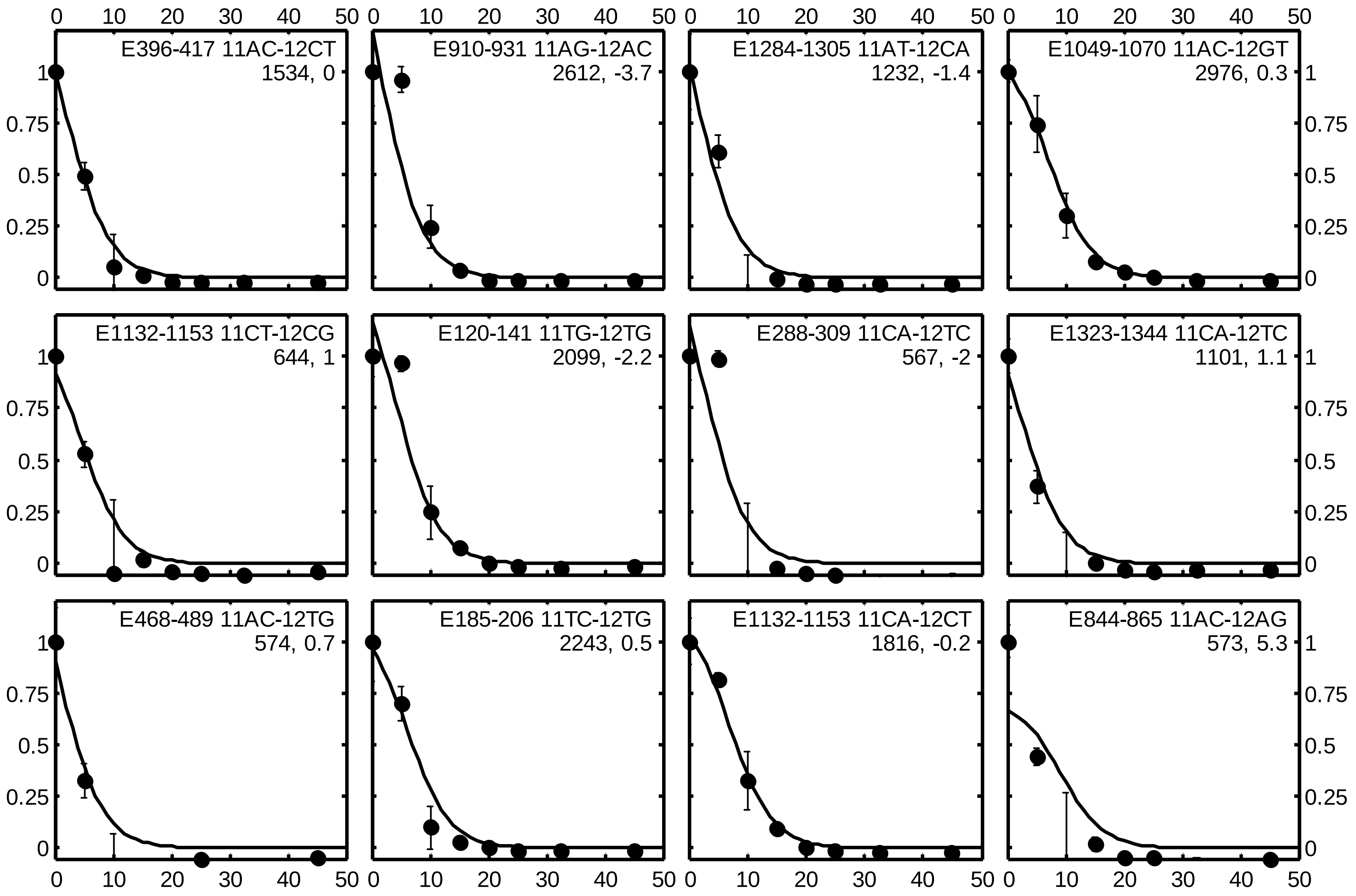
E396-417 11AC-12CT
1534, 0
E910-931 11AG-12AC
2612, -3.7
E1284-1305 11AT-12CA
1232, -1.4
E1049-1070 11AC-12GT
2976, 0.3
E1132-1153 11CT-12CG
644, 1
E120-141 11TG-12TG
2099, -2.2
E288-309 11CA-12TC
567, -2
E1323-1344 11CA-12TC
1101, 1.1
E468-489 11AC-12TG
574, 0.7
E185-206 11TC-12TG
2243, 0.5
E1132-1153 11CA-12CT
1816, -0.2
E844-865 11AC-12AG
573, 5.3

Probes 93-100:

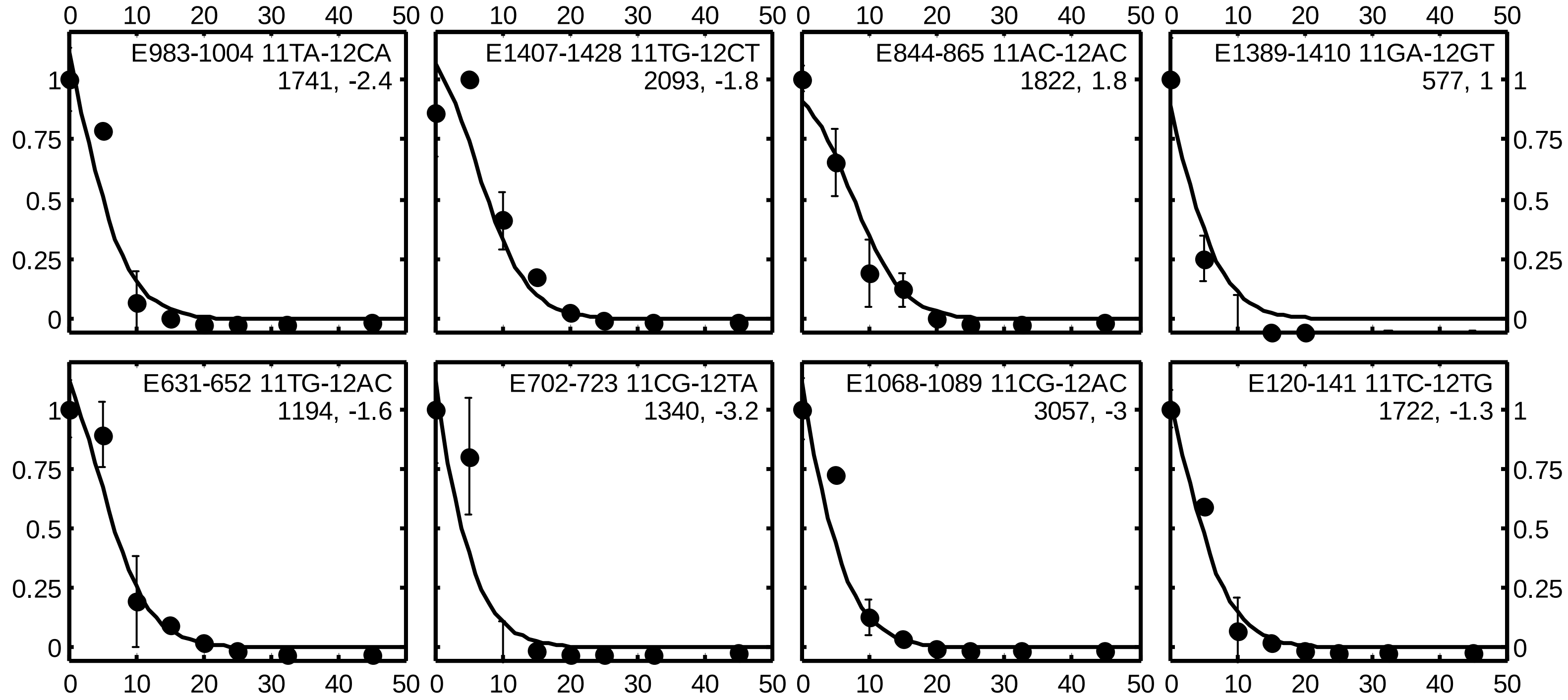

Supplement: Figure S1 — Figures of additional experimental and theoretical formamide melting profiles. (PDF) [file pone.0043862.s001.pdf]
