# Supplementary material for: Modeling Formamide Denaturation of Probe-Target Hybrids for Improved Microarray Probe Design in Microbial Diagnostics
Source: PLoS One. 2012 Aug 27;7(8):e43862. doi: 10.1371/journal.pone.0043862 (PMC3428302; doi:10.1371/journal.pone.0043862)
Supplement: Figure S2 — Signal saturation with highly concentrated target. (PDF) [file pone.0043862.s002.pdf]

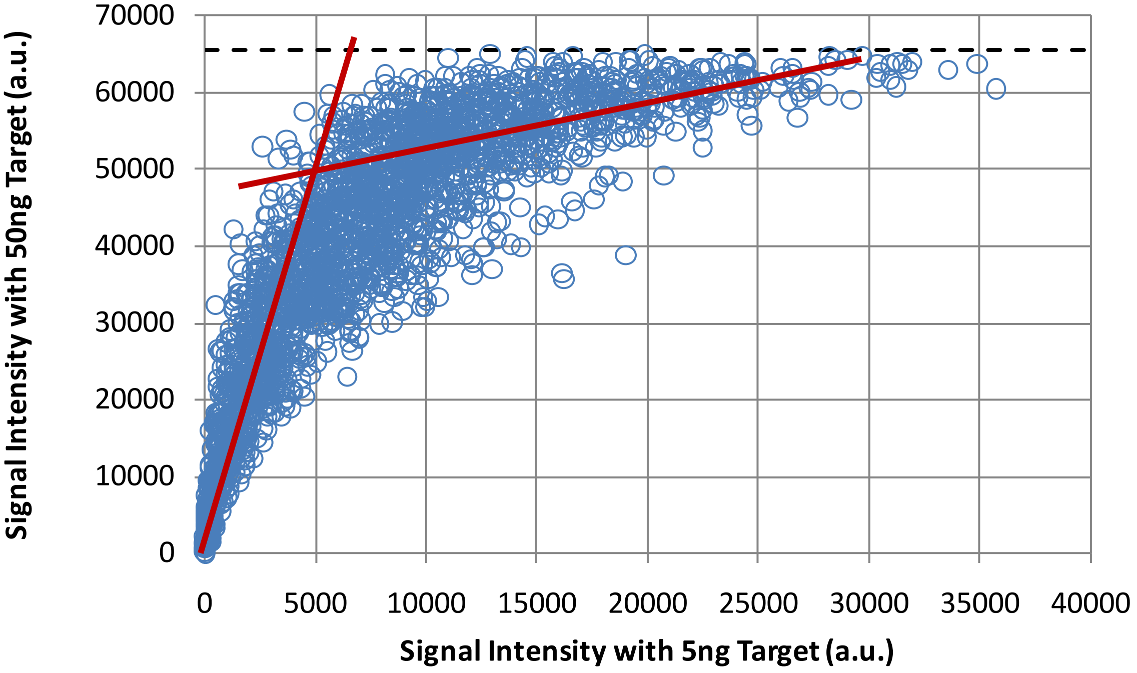

Figure S2. Signal saturation with highly concentrated target. Plot shows fluorescence intensity of probes in TileE and Length sets (Table 1) obtained with 15% formamide in the hybridization buffer. The steeper red line, with a slope of 10, indicates the lower fluorescence intensity values (<40,000 florescence units) on the y-axis are consistent with a 10-fold increase in target concentration. The other red line is drawn with an arbitrary slope to show the signal saturation at higher signal levels. The dashed line indicates the maximum signal intensity that can be recorded. Bright probes converge to this value for the concentrated target.
