# Supplementary material for: Modeling Formamide Denaturation of Probe-Target Hybrids for Improved Microarray Probe Design in Microbial Diagnostics
Source: PLoS One. 2012 Aug 27;7(8):e43862. doi: 10.1371/journal.pone.0043862 (PMC3428302; doi:10.1371/journal.pone.0043862)
Supplement: Figure S3 — Curve fitting with different methods to align theoretical curves with normalized experimental profiles. (PDF) [file pone.0043862.s003.pdf]

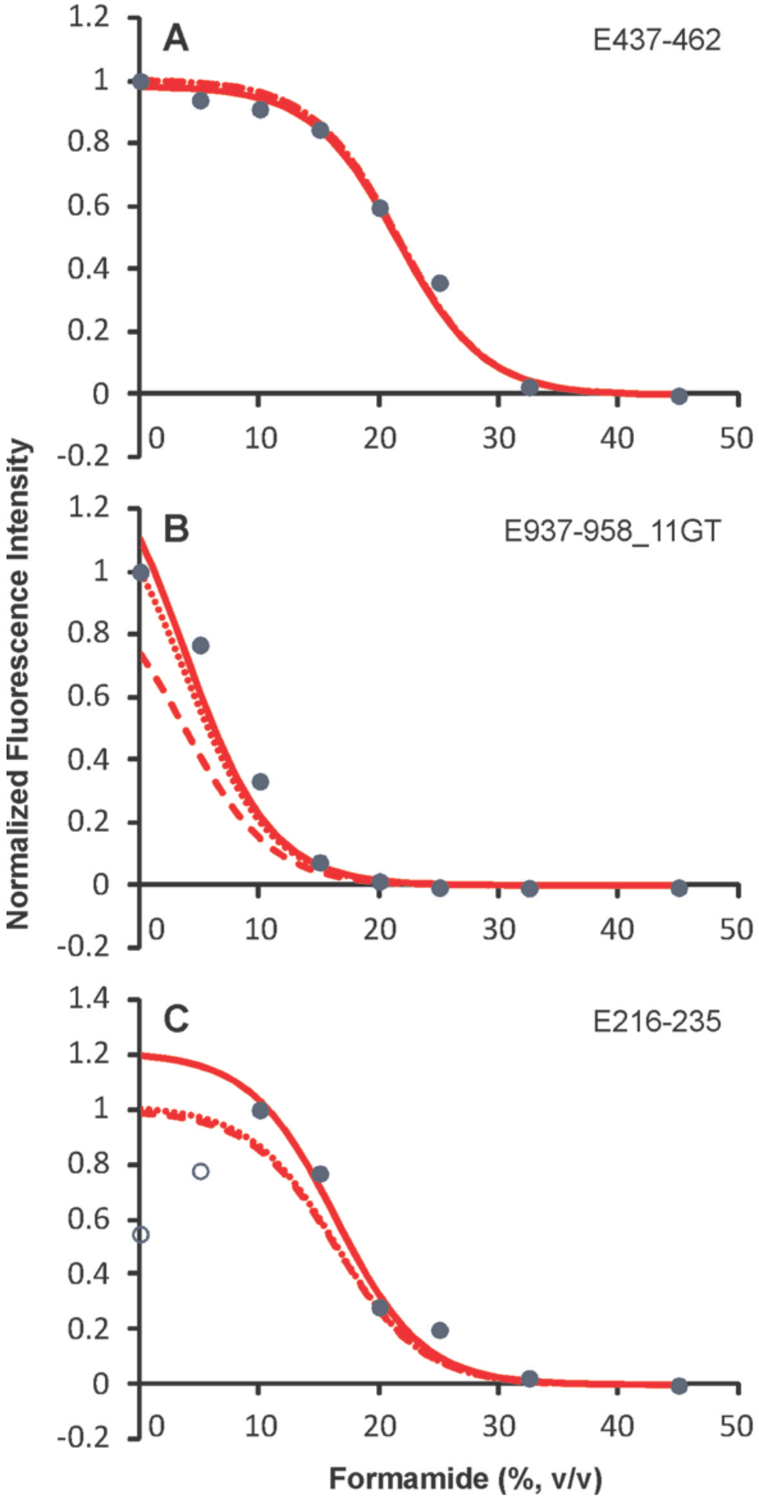

Figure S3. Curve fitting with different methods to align theoretical curves with normalized experimental profiles. The γ-factor approach preferred in modeling (best-fitted γ values, Equation 3) (solid curves) is

compared to the case without any adjustment ($\gamma = 1.0$) (dashed curves) and to an alternative technique wherein the theoretical curve is normalized to its maximum (γ = 1/[hybridization efficiency at 0% formamide]) (dotted curves; see also TextS1). **(A)** Example probe showing no significant difference between different methods due to the presence of a nearly perfect sigmoidal pattern (best fitted $\gamma = 1.0$). **(B)** Example probe with a truncated sigmoidal profile because of early melting. With $\gamma = 1.0$, the calculated hybridization efficiency value (e.g., 0.74 at 0% formamide) does not match the normalized experimental data, which is always 1 at maximum signal response. The original best-fitting γ approach and the alternative fixed γ method can align the theoretical profile with the experimental data (best-fitted $\gamma = 1.5$). **(C)** Example probe without a full sigmoidal profile due to signal increase at lower formamide concentrations, a frequent case which has been observed in other hybridization systems before and attributed to possible kinetic limitations posed by secondary structures of probe and target (see main text). Only the best-fitted γ can buffer this effect as it is not limited by the maximum of the experimental or theoretical profiles. Open circles indicate data points ignored during curve-fitting (see Results for the pertaining rule) because the model used (Equation 1 in main text) is not meant to capture the kinetic limitations observed at low formamide concentrations.
