# Supplementary material for: Modeling Formamide Denaturation of Probe-Target Hybrids for Improved Microarray Probe Design in Microbial Diagnostics
Source: PLoS One. 2012 Aug 27;7(8):e43862. doi: 10.1371/journal.pone.0043862 (PMC3428302; doi:10.1371/journal.pone.0043862)
Supplement: Figure S4 — Figures for Text S2. (PDF) [file pone.0043862.s004.pdf]

Figures for Text S2

A

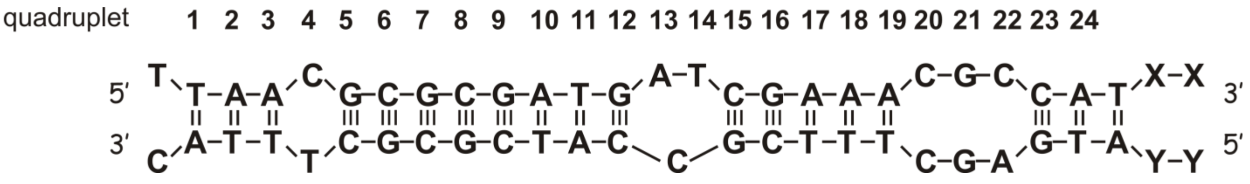

B

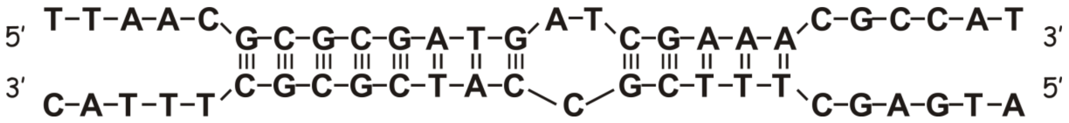

C

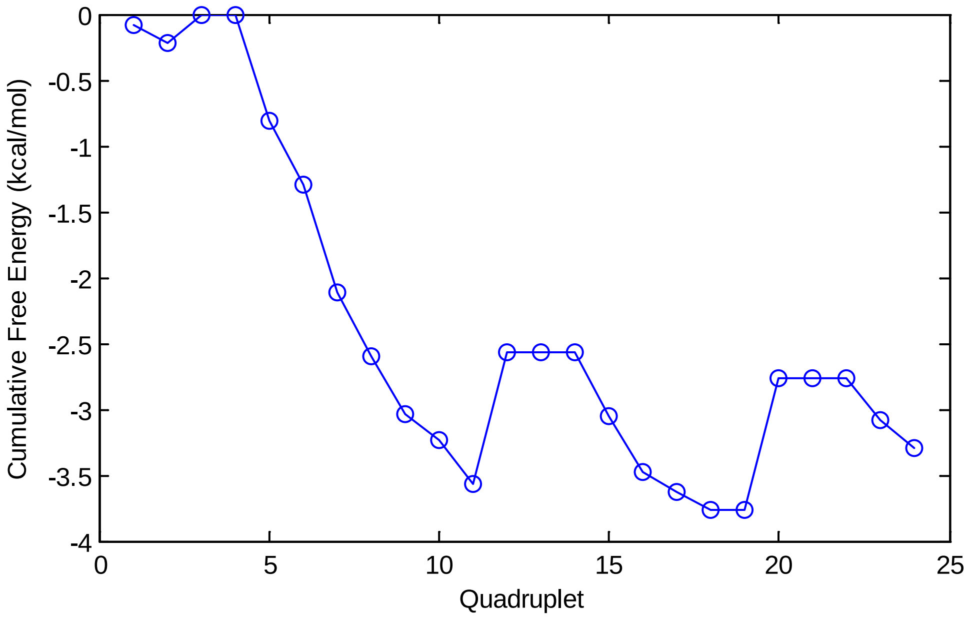

D

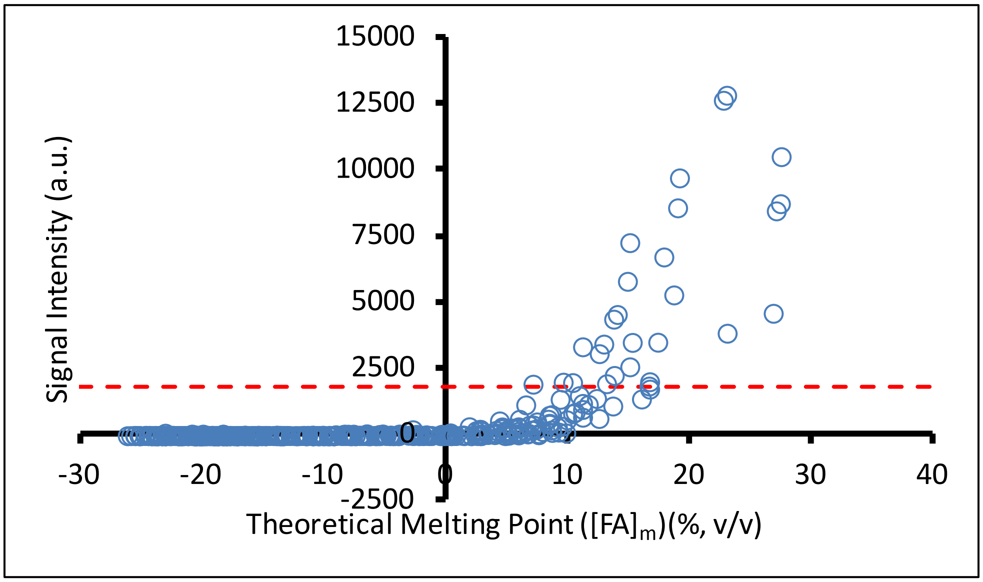

Figure S4. (A) Example duplex for the demonstration of free energy calculations. The last two pairs of mismatching nucleotides are added for algorithmic reasons to form terminal quadruplets. **(B)** Relaxed conformation of the example duplex with minimum predicted free energy. **(C)** Graphical representation of free energy calculation for the example duplex (see Equations S2, S3). Positive cumulative values are zeroed at the 3rd and 4th quadruplets. Minimum free energy is attained at the 18th quadruplet. Total free energy is obtained by adding the initiation free energy to the minimum value in the graph (see also Equation S3). **(D)** Experimental verification of the algorithm developed for the calculation of free energy changes of duplexes involving complex conformations. Signal intensities of *R. sphaeroides* probes obtained with 5 ng *E. coli* target and 15% formamide in the hybridization buffer are plotted against theoretical melting points ($[FA]_m$). For melting point calculation, the full sigmoidal profile was generated with the LFEM even if the upper plateau was in negative formamide values, and the mid-point of this curve was taken (and hence the values are different from $[FA]_{1/2,pred}$ used in the manuscript, which is calculated from curves always starting at 0% formamide). Only probes with predicted melting points around or greater than 15% show strong fluorescence intensities above 1750 units, a threshold defined in the Discussion section (dashed line). Most duplexes have hypothetical melting points less than 0% formamide, consistent with their signal intensities close to the background.
