# Supplementary material for: Modeling Formamide Denaturation of Probe-Target Hybrids for Improved Microarray Probe Design in Microbial Diagnostics
Source: PLoS One. 2012 Aug 27;7(8):e43862. doi: 10.1371/journal.pone.0043862 (PMC3428302; doi:10.1371/journal.pone.0043862)
Supplement: Table S2 — Extended free energy rules for quadruplets of nucleotide pairs in a DNA duplex. (PDF) [file pone.0043862.s006.pdf]

Table S2. Extended free energy rules for quadruplets of nucleotide pairs in a DNA duplex.

**Class**	**Quadruplet** [a]	**ΔG°** [b]	**Description**
**1**	●●**	Table S1A	Nearest neighbor in the first two positions.
**2**	●^●●	Table S1B	Mismatch loop based on the triplet in the first three positions.
**3**	●-●●	Table S1C	Bulged mismatch loop based on the triplet in the first three positions.
**4**	●^^●	Table S1D	Tandem mismatch.
**5**	●^-●<br>●-^●<br>●--●	1.0	Bulged tandem mismatch loop.
**6**	###●	1.0	End of a mismatch series with at least three mismatches.
**7**	●#●#	1.0	Adjacent mismatch loops interfering with at least four nearest neighbors.
**8**	#**#	0.0	No additional penalty since used mismatch loop information is contained in neighboring quadruplets.
**9**	#●●●	0.0	No additional penalty since used mismatch loop information is contained in neighboring quadruplets.
**10**	##●●	0.0	No additional penalty since used mismatch loop information is contained in neighboring quadruplets.
**11**	#●#●	0.0	No additional penalty since used mismatch loop information is contained in neighboring quadruplets.
**12**	●###	0.0	Beginning of a mismatch series with at least three mismatches. Penalty is placed at the end of the mismatch series (see Type 6).

[a] ●, matching pair; **^**, base to base mismatched pair; **-**, gap to base mismatch (bulged mismatch); **#**, any mismatch; *****, any pair.

[b] For studied conformations, corresponding table of parameters is indicated. For others, approximations are provided in kcal/mol.
