# Supplementary material for: Modeling Formamide Denaturation of Probe-Target Hybrids for Improved Microarray Probe Design in Microbial Diagnostics
Source: PLoS One. 2012 Aug 27;7(8):e43862. doi: 10.1371/journal.pone.0043862 (PMC3428302; doi:10.1371/journal.pone.0043862)
Supplement: Text S1 — Best-fitted γ Factors and Additional Statistical Tests. (PDF) [file pone.0043862.s007.pdf]

Best-fitted γ Factors and Additional Statistical Tests

The mathematical framework of this study was devised to fit theoretical denaturation curves to normalized experimental formamide series. When the experimental data show a nearly perfect sigmoidal profile as depicted in Figure S3A, the underlying assumption is that the upper plateau of the normalized profile represents a hybridization efficiency of 1, and similarly, the lower plateau a hybridization efficiency of 0. Thus the experimental data can be directly matched with a theoretical curve obtained from calculated hybridization efficiencies. However, in the absence of a full sigmoidal profile (e.g., Figures S3B and S3C), there is no firm basis for the actual experimental hybridization efficiency and the value of 1 in the normalized experimental series only represents the maximum observed signal. Therefore, the theoretical curve also needs to be modified to be matched with the experimental data. This was done by including a γ factor in the estimation of theoretical denaturation profiles, as described by Equation 3 (see Methods). There are three mathematical options for the determination of γ factors in Equation 3. First, it is possible to simplify the mathematical model by setting $\gamma = 1$ for all probes. This corresponds to the abovementioned direct matching of theoretical hybridization efficiency values with normalized experimental data and is not effective when the experimental profile does not have a complete sigmoidal shape. Second, γ can be calculated directly from the theoretical hybridization efficiency calculations so that the maximum predicted efficiency takes a value of 1, consistent with the normalization of the experimental data. Third, it is possible to allow the calculation of γ factors by a secondary fitting of the experimental data to the modeling output. The latter approach was preferred in this study because it allows for a better representation of the experimental data in incomplete sigmoidal profiles (Figure S3B) and in profiles in which kinetic limitations may affect the hybridizations at low formamide concentrations (Figure S3C). However, allowing for best-fitting of γ factors may seem to overparameterize the models developed. Overparameterization is not a rare problem in microarray data modeling as was mentioned previously [1]. In this study, the degree of freedom lost due to γ factors was taken into account by the key statistic used in model comparison (s^2, Equation 5) and the cross validation tests carried out during modeling proved that the derived thermodynamic parameters were physically meaningful (Table 2). Furthermore, we performed additional statistical tests (presented in this section) to clearly and directly demonstrate that the predictive power of our models is driven by thermodynamic parameters and not the γ factors, and therefore, that it is statistically legitimate to estimate these factors during the curve-fitting procedure.

The additional analyses involved twelve statistical tests summarized in Table 3 (designated T1-T12). First (T1, Table 3), we show the effect of setting $\gamma = 1$ in Equation 3 for all probes in model M3. Compared with the original results in Table 2 for M3, this approach increased the overall error squares by about 50% (from 0.0081 to 0.0118) with a modest decrease in R^2 (from 0.95 to 0.93). To further evaluate the influence of γ factors in simulations, we performed T2 (Table 3). In this case, original best-fitted γ factors from M3 were permuted between probes and the results for the key statistics were repeatedly obtained for different randomized permutations. The average ε_{ov}^2 and R^2 converged to the respective values of 0.0157 and 0.90 after about 150 permutations, showing further decrease in the agreement between theoretical curves and experimental data, although the model sensitivity to γ factors was still

modest. It should be noted that these tests did not affect the predictive error for half-denaturation points ($err[FA]_{1/2}$; Table 2 and Table 3) as the melting point of a theoretical curve is mathematically independent of γ factors (see main text). In comparison, the permutation of nearest neighbor free energy parameters largely degrades the predictive power of model M3, as shown by test T3 (γ factor best-fitting is in effect in this test). Converging values after about 400 permutations showed more than quadrupled error squares and a relatively large decrease in R^2 to 0.78 (Table 3), as compared to the results in Table 2. More importantly, the average mid-point distance between theoretical and experimental curves increased from 2.1% to 5.7% formamide and nearly half of the probes had a distance larger than 5% formamide, which we considere as the effective threshold for useful predictions. Given that only 6.6% of the probes were above this threshold in the original calibrated model (Table 2), the role of the free energy calculations in predictive power is clear. However, the total effect of free energy predictions is underestimated by this analysis as the model is still able derive useful information from probe sequence in the form of probe length. Since there is a general correlation between probe length and melting point, and since longer probes always have a larger sum of nearest neighbor free energies, some of the variation can still be captured by the probe free energy calculations. Thus, we measured the overall sensitivity of M3 to ΔG^o values by permuting probe sequences during the analysis (T4, Table 3). This corresponds to permuting the calculated ΔG^o values between the probes in addition to permuting the nearest neighbors. The result is poorer fitting quality with an R^2 of 0.68 and with the majority of probes having a >5% error at the melting point. Clearly, the model has no useful predictive power without using the thermodynamic information derived from probe sequences.

Despite the loss of predictive ability after free energy permutations, the relatively large R^2 value of 0.68 suggests that the model can still capture the majority of experimental variation. How is this possible if γ factors also cannot explain the variation? The answer must be in the two remaining modeling parameters for M3, namely, the effective probe concentration ($\{P\}_o$) and the denaturant *m*-value. Indeed, when these two parameters are randomized in their physically meaningful ranges (assumed as 10^{-12} to 1M for $\{P\}_o$ and 0-1 kcal/mol/% for *m*), R^2 converges to a negative value (T5, Table 3) indicating that the model cannot follow the experimental trends any more. Thus, the calibrated $\{P\}_o$ and *m* values (together with free energy values in the right scale) capture most of the experimental variation by defining the general sigmoidal shape of the denaturation profiles, while the exact values of nearest neighbor free energies adjust the positioning of the curves along the x-axis (formamide) to best fit the experimental data. The γ factors account for a smaller portion of the variation by adjusting the curves along the y-axis (normalized signal intensity). Combined with the fact that the best-fitted and cross-validated nearest neighbor rules of M3 correlate strongly with the original in-solution values (Figure 4), these analyses demonstrate the unequivocal physical meaning of the thermodynamic parameters within our modeling framework.

Since T1 showed that γ factors had a minor contribution to the goodness of fitting, an inevitable question is what would be the effect of removing γ factors during model development. To answer this question for M1 and M3, we performed T6 and T7, which successfully re-calibrated and validated these models using $\gamma = 1.0$ in Equation 3 for all probes (Table 3). Although the decrease in the goodness of fitting was slightly larger for M1 (based on R^2 values in Table 3 compared to those in Table 2), the relative strengths of the two models did not change appreciably with respect to the original modeling practice shown in Table 2. Moreover, the new best-fitting values for the nearest neighbor free energies of

M3 were nearly identical to the original ones (linear relationship: y = 1.0702x - 0.0002, R^2 = 0.999; data not shown). Thus, omitting γ factors would not have caused significant changes in the results and conclusions from M1 and M3 models.

The obvious next question is why γ factors are preferred. Part of this answer is in Figure S3B, where the best-fitted γ factor is significantly different from 1.0 (γ = 1.5 in this case). In this case, the theoretical hybridization efficiency values for the range of formamide are always lower than 1 and a full sigmoidal curve is not obtained. Therefore, a multiplier is needed to align the maximum point of the theoretical curve with the normalized experimental profiles. The probe set used in M1 and M3 (perfect matches) has only a few of these cases (for 93% of probes: 0.8< γ <1.2), and thus, the overall curve-fitting is less sensitive to the γ factor adjustment (see Figure S1A for a subpopulation of probes in this set). To realize the potential of γ factors, we need to use a set that has a significant number of probes as in Figure S3B. Since mismatched probes are more likely to behave this way, we tested the effect of removing γ factors on M5, our model that addresses central mismatches (T8, Table 3) (for 28% of probes = γ >1.2; see Figure S1B for a subpopulation of probes in this set). In this case, the average error squares (0.0147) was nearly doubled compared to results in Table 2 (M5, central single mismatches), with a decrease in R^2 from 0.94 to 0.90. Furthermore, the permutation of γ factors (test T9) effectively reduced fitting quality as expected (ε_{ov}^2 = 0.028, R^2 = 0.82). Thus, the goodness of fits in the mismatched probe set of M5 were more sensitive to γ factors than the perfect match set of M3.

We also explored the alternative estimation of γ factors that does not need best-fitting. This involves the simple calculation of the factor as the inverse of the maximum predicted hybridization efficiency (always attained at 0% formamide) which effectively means that the theoretical profiles are adjusted to have a maximum normalized value of 1. The 10th test (T10, Table 3) shows that calculating γ this way for M5 would give lower residual squares and higher R^2 than what is achievable without any adjustment (i.e., in comparison to T8, where γ = 1) and the goodness of fitting is marginally different from the original M5 results in Table 2 (central single mismatches). In comparison, the permutation of the 104 loop free energy parameters of M5 results in a greater loss of goodness of fitting (T11, Table 3), with the additional decrease in the percentage of probes showing good half-denaturation point predictions from 94.5 to 82%. Note that this is still a modest degradation of fitting quality compared to the effect of permuting nearest neighbor free energies in M3 (see T3, Table 3), because the true nearest neighbors that M5 inherits from the parent model M3 dominate the overall probe free energy calculations (see Methods). In other words, M5 is able to capture most of the experimental variation by calibrated thermodynamic parameters inherited from M3, while the additional loop free energy parameters of M5, by their definition, fine tune the position of the theoretical profiles along the x-axis (formamide) to best fit the experimental data. Therefore, the model simulations are primarily driven by thermodynamic parameters while γ factors are secondary parameters that can either be calculated from the predictions at 0% formamide or best-fitted for each probe.

As a final test, we performed T12 (Table 3) to show that the fixed γ factor approach could indeed be used in model M5 to replace best-fitted γ factors without significantly affecting the original fitting performance. This test was clearly positive, with the average predictive error in half-denaturation points not different than the original model (see Table 3 and Table 2). In addition, the loop free energy values obtained this way showed only small differences from the original ones (linear relationship: y = 1.05x -

0.13, R^2 = 0.996; data not shown). Then why do we favor best-fitted γ factors against fixed values to avoid the addition of new parameters to modeling? This is partially answered by Figure S3C showing a frequent case where an increase in signal intensity is observed at lower formamide points (see Figure S1A for many examples), possibly due to kinetic limitations posed by secondary structures (see main text). This artifact obscures the melting behavior definable by our two-state model, as the maximum observed signal intensity probably does not match 100% hybridization efficiency, yet it has to be assigned the value of 1 in the normalized profile (Figure S3C). The situation can also be described as a missing upper plateau of the sigmoidal profile, which is better handled by the best-fitted γ factor as clearly seen in Figure S3C. Furthermore, the alternative calculation of γ as the inverse of the hybridization efficiency at 0% formamide always fixes both theoretical and experimental maxima to a value of 1, giving a single experimental value in a formamide series (that with maximum signal intensity) the control over the fitting quality for the probe. This can amplify the effect of experimental noise on parameter estimations. Instead, best-fitted γ factors align the theoretical curve with the experimental profile using the information in the entire series, thereby minimizing the effect of experimental noise. In retrospect, the small differences in thermodynamic parameters derived with and without γ factors reflect the effect of these experimental artifacts on the latter. Therefore, the parameters obtained with best-fitted γ factors in this study are our best estimates given the microarray data.

In conclusion, the best-fitting approach to calculate γ factors was an effective way of buffering experimental artifacts of formamide denaturation without overparameterizing the models developed. Modeling predictions were almost fully driven by thermodynamic parameters that reflect our best estimates from data.

1. Pozhitkov AE, Tautz D, Noble PA (2007) Oligonucleotide microarrays: widely applied--poorly understood. Briefings in functional genomics & proteomics 6: 141-148.
