# Supplementary material for: Modeling Formamide Denaturation of Probe-Target Hybrids for Improved Microarray Probe Design in Microbial Diagnostics
Source: PLoS One. 2012 Aug 27;7(8):e43862. doi: 10.1371/journal.pone.0043862 (PMC3428302; doi:10.1371/journal.pone.0043862)
Supplement: Text S2 — Free energy calculations with nucleotide quadruplets. (PDF) [file pone.0043862.s008.pdf]

Free Energy Calculations with Nucleotide Quadruplets

To calculate the free energy change for any given probe/target site duplex, we established a simple algorithm that uses the free energy rules of nearest neighbors and mismatch loops derived during modeling (see Results) and approximations for other (more complex, less stable) conformations. The algorithm divides the DNA duplex into quadruplets of neighboring base pairs (matches and mismatches) and sums the free energy contributions of each quadruplet together with the initiation free energy, as shown in Equation S1. Here, *q* represents the number of quadruplets that form the duplex such that the dangling terminal mismatches are removed from the duplex in line with the thermodynamic model developed. There are 81 possible quadruplet types in terms of base-matching properties (3X3X3X3, for the four positions each with three possibilities including a matching pair, base to base mismatch pair, and base to gap mismatch [insertion or deletion]). These are consolidated into 12 major classes as shown in Table S2. Relaxation of terminal regions due to positional mismatch stability (see Results) is also included in the algorithm. See below for an example calculation.

$$\Delta G^{o}_{duplex} = \Delta G^{o}_{quadruplets} + \Delta G^{o}_{ini} = \sum_{i=1}^{q} \Delta G^{o}_{i} + \Delta G^{o}_{ini} \qquad \text{(S1)}$$

The advantage of using a quadruplet as the smallest unit is that, the most complex conformation studied (i.e., tandem mismatches; see Results) is covered, while the predictive approach is extended to other forms without a long and complex list of all possible nucleotide combinations. Thus, our method allows the fast computation of the hybridization efficiency of a probe against thousands of target and non-target sequences that may be encountered during microarray design for microbial ecology applications. The free energy rules in Table S2 provide conservative approximations for conformational stability, i.e., the free energy penalties applied to mismatch loops that were not systematically studied probably underestimate the actual destabilizing effect. In >95% of duplexes with complex conformations, all irregular mismatch types end up being lost in relaxed terminals, which implicates a high confidence in free energy calculations since the penalties that cause relaxation are probably underestimated. In the remaining cases, the complex conformations are generally centrally positioned and associated with a very low melting point (<5% formamide in >85% of the cases), making the predictive accuracy less important. Experimental verification of the free energy algorithm is also provided below based on a specific data set.

Example

Here, we show the free energy calculation (at 42°C, without formamide) for the duplex in Figure S4A, which has studied and non-studied mismatch conformations. This duplex has 24 quadruplets to be considered, the first of which starts with the first nearest neighbor (TA/AT) and the last with the last nearest neighbor (AT/TA). Two space holder mismatch pairs (X/Y) are added to the right, so that the last two nearest neighbors can be included in two quadruplets of Class 1 (Table S2) at positions 23 and 24.

The free energy is calculated according to Equation S1, with q = 24. The expansion of the summation term is shown by Equation S2, where each quadruplet and its sequence for both strands are in the subscript and its class is indicated in parenthesis according to Table S2. Mismatches are underlined. Numerical values for all free energy components are shown in Equation S4. In this example, there is relaxation at both terminals as indicated by the crossed terms. The first four free energy values add up to a positive value, and hence they are removed, since the overall free energy is more negative without them. Likewise, elimination of the last six terms also makes the overall value more negative. Thus, the duplex with minimum free energy is predicted to have dangling terminals with five and six base pairs as depicted in Figure S4B. The minimum free energy calculation in Equation S3 is graphically represented in Figure S4C.

$$\Delta G^{o}_{duplex} = \Delta G^{o}_{1,\mathrm{TAA}\underline{\mathbf{C}}/\mathrm{ATT}\underline{\mathbf{T}}}\ \text{(Class 1)} + \Delta G^{o}_{2,\mathrm{AA}\underline{\mathbf{C}}\mathrm{G}/\mathrm{TT}\underline{\mathbf{T}}\mathrm{C}}\ \text{(Class 1)} + \Delta G^{o}_{3,\mathrm{A}\underline{\mathbf{C}}\mathrm{GC}/\mathrm{T}\underline{\mathbf{T}}\mathrm{CG}}\ \text{(Class 2)} + \Delta G^{o}_{4,\underline{\mathbf{C}}\mathrm{GCG}/\underline{\mathbf{T}}\mathrm{CGC}}\ \text{(Class 9)} + \Delta G^{o}_{5,\mathrm{GCGC}/\mathrm{CGCG}}\ \text{(Class 1)} + \Delta G^{o}_{6,\mathrm{CGCG}/\mathrm{GCGC}}\ \text{(Class 1)} + \Delta G^{o}_{7,\mathrm{GCGA}/\mathrm{CGCT}}\ \text{(Class 1)} + \Delta G^{o}_{8,\mathrm{CGAT}/\mathrm{GCTA}}\ \text{(Class 1)} + \Delta G^{o}_{9,\mathrm{GATG}/\mathrm{CTAC}}\ \text{(Class 1)} + \Delta G^{o}_{10,\mathrm{ATG}\underline{\mathbf{A}}/\mathrm{TAC-}}\ \text{(Class 1)} + \Delta G^{o}_{11,\mathrm{TG}\underline{\mathbf{AT}}/\mathrm{AC-}\underline{\mathbf{C}}}\ \text{(Class 1)} + \Delta G^{o}_{12,\mathrm{G}\underline{\mathbf{AT}}\mathrm{C}/\mathrm{C-}\underline{\mathbf{C}}\mathrm{G}}\ \text{(Class 5)} + \Delta G^{o}_{13,\underline{\mathbf{AT}}\mathrm{CG}/\mathrm{-}\underline{\mathbf{C}}\mathrm{GC}}\ \text{(Class 10)} + \Delta G^{o}_{14,\underline{\mathbf{T}}\mathrm{CGA}/\underline{\mathbf{C}}\mathrm{GCT}}\ \text{(Class 9)} + \Delta G^{o}_{15,\mathrm{CGAA}/\mathrm{GCTT}}\ \text{(Class 1)} + \Delta G^{o}_{16,\mathrm{GAAA}/\mathrm{CTTT}}\ \text{(Class 1)} + \Delta G^{o}_{17,\mathrm{AAA}\underline{\mathbf{C}}/\mathrm{TTT}\underline{\mathbf{C}}}\ \text{(Class 1)} + \Delta G^{o}_{18,\mathrm{AA}\underline{\mathbf{CG}}/\mathrm{TT}\underline{\mathbf{CG}}}\ \text{(Class 1)} + \Delta G^{o}_{19,\mathrm{A}\underline{\mathbf{CGC}}/\mathrm{T}\underline{\mathbf{CGA}}}\ \text{(Class 12)} + \Delta G^{o}_{20,\underline{\mathbf{CGC}}\mathrm{C}/\underline{\mathbf{CGA}}\mathrm{G}}\ \text{(Class 6)} + \Delta G^{o}_{21,\underline{\mathbf{GC}}\mathrm{CA}/\underline{\mathbf{GA}}\mathrm{GT}}\ \text{(Class 10)} + \Delta G^{o}_{22,\underline{\mathbf{C}}\mathrm{CAT}/\underline{\mathbf{A}}\mathrm{GTA}}\ \text{(Class 9)} + \Delta G^{o}_{23,\mathrm{CAT}\underline{\mathbf{X}}/\mathrm{GTA}\underline{\mathbf{Y}}}\ \text{(Class 1)} + \Delta G^{o}_{24,\mathrm{AT}\underline{\mathbf{XX}}/\mathrm{TA}\underline{\mathbf{YY}}}\ \text{(Class 1)} + \Delta G^{o}_{ini} \qquad \text{(S2)}$$

$$\Delta G^{o}_{duplex} = \cancel{(-0.09 - 0.14 + 1.49 + 0.0)}^{0} - 0.81 - 0.49 - 0.81 - 0.49 - 0.43 - 0.21 - 0.32 + 1.0 + 0.0 + 0.0 - 0.49 - 0.43 - 0.14 - 0.14 + \cancel{(0.0 + 1.0 + 0.0 + 0.0 - 0.32 - 0.21)}^{0} + 1.96 = -1.80 \qquad \text{(S3)}$$

Validation

The algorithm described here can be used for the free energy calculation of any duplex. For duplexes composed of conformations studied in Results section, validation of formamide simulations has already been provided. The objective of this section is to validate the algorithm for duplexes with complex conformations that were not analyzed in Results. Since most oligonucleotide duplexes with complex conformations have low free energy values, and hence low melting points, the approach based on the distance between theoretical and experimental curves (see Results) is not useful for the validation here. Instead, we verify the calculations with a simple method depicted in Figure S4D, using experimental results from all *Rhodobacter sphaeroides* probes (TileR set, Table 1) that formed complex conformations with *Escherichia coli* target. According to this, signal intensities obtained from a hybridization at 15% formamide are consistent with the melting points predicted using free energy values calculated with the quadruplet algorithm, thus verifying the usefulness of approximations in Table S2.

ProbeMelt

The algorithm and thermodynamic parameters developed in this study were incorporated into a computational tool named ProbeMelt. This tool is free and accessible online at http://DECIPHER.cee.wisc.edu. In addition, the program, source code, and associated documentation are available (as part of the DECIPHER package for the R programming language) for download under the terms of the GNU General Public License. This enables the user to integrate the ProbeMelt algorithm and dataset into their own standalone probe design tool.
